# Synthesis of ^11^C-Labelled Ureas by Palladium(II)-Mediated Oxidative Carbonylation

**DOI:** 10.3390/molecules22101688

**Published:** 2017-10-10

**Authors:** Sara Roslin, Peter Brandt, Patrik Nordeman, Mats Larhed, Luke R. Odell, Jonas Eriksson

**Affiliations:** 1 Organic Pharmaceutical Chemistry, Department of Medicinal Chemistry, BMC, Uppsala University, Box 574, SE-751 23 Uppsala, Sweden; peter.brandt@orgfarm.uu.se (P.B.); patrik.nordeman@akademiska.se (P.N.); luke.odell@orgfarm.uu.se (L.R.O.); jonas.p.eriksson@akademiska.se (J.E.); 2 Science for Life Laboratory, Department of Medicinal Chemistry, BMC, Uppsala University, Box 574, SE-751 23 Uppsala, Sweden; mats.larhed@orgfarm.uu.se

**Keywords:** carbon-11, ^11^C-labelling, urea, carbonylation, positron emission tomography, carbon monoxide

## Abstract

Positron emission tomography is an imaging technique with applications in clinical settings as well as in basic research for the study of biological processes. A PET tracer, a biologically active molecule where a positron-emitting radioisotope such as carbon-11 has been incorporated, is used for the studies. Development of robust methods for incorporation of the radioisotope is therefore of the utmost importance. The urea functional group is present in many biologically active compounds and is thus an attractive target for incorporation of carbon-11 in the form of [^11^C]carbon monoxide. Starting with amines and [^11^C]carbon monoxide, both symmetrical and unsymmetrical ^11^C-labelled ureas were synthesised via a palladium(II)-mediated oxidative carbonylation and obtained in decay-corrected radiochemical yields up to 65%. The added advantage of using [^11^C]carbon monoxide was shown by the molar activity obtained for an inhibitor of soluble epoxide hydrolase (247 GBq/μmol–319 GBq/μmol). DFT calculations were found to support a reaction mechanism proceeding through an ^11^C-labelled isocyanate intermediate.

## 1. Introduction

Positron emission tomography (PET) is a non-invasive imaging technique used to visualise and study biological processes in vivo by use of a molecular probe, a PET tracer, where a positron-emitting radioisotope has been incorporated. PET has found extensive use in clinical applications such as oncology, neurology and cardiology [1,2,3]. PET also serves as a useful technique in drug development, where PET offers the possibility to study the distribution, kinetics and target occupancy of potential drugs in vivo [4,5,6].

A radioisotope commonly used in PET is carbon-11 (^11^C), with a half-life of 20.4 min. The natural abundance of carbon in biologically active molecules makes carbon-11 an appealing isotope to incorporate in PET tracers. The short half-life offers the possibility to perform several scans in one patient during a day but it also puts time-restraints on the production of the ^11^C-labelled PET tracer. Efficient incorporation of carbon-11 is therefore a key step for a successful production. The labelling reaction should preferably be performed as the last step in the synthetic route and be a fast, high-yielding and robust method.

Carbon-11 can be incorporated in the PET tracer via a carbonylation reaction using [^11^C]carbon monoxide ([^11^C]CO). There are numerous carbonyl-containing biologically active molecules, thus the ^11^C-carbonylative labelling reaction has great potential in PET tracer development but the minute amounts (typically 10–100 nmol), the short physical half-life and the low solubility of [^11^C]CO in organic solvents pose particular challenges [7,8,9]. To achieve sufficiently fast reaction kinetics, high reagent concentrations are needed, something that can be accomplished by confining the [^11^C]CO in low volume reaction vessels [8]. However, conventional bubbling of [^11^C]CO into the reaction mixture in this setting typically results in low recovery due to the low solubility of [^11^C]CO and efficient purging of the gas phase as the carrier gas is vented. This problem has been approached in different ways, for example using high-pressure autoclave reactors [10,11,12], [^11^C]CO-trapping agents [13,14,15,16,17,18], microfluidic reactors [19,20,21] and an ambient pressure system [22]. The ambient pressure system, developed by Eriksson et al., uses xenon as a carrier gas instead of helium or nitrogen. Most of the carrier gas is absorbed by the reaction solvent, thus precluding the need for high-pressure autoclave reactors or [^11^C]CO-trapping reagents.

**Figure 1:**
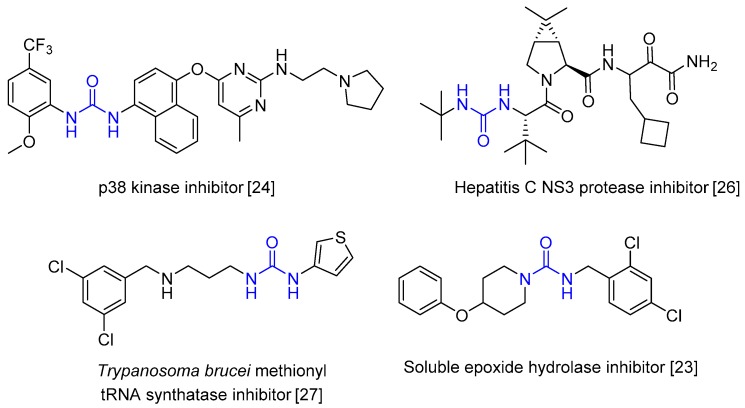
Biologically active ureas.

The urea moiety is a structural motif with a long history in medicinal chemistry and is found in various drugs and biologically active compounds [23]. By offering possibilities for hydrogen bonding, modulation of physicochemical properties and unique binding modes, therapeutic agents acting as inhibitors at targets such as protein kinases, Hepatitis C NS3 protease and methionyl–tRNA synthetase all contain the urea functional group (Figure 1) [23,24,25,26]. With the incorporation of the urea motif in aspiring drugs, there is a rationale for developing a simple and reliable method for incorporation of carbon-11 into the urea functional group.

[^11^C]Urea and ^11^C-labelled urea-derivatives have traditionally been synthesised from [^11^C]phosgene ([^11^C]COCl_2_) [27,28,29,30,31] but also from [^11^C]cyanide [32,33,34], [^11^C]carbon dioxide ([^11^C]CO_2_) [35,36,37,38,39,40,41] and [^11^C]CO [14,42,43,44,45,46,47,48,49]. The different methods come with their own unique limitations. Production of [^11^C]COCl_2_ is rather complicated, and the method is burdened by low product molar activities (A_m_) and is less suited for the labelling of unsymmetrical ureas. [^11^C]HCN also suffers from low A_m_ and the production requires a series of chemical transformations, hence the long reaction times. Methods utilising [^11^C]CO_2_ and [^11^C]CO offer improved A_m_ and fewer or no subsequent chemical transformations. Cyclotron-produced [^11^C]CO_2_ can be utilised directly but fixation agents and drying agents are needed in addition to the amine/amines to be incorporated in the ^11^C-labelled urea. Great care must be taken to make sure that all agents used are freed of atmospheric CO_2_ to avoid isotopic dilution and reduced A_m_. The A_m_ reached with [^11^C]CO_2_-fixation methods have been in the range of 25–148 GBq/μmol [36,37,38,41,50]. High A_m_ is of particular importance when imaging a less abundant target, especially in the central nervous system [51,52]. 

Different approaches have been employed for the synthesis of ^11^C-labelled ureas from [^11^C]CO and amines. The first method published was a selenium-mediated carbonylation for the synthesis of a number of symmetrical and unsymmetrical ^11^C-labelled ureas, where secondary amines were difficult to employ [42]. Rhodium(I) has mainly been used in the synthesis of unsymmetrical ^11^C-labelled ureas and necessitates the use of an azide as precursor, which, according to Doi et al., converts to a nitrene intermediate and subsequently to the ^11^C-labelled isocyanate when reacting with [^11^C]CO. [43,44,45,46,47,48]. In contrast to the case with Rh(I), palladium(II)-mediated ^11^C-urea syntheses can use amines as sole precursors. Kealey et al. reported the use of a Cu(I)scorpionate complex for the trapping of [^11^C]CO for a Pd(II)-mediated formation of symmetrical and unsymmetrical ^11^C-labelled ureas [49]. Primary, aliphatic amines performed well as substrates, whereas anilines were found to be more challenging. Since no ^11^C-labelled ureas were isolated nor any A_m_ determined, the practical utility of the method was difficult to assess.

To address some of the issues with the related methods and to improve the access to ^11^C-labelled ureas, we here report on Pd(II)-mediated oxidative ^11^C-carbonylation of amines for the synthesis of symmetrical and unsymmetrical ^11^C-labelled ureas. The developed protocol utilised [^11^C]CO, with xenon as a carrier gas, a palladium source and amines for the isolation of 14 ^11^C-labelled ureas. Additionally, to demonstrate the advantage of using [^11^C]CO to reduce isotopic dilution, the A_m_ was determined for an inhibitor of soluble epoxide hydrolase (sEH, Figure 1).

## 2. Results and Discussion

The minute amounts of [^11^C]CO available for reaction and the requisite for a finished synthesis within 2–3 half-lives of carbon-11 set the framework for a transition-metal-mediated ^11^C-carbonylation. We have previously used the xenon system for ambient pressure carbonylations and demonstrated its feasibility in synthesising amides [53,54] and sulfonyl carbamates [55]. The report by Kealey et al. as well as our own observations that ^11^C-labelled ureas can form as byproducts in the synthesis of ^11^C-labelled amides, especially when a Pd(II) source is used as a pre-catalyst for the aminocarbonylation, sparked our interest in exploring the Pd(II)-mediated formation of ^11^C-labelled ureas [14].

The investigation into the synthesis of symmetrical ^11^C-labelled ureas began with using benzylamine (**1**) as a model amine and Pd(Xantphos)Cl_2_ as a Pd source [56,57]. Initially, 56% of the [^11^C]CO was converted to non-volatile ^11^C-labelled compounds and the selectivity for [^11^C]-*N*,*N′*-dibenzylurea **2** was >99%, giving a radiochemical yield (RCY) of 55% calculated from the conversion and product selectivity (Table 1, entry 1) [58].

With these encouraging results, the reaction time and temperature were altered in order to improve the conversion. Extending the reaction time to 10 min (entry 2) increased the conversion to 83% and returned a RCY of 82%. Raising the reaction temperature to 150 °C (entry 3) whilst keeping the reaction time at 5 min did not improve the reaction to the same extent as prolonging the reaction time (66% conversion and 63% RCY). When heating the reaction at 150 °C for 10 min (entry 4), the conversion and RCY were further improved to 90% and 87%, respectively. The gain in RCY was minor for entry 4 as compared to entry 2 and we therefore decided to continue with the conditions as in entry 2 to avoid unwanted side reactions caused by the high temperature. In a final experiment, the amount of **1** was lowered to 10 μmol to test whether the conversion of [^11^C]CO and the selectivity could be retained (entry 5). The conversion dropped somewhat but **2** was still obtained in 65% RCY. Further experiments were conducted using 30 μmol of amine.

Next, the scope for symmetrical ^11^C-urea formation was investigated (Table 2). Symmetrical ureas are not as abundant in medicinal chemistry as unsymmetrical ureas. There are, however, examples of bioactive, symmetrical ureas such as [(7-amino(2-naphthyl)sulfonyl]phenylamines derivatives that have been shown to activate insulin receptor tyrosine kinases or sulfonylated naphthyl urea derivatives inhibiting protein arginine methyl transferases [59,60]. The products were isolated by semi-preparative HPLC purification and the radiochemical yields are based on the amount of [^11^C]CO transferred to the reaction vial [58]. Primary, aliphatic amines (**2**, **3** and **4**) were found to be very good substrates and the products were isolated in good radiochemical yields and in >99% radiochemical purity (RCP). The gain in conversion that was seen when increasing the reaction time from 5 min to 10 min manifested itself as a gain in RCY for **2** (41% compared with 65%).

Urea **2** was isolated with 0.44 GBq 34 min after end of nuclide production (EOB) following a 5-min reaction, whereas a 10 min reaction time returned **2** with 0.83–0.88 GBq 33–36 min after EOB. Another example of a symmetrical urea in medicinal chemistry is *N*,*N′*-dicyclohexylurea, which has been identified as a potent inhibitor of sEH (K*_i_* 30 nM), a discovery that sparked interest in the urea as a scaffold for designing sEH inhibitors [61]. Notably, [^11^C]*N*,*N′*-dicyclohexylurea (**4**) was isolated in a high RCY (48%). Aniline was found to be a sluggish substrate and **5** was only isolated in 4% RCY. This is in line with previous studies using either a Pd(II)-source and high concentrations of aniline (90 μmol) or [^11^C]CO_2_-fixation methods using varying amounts of aromatic amines [35,36,49,50]. Rh(I)-mediated synthesis of aromatic ^11^C-labelled ureas has returned higher RCYs, albeit starting from an aromatic azide rather than an aromatic amine [44,45]. When piperidine **6** was used as substrate, the tetra-substituted urea was not detected.

For optimisation of the synthesis of unsymmetrical ^11^C-labelled ureas, **7** was chosen as the model compound (Table 3). Starting from the same conditions as in the synthesis of symmetrical ^11^C-labelled ureas, **7** was obtained in 42% RCY based on a conversion of 53% and a product selectivity of 79% (entry 1) [58]. The ratio of symmetrical (**2**) versus unsymmetrical (**7**) formation was 12:88. Initial optimisation was performed by changing the Pd-source (entries 2–5) [14,49]. Although the Pd-species in entries 2–5 gave higher conversions than Pd(Xantphos)Cl_2_, the product selectivity was lower and consequently the RCYs as well (7–32%). Using DMF as solvent was unfavorable for the product selectivity (entry 6). An investigation of the temperature influence (entries 7 and 8), showed that a lowering of the temperature to 80 °C, which has been used in Rh-based ^11^C-labelled urea syntheses, retained the conversion whilst losing product selectivity [43]. On the other hand, heating the reaction to 150 °C improved the product selectivity but, because of the lower conversion, the yield was in the same range as in entry 1 (41% in entry 8 and 42% in entry 1). The reaction temperature was therefore kept at 120 °C.

Next, increasing the amount of piperidine (**6**) was investigated (entries 9 and 10). Not surprisingly, using five equivalents of **6** gave almost sole formation of unsymmetrical ^11^C-labelled urea **7** (entry 10) whereas having two equivalents of **6** did not improve the product ratio to the same extent (entry 9). The RCY was markedly lower in entry 9 (29%) compared to entry 1 and entry 10 (both 42%), because of both lower conversion and inferior product selectivity. As no improvement in yield was gained by using five equivalents of **6**, the reaction time was altered next. Heating the reaction for 10 min enhanced the conversion, the product selectivity and the ratio of **2** to **7** formed, with a 60% RCY (entry 11). A final experiment, with Pd(PPh_3_)_4_, supported the reaction to be Pd(II)-mediated as neither **2** nor **7** formed with the Pd(0)-source. The conditions in entry 11 were continued with for investigation of the scope for synthesis of unsymmetrical ^11^C-labelled ureas, including sEH inhibitor **19** (Table 4).

Compounds **7**–**19** were isolated by semi-preparative HPLC and the radiochemical yields are based on the amount of [^11^C]CO transferred to the reaction vial [58]. Aliphatic amines were isolated in good RCYs ranging from 12% to 41% (**7**–**10**). Another demonstration of the gain in measured radioactivity of the product with a prolonged reaction time is seen for **7**, where a 5 min reaction time led to a RCY of 17% and 0.15 GBq isolated 37 min from EOB, whilst the 10 min reaction time gave 41% in RCY and 0.47–0.57 GBq isolated after 36–45 min from EOB. 2-(2-Aminoethyl)pyridine proved to be a challenging substrate and **9** was isolated in 12% RCY. Here, an unknown ^11^C-labelled byproduct formed along with product **9**. The byproduct was not observed when amine **6** was removed. In the synthesis of **10**, steric hindrance was introduced in the primary amine, which resulted in a lower RCY compared to **7** and **8**.

A set of aniline derivatives were also synthesised (**11**–**14**) and, similar to **5**, were found to be less reactive than their aliphatic counterparts and were isolated in RCYs varying from 5% to 12%. In the reaction with unfunctionalised aniline, an impurity was present after the purification and **11** could only be isolated with 80% RCP. When three equivalents of aniline were used, the RCY increased from 7% to 12% and the RCP reached 97%. Substitution in the 4-position of the aromatic ring was explored and methoxy- and fluoro-substituents were equally tolerated (**12** and **13**, 8–9%), whereas the nitro group gave a slightly lower RCY of 5% (**14**). It should be noted that the conversion for the reactions with aniline derivatives were in the range of 60–67% and the product selectivities varied from 33% to 71%. The true outcome of the reaction only became fully apparent after isolation of the respective product. Thus, the radiochemical yields estimated for a non-isolated ^11^C-labelled product should be interpreted with care as it may not fully correspond to the radiochemical yield of the isolated product. Next, cyclic ^11^C-labelled urea **15** was synthesised but isolated in a very low RCY (1%). An unknown ^11^C-labelled byproduct formed during the reaction. This byproduct was not formed when the reference was synthesised (even when using Pd(Xantphos)Cl_2_). ^11^C-Labelled sulfonylureas have been synthesised from the corresponding sulfonyl azide in a Rh(I)-mediated reaction but here, synthesis of ^11^C-labelled sulfonyl ureas **16** and **17** did not result any in isolable product [43]. To probe whether the trace formation of **16** was related to the poor nucleophilicity of aniline, the amine was changed to **6** to aid in the plausible ^11^C-sulfonyl isocyanate formation and subsequent product formation to sulphonylurea **17**. However, as seen in Table 4, this change did not improve the reaction outcome.

Ethanol has been found to increase the conversion of [^11^C]phosgene-derived ammonium [^11^C]isocyanate to [^11^C]urea [31]. Similarly, different alcoholic additives were found to accelerate the conversion of ammonium cyanate to urea [62]. Therefore, 10 equivalents of 1-butanol were added to the reaction mixture of **7**, **8** and **11**–**15**. Compounds **12** and **13**, were isolated with improved RCY (21% and 28%) whereas the RCY of **7** and **8**, synthesised from an aliphatic primary amine and **6**, were not improved (31% and 14%). Hence, the addition of 1-butanol could be most beneficial when a poorly nucleophilic primary amine is used. However, the RCY of **11**, **14** and **15** were not improved thus making the hypothesis of nucleophilicity less certain. Of note is that the ^11^C-labelled byproduct formed during the synthesis of **15**, was isolated in a RCY of 72% when 1-butanol was added. 

Lactams has previously been synthesised under Pd(II)-catalysed conditions, however, with the conditions employed here, in particular the short reaction time and small amount of [^11^C]CO, ^11^C-labelled lactam **18** was not formed [63]. A ^11^C-labelled byproduct was formed, which in part accounts for the relatively high [^11^C]CO-conversion (66%). The byproduct was hypothesised from LC/MS to be [^11^C]*N*-benzyl-*N*-ethylbenzamide, formed by scrambling of a phenyl group from the Pd-ligand. A subsequent synthesis of *N*-benzyl-*N*-ethylbenzamide from benzoic acid and *N*-ethyl benzylamine and matching of LC retention times confirmed the identity. When the reaction mixture was preheated, to aid in dissolution of reactants, [^11^C]*N*-benzyl-*N*-ethylbenzamide was formed in 25% RCY, whereas the RCY dropped to 4% when the reaction mixture was not preheated. Lastly, sEH inhibitor **19** was synthesised in a good 41% RCY [23]. To further demonstrate the utility of the method, with respect to molar activity, was the A_m_ determined for **19** in two experiments. When starting from 17.8 GBq of [^11^C]CO, product **19** was isolated with 1.9 GBq and in a A_m_ of 247 GBq/μmol, 43 min after EOB. In the second experiment, when starting with 12.8 GBq of [^11^C]CO, **20** could be isolated with 2.1 GBq and a A_m_ of 319 GBq/μmol, 41 min from EOB.

**Scheme 1:**
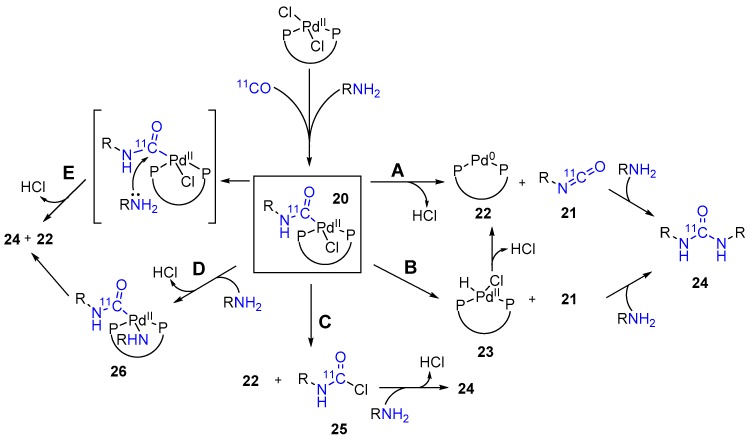
Hypothetical reaction paths for ^11^C-labelled urea formation.

In Scheme 1, hypothetical paths **A**–**E** through which the reaction can proceed are presented. A carbamoylpalladium species has been proposed by Hiwatari et al. to form as an intermediate in the reaction [64]. Thus, hypothesising the same species will form in the present reaction with [^11^C]CO, ^11^C-labelled carbamoylpalladium species **20** can generate ^11^C-labelled isocyanate **21** through the deprotonation of **20** by an external base such as in path **A**. Alternatively, **21** can be formed by a β-elimination to give a hydridopalladium(II) complex **23**, which subsequently can reductively eliminate HCl to yield Pd(0)-complex **22** (path **B**). The elimination depicted in path **A** has found experimental support in kinetic studies where the disappearance of a carbamoylpalladium complex was found to have a first-order dependence on the concentration of a tertiary amine [64]. Attack of a second amine on **21** will furnish product **24**. Carbamoyl chloride **25** is proposed to form in path **C** after a reductive elimination from **20**. As in **A** and **B**, attack of another amine gives **24**. In paths **D** and **E**, the second amine reacts directly with **20**, either through initial coordination to Pd (complex **26**, path **D**) or through a direct attack on the carbonyl (path **E**).

Path **A**, and path **B** to some extent, could explain the low RCY of **5** if the weakly basic aniline cannot deprotonate **20** and/or is slow to react with **21**. However, the low RCYs of **11**–**14** where **6** with ease should be able to both deprotonate **20** and subsequently trap **21** to form product cannot be rationalised in the same way. Although paths **C**–**E** are feasible on paper, previous experimental studies support an isocyanate intermediate, i.e., path **A** or **B**, on the basis that secondary amines does not furnish the corresponding tetra-substituted urea under the conditions explored [63,64,65]. Further support of path **A** or **B** is that the ratio of symmetric ^11^C-labelled urea **2** to unsymmetrical ^11^C-labelled urea **7** was here found to be approximately 1:9 and the same ratio was found when **1** and **6** were allowed to react with benzyl isocyanate. Furthermore, when performing the reaction with only 4-fluoroaniline and Pd(Xantphos)Cl_2_, was the presence of [^11^C]4-fluorophenyl isocyanate confirmed by analytical HPLC and co-injection with 4-fluorophenyl isocyanate. Amine **6** was thereafter added and the reaction was heated for another 10 min. A second analysis revealed that **13** had formed. However, the mere presence of the ^11^C-labelled isocyanate does not preclude the reaction from proceeding through an alternative mechanism. 

**Scheme 2:**
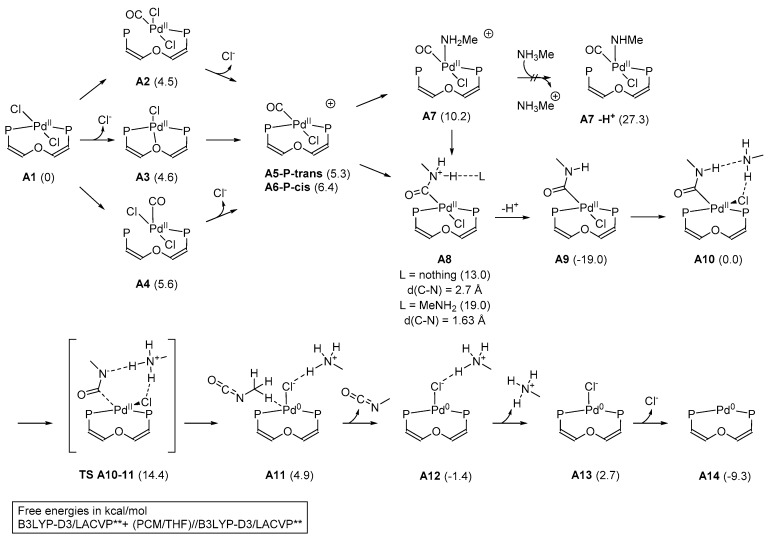
Calculated free energies (kcal/mol) of intermediates along path **A** in Scheme 1 showing that the path is energetically feasible.

To lend further support to path **A** in Scheme 1, DFT-calculations were performed for the urea formation from CO and *N*-methyl amine. Although a solvent model for THF was used, the accuracy of the energies will suffer from the variation in charge for the palladium complexes on the reaction path. However, it was reasoned that the calculations would be accurate enough to give a rough estimate of the stability of intermediates along the reaction path (Scheme 2).

As shown in Scheme 2, addition of CO to the initial palladium dichloro complex **A1** does not involve any dramatic energy changes and addition of a primary amine is also feasible. Deprotonation of the palladium-bound amine is not likely, however. Instead, the amine can attack the carbonyl directly forming a labile *N*-protonated carbamoyl species. In the gas-phase, the C–N bond varies between 2.07 Å and 2.70 Å depending on whether the cation can be stabilised by the phenylphosphine or not. Upon addition of a stabilising amine to **A8**, the C–N bond shortens to 1.63 Å. The carbamoyl species **A9**, formed by deprotonation of **A8**, is the most stable intermediate on the reaction path. Base-assisted deprotonation of this species leads to the formation of an isocyanate and the palladium(0)-complex **A14** in accordance with the first-order dependence on the concentration of a tertiary amine reported by Hiwatari et al. [64].

## 3. Materials and Methods

### 3.1. General Chemistry Information

Reagents and solvents were obtained from Sigma-Aldrich (St. Louis, MO, USA) and Fischer (Pittsburgh, PA, USA) and used without further purification. Thin layer chromatography (TLC) was performed on aluminium sheets pre-coated with silica gel 60 F_254_ (0.2 mm, Merck KGaA, Darmstadt, Germany). Column chromatography was performed using silica gel 60 (40–63 μm, Merck KGaA, Darmstadt, Germany). Carbon-11 was prepared by the ^14^N(p,α)^11^C nuclear reaction using 17 MeV protons produced by a Scanditronix MC-17 Cyclotron at PET Centre, Uppsala University Hospital, and obtained as [^11^C]carbon dioxide. The target gas used was nitrogen (AGA Nitrogen 6.0) containing 0.05% oxygen (AGA Oxygen 4.8). The [^11^C]CO_2_ was transferred to the low-pressure xenon system in a stream of helium gas and concentrated before reduction to [^11^C]CO, over zinc heated to 400 °C [22,54]. A column with Ascarite was used to trap residual [^11^C]CO_2_. Before transferring the concentrated [^11^C]CO into the reaction vial, through a transfer needle placed in the capped reaction vial, the carrier gas was changed from helium to xenon (>99.9%, 1.5 mL/min). No venting needle was required during the transfer and, when completed, the transfer needle was removed and the reaction vial was lowered into a heating block. Analytical reversed phase HPLC-MS was performed on a Dionex Ultimate 3000 system using 0.05% HCOOH in water and 0.05% HCOOH in acetonitrile as mobile phase with MS detection, equipped with a C18 (Phenomenex Kinetex SB-C18 (4.8 × 50 mm)) column using a UV diode array detector. Purity determinations were performed using C18 (Phenomenex Kinetex SB-C18 (4.8 × 50 mm)) column and Biphenyl (Phenomenex Kinetex Biphenyl (2.6 μm, 4.6 × 50 mm) column, with UV detection at 214 nm or 254 nm. Semi-preparative reversed phase HPLC was performed on a Gilson–Finnigan ThermoQuest AQA system equipped with Gilson GX-271 system equipped with a C18 (Macherey-Nagel Nucleodur HTec (5 μm, 125 × 21 mm)) using 0.1% TFA in water and 0.1% TFA in acetonitrile as eluent. Purifications of ^11^C-labelled ureas were performed on a VWR La Prep Sigma system with a LP1200 pump, 40D UV detector, a Bioscan flowcount radiodetector. The identities, concentration and radiochemical purities of the purified ^11^C-labeled ureas were determined with either a VWR Hitachi Elite LaChrom system (L-2130 pump, L-2200 autosampler, L-2300 column oven, L-2450 diode array detector in series with a Bioscan β^+^-flowcount radiodetector) or an Elite LaChrom VWR International (LaPrep P206 pump, an Elite LaChrom L-2400 UV detector in series with a Bioscan β^+^-flowcount detector), 8.1 mM ammonium carbonate (aq.) and acetonitrile mobile phase and Merck Chromolith Performance RP-18e column (4.6 × 100 mm) using isotopically unmodified compounds as references. NMR spectra were recorded on a Varian Mercury plus spectrometer (^1^H at 399.8 MHz, ^13^C at 100.5 MHz and ^19^F at 376.5 MHz) at ambient temperature. Chemical shifts (δ) are reported in ppm, indirectly referenced to tetrametylsilane (TMS) via the residual solvent signal (^1^H: CHCl_3_ δ 7.26, CD_2_HOD δ 3.31, (CHD_2_)(CD_3_)SO δ 2.50, (CHD_2_)(CD_3_)CO δ 2.05 ^13^C: CDCl_3_ δ 77.2, CD_3_OD δ 49.0, (CHD_2_)(CD_3_)SO δ 39.5, (CD_3_)_2_CO δ 29.8, 206.3.

### 3.2. Synthesis of Starting Materials

#### 3.2.1. *tert*-Butyl 4-hydroxypiperidine-1-carboxylate [66] CAS: 109384-19-2

4-Hydroxypiperdine (3.1 mmol) was dissolved in THF (10 mL), to which 10% NaOH (aq., 1.5 mL) was added. Di-*tert*-butyl dicarbonate (1.2 equiv.) was slowly added to the reaction mixture. After completion of the reaction, the reaction mixture was extracted with 3 × 15 mL chloroform. The combined organic phases were washed with 15 mL Brine, dried over Na_2_SO_4_, filtered and concentrated in vacuo. Flash column chromatography followed, using 15% ethanol in ethyl acetate as eluent. Isolated as a white solid in 95% purity and used without further purification.^1^H-NMR (400 MHz, CDCl_3_) δ 3.91–3.76 (m, 3H), 3.07–2.96 (m, 2H), 1.89–1.80 (m, 2H), 1.68 (d, *J* = 4.2 Hz, 1H), 1.50–1.46 (m, 1H), 1.45 (s, 9H), 1.43–1.39 (m, 1H).

#### 3.2.2. *tert*-Butyl 4-phenoxypiperidine-1-carboxylate [66] CAS: 155989-69-8

*tert*-Butyl 4-hydroxypiperidine-1-carboxylate (3.1 mmol), phenol (1.0 equiv.) and PPh_3_ (1.0 equiv.) were dissolved in anhydrous THF (16 mL) before addition of diisopropyl azodicarboxylate (1.0 equiv.). The reaction mixture was stirred at room temperature for 18 h. The reaction mixture was concentrated before purification with flash column chromatography, using 7:1 *i*-hexane:ethyl acetate as eluent. Isolated as a white solid (498 mg, 59%). *R*_f_ = 0.47 (7:1 *i*-hexane:ethyl acetate). ^1^H-NMR (400 MHz, CDCl_3_) δ 7.31–7.27 (m, 2H), 6.99–6.88 (m, 3H), 4.46 (hept, *J* = 3.5 Hz, 1H), 3.75–3.64 (m, 2H), 3.39–3.26 (m, 2H), 1.98–1.86 (m, 2H), 1.83–1.69 (m, 2H), 1.47 (s, 9H). ^13^C-NMR (101 MHz, CDCl_3_) δ 157.3, 155.0, 129.7, 121.2, 116.3, 79.7, 72.3, 40.9, 30.7, 28.5. MS-EI *m*/*z* 277.1.

#### 3.2.3. 4-Phenoxypiperidine [66] CAS: 3202-33-3

*tert*-Butyl 4-phenoxypiperidine-1-carboxylate (453 mg) was dissolved in dichloromethane (10 mL). TFA (1.4 mL) was added and the mixture was stirred at room temperature for 2.5 h. The reaction mixture was concentrated and re-dissolved in dichloromethane (20 mL), followed by washing with 1.5 M NaOH (20 mL). The water phase was extracted with 2 × 20 mL DCM. The combined organic phases were dried over Na_2_SO_4_, filtered and concentrated. Purification with flash column chromatography using 5% MeOH and 1% triethylamine (TEA) in DCM Product was isolated as a white solid (290 mg, 96%). *R*_f_ = 0.18 (5% MeOH, 1% TEA in DCM). ^1^H-NMR (400 MHz, CDCl_3_) δ 7.30–7.25 (m, 2H), 6.96–6.89 (m, 3H), 4.39 (hept, *J* = 3.9 Hz, 1H), 3.20–3.12 (m, 2H), 2.80–2.72 (m, 2H), 2.24 (br. s, 1H), 2.08–1.97 (m, 2H), 1.75–1.65 (m, 2H). ^13^C-NMR (101 MHz, CDCl_3_) δ 157.4, 129.6, 121.0, 116.3 73.1, 43.8, 32.2. HPLC purity > 99%.

### 3.3. Synthesis of Reference Compounds

#### 3.3.1. General Procedure for Synthesis of Reference Compounds via an Oxidative Carbonylation

The reaction was performed in a double-chamber system [67,68]. Amine/amines, Pd(OAc)_2_ (0.05 equiv.) and Cu(OAc)_2_ (0.5 equiv.) were added to the reaction chamber and dissolved in 1,4-dioxane (2 mL). In the CO-chamber was Mo(CO)_6_ (200 mg) dissolved in 1,4-dioxane (2 mL). After capping of the system, 1,8-diazabicyclo[5.4.0]undec-7-ene was added to the CO-chamber. The double-chamber system was positioned in a Dry–Syn heating block and heated to 95 °C. After completion of the reaction, the reaction mixture was filtered through a short silica plug before flash column chromatography.

#### 3.3.2. *N*,*N′*-Dibenzylurea [69] CAS: 1466-67-7

Synthesised according to the general procedure from benzylamine (1 mmol). Purified with flash column chromatography, using 1% MeOH and 1% AcOH in DCM as eluent. Isolated as a white powder (74 mg, 31%). *R*_f_ = 0.23 (1% MeOH and 1% acetic acid in DCM). ^1^H-NMR (400 MHz, DMSO-*d*_6_) δ 7.34–7.18 (m, 10H), 6.46 (t, *J* = 6.1 Hz, 2H), 4.23–4.20 (m, 4H). ^13^C-NMR (101 MHz, CDCl_3_ and MeOD) δ 159.1, 139.3, 128.4, 127.2, 127.0, 44.0. MS-ESI [M + H^+^] = *m*/*z* 241.3. HPLC purity > 99%. 

#### 3.3.3. *N*-(2-(Pyridin-2-yl)ethyl)piperidine-1-carboxamide CAS: 1710806-84-0

Synthesised according to the general procedure from 2-(2-aminoethyl)pyridine (1.0 mmol) and piperidine (3 equiv.). Purified with flash column chromatography, using 1% MeOH and 1% AcOH in DCM as eluent. Isolated as a solid (49 mg, 21%). *R*_f_ = 0.16 (ethyl acetate + 1% TEA). ^1^H-NMR (400 MHz, (CD_3_)_2_CO) δ 8.51−8.47 (m, 1H), 7.67 (td, *J* = 7.7, 1.9 Hz, 1H), 7.24 (d, *J* = 7.8 Hz, 1H), 7.20−7.15 (m, 1H), 6.04 (br. s, 1H), 3.54−3.47 (m, 2H), 3.33−3.27 (m, 4H), 2.94 (t, *J* = 7.0 Hz, 2H), 1.60−1.53 (m, 2H), 1.49−1.41 (m, 4H). ^13^C-NMR (101 MHz, (CD_3_)_2_CO) δ 161.2, 158.3, 150.0, 137.1, 124.0, 122.1, 45.4, 41.3, 41.2, 39.1, 39.0, 26.5, 25.4. HRMS calc: 234.1606 found: 234.1614. HPLC purity > 99%. 

#### 3.3.4. 3,4-Dihydroquinazolin-2(1H)-one [70] CAS: 66655-67-2

Synthesised according to the general procedure from 2-aminobenzylamine (1.5 mmol). Purified twice with flash column chromatography, using 5% MeOH, 1% TEA in DCM and 1% TEA in ethyl acetate, respectively, followed by semi-preparative purification. Isolated as a white powder (26 mg, 12%). *R*_f_ = 0.74 (5% MeOH, 1% TEA in DCM). ^1^H-NMR (400 MHz, (CD_3_)_2_SO) δ 8.98 (br. s, 1H), 7.12–7.04 (m, 2H), 6.84 (td, *J* = 7.5, 1.2 Hz, 1H), 6.79–6.73 (m, 2H), 4.29 (s, 2H). ^13^C-NMR (101 MHz, (CD_3_)_2_SO) δ 154.6, 138.1, 127.6, 125.6, 120.9, 118.1, 113.5, 42.5. MS-ESI [M + H^+^] = *m*/*z* 149.1. HPLC purity > 99%. 

#### 3.3.5. 2-Ethylisoindolin-1-one [71] CAS: 23967-95-5

Synthesised according to the general procedure from *N*-ethylbenzylamine (1 mmol). Purified with flash column chromatography, using 3:1 i-hexane:ethyl acetate + 1% TEA. Isolated as a yellow liquid (49 mg, 31%). *R*_f_ = 0.25 (3:1 i-hexane:ethyl acetate + 1% TEA). ^1^H-NMR (400 MHz, CDCl_3_/CD_3_OD) δ 7.77 (d, *J* = 7.5 Hz, 1H), 7.54–7.49 (m, 1H), 7.47–7.41 (m, 2H), 4.40 (s, 2H), 3.64 (q, *J* = 7.3 Hz, 2H), 1.25 (t, *J* = 7.3 Hz, 3H). ^13^C-NMR (101 MHz, CDCl_3_/CD_3_OD) δ 141.4, 132.7, 131.7, 128.4, 123.6, 123.0, 49.8, 37.4, 13.6 (Carbonyl carbon missing). MS-ESI [M + H^+^] = *m*/*z* 162.1. HPLC purity > 99%.

#### 3.3.6. *N*-(2,4-Dichlorobenzyl)-4-phenoxypiperidine-1-carboxamide CAS: 950645-62-2

Synthesised according to the general procedure from 2,4-dichlorobenzylamine (0.43 mmol) and 4-phenoxypiperidine (2 equiv.). Purified with flash column chromatography, using 1% MeOH and 1% AcOH in DCM as eluent, followed by semi-preparative chromatography. Isolated as a white powder (47 mg, 29%). *R*_f_ = 0.53 (5% MeOH, 1% TEA in DCM). ^1^H-NMR (400 MHz, (CD_3_)_2_CO) δ 7.46–7.42 (m, 2H), 7.33 (dd, *J* = 8.2, 2.2 Hz, 1H), 7.31–7.24 (m, 2H), 7.00–6.94 (m, 2H), 6.92 (tt, *J* = 7.3, 1.1 Hz, 1H), 4.62 (tt, *J* = 7.6, 3.7 Hz, 1H), 4.45 (d, *J* = 5.0 Hz, 2H), 3.86–3.75 (m, 2H), 3.41–3.30 (m, 2H), 2.03–1.95 (m, 2H), 1.72–1.61 (m, 2H).^13^C-NMR (101 MHz, (CD_3_)_2_CO) δ 157.6, 157.5, 137.2, 133.3, 132.5, 130.3, 129.6, 128.6, 127.1, 120.8, 116.1, 72.2, 41.6, 41.2, 30.7. HRMS calc: 379.0980 found: 379.0999. HPLC purity > 99%.

#### 3.3.7. General Procedure for the Synthesis of Reference Compounds from an Isocyanate

The isocyanate derivative (1 mmol) was dissolved in DCM (2 mL) and cooled to −10 °C before piperidine (1 equiv.) in DCM (2 mL) was added under N_2_. The reaction was stirred under N_2_ for 30 min and then let to warm room temperature and stir until completion. The reaction mixture was concentrated in vacuo and recrystallised.

#### 3.3.8. *N*-Benzylpiperidine-1-carboxamide [72] CAS: 39531-35-6

Synthesised according to the general procedure from benzyl isocyanate. Recrystallised in petroleum ether and isolated as colourless crystals (87 mg, 40%). *R*_f_ = 0.33 (3:1 *i*-hexane:ethyl acetate + 1% TEA). Melting point: 99–100 °C [73]. ^1^H-NMR (400 MHz, (CD_3_)_2_CO) δ 7.32–7.24 (m, 4H), 7.21–7.16 (m, 1H), 6.27 (s, 1H), 4.35 (d, *J* = 5.8 Hz, 2H), 3.40–3.35 (m, 4H), 1.63–1.55 (m, 2H), 1.53–1.44 (m, 4H). ^13^C-NMR (101 MHz, (CD_3_)_2_CO) δ 158.3, 142.3, 128.9, 128.2, 127.3, 45.6, 44.9, 26.6, 25.4. MS-ESI [M + H^+^] = *m*/*z* 219.4. HPLC purity > 99%.

#### 3.3.9. *N*-Butylpiperidine-1-carboxamide CAS: 1461-79-6

Synthesised according to the general procedure from butyl isocyanate. Recrystallised in petroleum ether and isolated as colourless crystals (150 mg, 81%). *R*_f_ = 0.15 (3:1 *i*-hexane:ethyl acetate + 1% TEA). Melting point: 60–62 °C. ^1^H-NMR (400 MHz, (CD_3_)_2_CO) δ 5.73 (s, 1H), 3.35–3.26 (m, 4H), 3.16–3.09 (m, 2H), 1.60–1.52 (m, 2H), 1.51–1.39 (m, 6H), 1.37–1.24 (m, 2H), 0.88 (t, *J* = 7.3 Hz, 3H). ^13^C-NMR (101 MHz, (CD_3_)_2_CO) δ 158.4, 45.5, 41.0, 40.9, 33.4, 33.4, 26.5, 25.4, 20.7, 14.2. HRMS calc: 185.1654 found: 185.1653. HPLC purity > 99%.

#### 3.3.10. *N*-Isopropylpiperidine-1-carboxamide CAS: 10581-04-1

Synthesised according to the general procedure from isopropyl isocyanate. Recrystallised in petroleum ether and isolated as white crystals (100 mg, 58%). Melting point: 114–121 °C. ^1^H-NMR (400 MHz, (CD_3_)_2_SO) δ 6.02 (d, *J* = 7.6 Hz, 1H), 3.80–3.66 (m, 1H), 3.25–3.20 (m, 4H), 1.56–1.46 (m, 2H), 1.44–1.34 (m, 4H), 1.03 (d, *J* = 6.6 Hz, 6H). ^13^C-NMR (101 MHz, (CD_3_)_2_CO) δ 156.7, 44.2, 41.6, 25.3, 24.2, 23.0. HRMS calc: 171.1497 found: 171.1504. HPLC purity > 99%.

#### 3.3.11. *N*-Phenylpiperidine-1-carboxamide [72] CAS: 2645-36-5

Synthesised according to the general procedure from phenyl isocyanate. Recrystallised in ethyl acetate and isolated as colorless crystals (173 mg, 84%). *R*_f_ = 0.37 (3:1 *i*-hexane:ethyl acetate + 1% TEA). Melting point: 168–171 °C. ^1^H-NMR (400 MHz, (CD_3_)_2_CO) δ 7.84 (br. s, 1H), 7.54–7.49 (m, 2H), 7.23–7.17 (m, 2H), 6.91 (tt, *J* = 7.4, 1.2 Hz, 1H), 3.50–3.44 (m, 4H), 1.67–1.59 (m, 2H), 1.58–1.49 (m, 4H). ^13^C-NMR (101 MHz, (CD_3_)_2_CO) δ 155.7, 141.9, 129.1, 122.4, 120.2, 45.8, 26.6, 25.3. MS-ESI [M + H^+^] = *m*/*z* 205.4. HPLC purity > 99%.

#### 3.3.12. *N*-(4-Methoxyphenyl)piperidine-1-carboxamide CAS: 2645-37-6

Synthesised according to the general procedure from 4-methoxyphenyl isocyanate. Recrystallised in petroleum ether and isolated as colourless crystals (183 mg, 78%). *R*_f_ = 0.49 (3:1 *i*-hexane:ethyl acetate + 1% TEA). Melting point: 124–125 °C. ^1^H-NMR (400 MHz, (CD_3_)_2_CO) δ 7.68 (s, 1H), 7.43–7.37 (m, 2H), 6.82–6.76 (m, 2H), 3.73 (s, 3H), 3.50–3.39 (m, 4H), 1.65–1.58 (m, 2H), 1.57–1.49 (m, 4H). ^13^C-NMR (101 MHz, (CD_3_)_2_CO) δ 155.9, 135.0, 122.1, 122.0, 114.3, 55.6, 45.8, 26.6, 25.3. HRMS calc: 235.1447 found: 235.1450. HPLC purity > 99%. 

#### 3.3.13. *N*-(4-Fluorophenyl)piperidine-1-carboxamide CAS: 60465-12-5

Synthesised according to the general procedure from 4-fluorophenyl isocyanate. Recrystallised in ethyl acetate and isolated as colourless crystals (188 mg, 84%). *R*_f_ = 0.39 (3:1 *i*-hexane:ethyl acetate + 1% TEA). Melting point: 179–184 °C. ^1^H-NMR (400 MHz, (CD_3_)_2_CO) δ 7.90 (br. s, 1H), 7.55–7.48 (m, 2H), 7.02–6.94 (m, 2H), 3.50–3.42 (m, 4H), 1.67–1.58 (m, 2H), 1.57–1.48 (m, 4H). ^13^C-NMR (101 MHz, (CD_3_)_2_CO) δ 158.8 (d, *J* = 238.2 Hz), 155.8, 138.2 (d, *J* = 2.6 Hz), 121.9 (d, *J* = 7.5 Hz), 115.4 (d, *J* = 22.3 Hz), 45.7, 26.6, 25.3. ^19^F-NMR (376 MHz, (CD_3_)_2_CO) δ -119.3 – -130.5. HRMS calc: 223.1247 found: 223.1245. HPLC purity > 99%.

#### 3.3.14. *N*-(4-Nitrophenyl)piperidine-1-carboxamide [63] CAS: 2589-20-0

Synthesised according to the general procedure from 4-nitrophenyl isocyanate. Recrystallised in petroleum ether and ethyl acetate and isolated as yellow crystals (223 mg, 89%). *R*_f_ = 0.39 (3:1 *i*-hexane:ethyl acetate + 1% TEA). Melting point: 159–162 °C. ^1^H-NMR (400 MHz, (CD_3_)_2_CO) δ 8.51–8.47 (m, 1H), 7.67 (td, *J* = 7.7, 1.9 Hz, 1H), 7.24 (d, *J* = 7.8 Hz, 1H), 7.20–7.15 (m, 1H), 6.04 (br. s, 1H), 3.54–3.47 (m, 2H), 3.33–3.27 (m, 4H), 2.94 (t, *J* = 7.0 Hz, 2H), 1.60–1.53 (m, 2H), 1.49–1.41 (m, 4H). ^13^C-NMR (101 MHz, (CD_3_)_2_CO) δ 161.2, 158.3, 150.0, 137.1, 124.0, 122.1, 45.4, 41.3, 41.2, 39.1, 39.0, 26.5, 25.4. MS-ESI [M + H^+^] = *m*/*z* 250.2. HPLC purity > 99%.

#### 3.3.15. *N*-Tosylpiperidine-1-carboxamide CAS: 23730-08-7

Synthesised according to the general procedure from *p*-toluenesulfonyl isocyanate. Recrystallised in petroleum ether and isolated as white crystals (144 mg, 51%). *R*_f_ = 0.85 (5% MeOH, 1% TEA in DCM). Melting point: 133–137 °C. ^1^H-NMR (400 MHz, (CD_3_)_2_SO) δ 10.75 (br. s, 1H), 7.76 (d, *J* = 8.2 Hz, 2H), 7.36 (d, *J* = 8.1 Hz, 2H), 3.29–3.23 (m, 4H), 2.38 (s, 3H), 1.54–1.46 (m, 2H), 1.43–1.35 (m, 4H). ^13^C-NMR (101 MHz, (CD_3_)_2_CO) δ 151.4, 143.4, 138.7, 129.0, 128.2, 44.9, 25.6, 24.1, 20.6. HRMS calc: 283.1116 found: 283.1103. HPLC purity > 99%.

### 3.4. General Procedure for the Synthesis and Analysis of ^11^C-Labelled Ureas

Pd(Xantphos)Cl_2_ (4 μmol) and amine/amines (30 μmol) were added to an oven-dried, conical glass vial followed by freshly distilled tetrahydrofuran (400 μL). [^11^C]CO was transferred to the capped reaction vial and the radioactivity was measured to determine the starting amount of [^11^C]CO. The reaction was heated at 120 °C for 10 min. When finished, the radioactivity was measured to confirm that no radioactive material had escaped during heating. The vial was purged with N_2_ to remove unreacted [^11^C]CO and, possibly, volatile labelled compounds formed during the reaction. The radioactivity was measured after the purge followed by either analytical HPLC for product selectivity determination or semi-preparative HPLC purification for ^11^C-labelled product isolation. Purification was performed using either column C1 = Phenomenex Kinetex C18 (5 μm, 150 × 10.0 mm) column, C2 = Reprosil–Pur Basic C18 (5 μm, 150 × 10.0 mm) column or C3 = Gemini NX C18 (5 μm, 250 × 10.0 mm) column with ammonium formate buffer 50 mM (pH 3.5) (A) and acetonitrile (B) as eluents. Run time was 20 min with flow 5 mL/min followed by flushing the column with 100% B. After isolation and a final radioactivity measurement of the ^11^C-labelled product, an aliquot was analysed to determine radiochemical purity and the identity of the ^11^C-labelled product was confirmed using the isotopically unmodified product as reference. The analytical method was the same for all compounds, 10–90% acetonitrile in 10 min (flow 2 mL/min). Molar activity determinations were based on a calibration curve, constructed with isotopically unmodified **20**. For full definitions and calculations, see the supporting materials.

#### 3.4.1. [*carbonyl*-^11^C]*N*,*N′*-Dibenzylurea **2**

Synthesised according to the general procedure (three experiments). (1) Purification method: 40% B. Column: C1. Starting from 3.4 GBq, 0.88 GBq was isolated at 33 min from EOB; (2) Purification method: 35% B Column: C1. Starting from 3.4 GBq, 0.83 GBq was isolated at 36 min from EOB; (3) Purification method: 45% B Column: C3. Starting from 2.7 GBq, 0.44 GBq was isolated at 34 min from EOB. Analytical HPLC R_t_ = 5.1 min.

#### 3.4.2. [*carbonyl*-^11^C]*N*,*N′*-Dipropylurea **3**

Synthesised according to the general procedure (two experiments). (1) Purification method: 0–70% B Column: C1. Starting from 2.2 GBq, 0.24 GBq was isolated at 42 min from EOB; (2) Purification method: 0–70% B Column: C1. Starting from 6.7 GBq, 0.62 GBq was isolated at 53 min from EOB. Analytical HPLC R_t_ = 2.6 min.

#### 3.4.3. [*carbonyl*-^11^C]*N*,*N′*-Dicyclohexylurea **4**

Synthesised according to the general procedure (two experiments). (1) Purification method: 40% B Column: C1. Starting from 3.4 GBq, 0.53 GBq was isolated at 37 min from EOB; (2) Purification method: 35% B Column: C1. Starting from 3.8 GBq, 0.60 GBq was isolated at 41 min from EOB. Analytical HPLC R_t_ = 5.6 min.

#### 3.4.4. [*carbonyl*-^11^C]*N*,*N′*-Diphenylurea **5**

Synthesised according to the general procedure (two experiments). (1) Purification method: 35% B Column: C1. Starting from 3.0 GBq, 0.028 GBq was isolated at 50 min from EOB; (2) Purification method: 35% B Column: C1. Starting from 6.7 GBq, 0.059 GBq was isolated at 43 min from EOB. Analytical HPLC R_t_ = 5.5 min.

#### 3.4.5. [*carbonyl*-^11^C]*N*-Benzylpiperidine-1-carboxamide **7**

Synthesised according to the general procedure (four experiments). (1) Purification method: 35% B Column: C1. Starting from 4.9 GBq, 0.47 GBq was isolated at 45 min from EOB; (2) Purification method: 35% B Column: C1. Starting from 3.0 GBq, 0.57 GBq was isolated at 36 min from EOB; (3) 5 min reaction time. Purification method: 35% B Column: C3. Starting from 2.6 GBq, 0.15 GBq was isolated at 37 min from EOB; (4) 10 equiv. of 1-butanol added. Purification method: 35% B Column: C3. Starting from 3.2 GBq, 0.29 GBq was isolated at 42 min from EOB. Analytical HPLC R_t_ = 4.9 min.

#### 3.4.6. [*carbonyl*-^11^C]*N*-Butylpiperidine-1-carboxamide **8**

Synthesised according to the general procedure (three experiments). (1) Purification method: 20–50% Column: C1. Starting from 3.7 GBq, 0.25 GBq was isolated at 44 min from EOB; (2) Purification method: 20–50% Column: C1. Starting from 2.9 GBq, 0.17 GBq was isolated at 46 min from EOB; (3) 10 equiv. of 1-butanol added. Purification method: 20–50% Column: C3. Starting from 2.8 GBq, 0.093 GBq was isolated at 49 min from EOB. Analytical HPLC R_t_ = 4.5 min.

#### 3.4.7. [*carbonyl*-^11^C]*N*-(2-(Pyridin-2-yl)ethyl)piperidine-1-carboxamide **9**

Synthesised according to the general procedure (two experiments). (1) Purification method: 20–50% B Column: C1. Starting from 3.3 GBq, 0.14 GBq was isolated at 36 min from EOB; (2) Purification method: 20% B Column: C1. Starting from 3.7 GBq, 0.13 GBq was isolated at 39 min from EOB. Analytical HPLC R_t_ = 3.8 min.

#### 3.4.8. [*carbonyl*-^11^C]*N*-Isopropylpiperidine-1-carboxamide **10**

Synthesised according to the general procedure (two experiments). (1) Purification method: 15–50% B Column: C1. Starting from 3.2 GBq, 0.11 GBq was isolated at 39 min from EOB; (2) Purification method: 15–50% B Column: C1. Starting from 3.2 GBq, 0.19 GBq was isolated at 38 min from EOB. Analytical HPLC R_t_ = 3.3 min.

#### 3.4.9. [*carbonyl*-^11^C]*N*-Phenylpiperidine-1-carboxamide **11**

Synthesised according to the general procedure (six experiments). (1) Purification method: 35% B Column: C1. Starting from 3.6 GBq, 0.19 GBq was isolated at 40 min from EOB; (2) Purification method: 30% B Column: C1. Starting from 3.1 GBq, 0.094 GBq was isolated at 42 min from EOB; (3) 3 equiv. aniline. Purification method: 20–50% B Column: C1. Starting from 3.6 GBq, 0.051 GBq was isolated at 40 min from EOB; (4) 3 equiv. aniline. Purification method: 20–50%% B Column: C1. Starting from 3.2 GBq, 0.0.058 GBq was isolated at 40 min from EOB; (5) 3 equiv. aniline. Purification method: 20–50% B Column: C1. Starting from 4.3 GBq, 0.16 GBq was isolated at 42 min from EOB; (6) 10 equiv. of 1-butanol. Purification method: 20–50% B Column: C1. Starting from 2.7 GBq, 0.026 GBq was isolated at 62 min from EOB. Analytical HPLC R_t_ = 4.7 min.

#### 3.4.10. [*carbonyl*-^11^C]*N*-(4-Methoxyphenyl)piperidine-1-carboxamide **12**

Synthesised according to the general procedure (three experiments). (1) Purification method: 20–50% B Column: C1. Starting from 3.2 GBq, 0.084 GBq was isolated at 41 min from EOB; (2) Purification method: 20–50% B Column: C1. Starting from 1.8 GBq, 0.052 GBq was isolated at 43 min from EOB; (3) 10 equiv. 1-butanol. Purification method: 20–50% B Column: C3. Starting from 2.9 GBq, 0.16 GBq was isolated at 43 min from EOB. Analytical HPLC R_t_ = 4.5 min.

#### 3.4.11. [*carbonyl*-^11^C]*N*-(4-Fluorophenyl)piperidine-1-carboxamide **13**

Synthesised according to the general procedure (four experiments). (1) Purification method: 35% B Column: C1. Starting from 3.7 GBq, 0.072 GBq was isolated at 46 min from EOB; (2) Purification method: 35% B Column: C1. Starting from 3.6 GBq, 0.086 GBq was isolated at 37 min from EOB; (3) Purification method: 35% B Column: C1. Starting from 21.7 GBq, 0.61 GBq was isolated at 37 min from EOB; (4) 10 equiv. 1-butanol. Purification method: 35% B Column: C3. Starting from 4.3 GBq, 0.38 GBq was isolated at 40 min from EOB. Analytical HPLC R_t_ = 4.8 min.

#### 3.4.12. [*carbonyl*-^11^C]*N*-(4-Nitrophenyl)piperidine-1-carboxamide **14**

Synthesised according to the general procedure (three experiments). (1) Purification method: 40% B Column: C1. Starting from 3.3 GBq, 0.028 GBq was isolated at 49 min from EOB; (2) Purification method: 40% B Column: C1. Starting from 3.6 GBq, 0.059 GBq was isolated at 39 min from EOB; (3) 10 equiv. 1-butanol. Purification method: 40% B Column: C3. Starting from 2.7 GBq, 0.007 GBq was isolated at 96 min from EOB. Analytical HPLC R_t_ = 5.4 min.

#### 3.4.13. [*carbonyl*-^11^C]3,4-Dihydroquinazolin-2(1H)-one **15**

Synthesised according to the general procedure (three experiments). (1) Purification method: 20% B Column: C1. Starting from 3.4 GBq, 0.007 GBq was isolated at 43 min from EOB; (2) Purification method: 20% B Column: C1. Starting from 3.1 GBq, 0.018 GBq was isolated at 34 min from EOB. Analytical HPLC R_t_ = 2.7 min.

#### 3.4.14. [*carbonyl*-^11^C]*N*-(2,4-Dichlorobenzyl)-4-phenoxypiperidine-1-carboxamide **19**

Synthesised according to the general procedure (three experiments). (1) Purification method: 60% B Column: C1. Starting from 3.2 GBq, 0.46 GBq was isolated at 33 min from EOB; (2) Purification method: 60% B Column: C2. Starting from 17.8 GBq, 1.9 GBq was isolated at 43 min from EOB; (3) Purification method: 60% B Column: C3. Starting from 12.8 GBq, 2.1 GBq was isolated at 41 min from EOB. Analytical HPLC R_t_ = 7.4 min.

### 3.5. Computational Details

The density functional theory calculations were performed in Jaguar version 9.5, release 11, Schrodinger, Inc., New York, NY, USA, 2016 [74]. To facilitate the calculations, *N*-methyl amine was used as a minimal primary amine. The geometries were optimised using the LACVP** basis set [75] and the B3LYP-D3 a posteriori-corrected functional [76]. Vibrational analyses were performed to characterise the stationary points identified and to calculate zero point energies. The contributions to the free energies were calculated at a temperature of 393.15 K (120 °C) using the B3LYP-D3/LACVP** geometries. For the chloride anion, an entropy of 40.97 cal K^−1^·mol^−1^ was used [77]. Solvation energies were calculated using B3LYP-D3/LACVP**+ and the standard Poisson-Boltzmann continuum solvation model [78] with parameters suitable for THF using the B3LYP-D3/LACVP** geometries.

## 4. Conclusions

An important aspect of PET tracer development is the possibility to label aspiring tracers, to enable their preclinical and subsequent clinical evaluation. The labelling method should give the labelled compound with high enough radioactivity and purity to enable the study. The palladium(II)-mediated oxidative carbonylation of aliphatic and aromatic amines presented herein is a facile and simple method for the synthesis of ^11^C-labelled ureas. In total, 14 symmetrical and unsymmetrical ^11^C-labelled ureas were synthesised and isolated using only [^11^C]CO, a Pd-source and amines as reaction components. DFT-calculations and selectivity experiments supported a reaction proceeding via a ^11^C-labelled isocyanate. The reaction outcome was largely dependent on the amine nucleophilicity, with aliphatic amines performing better than aromatic amines as substrates. However, with the addition of 1-butanol to the reaction mixture, the radiochemical yields of two aromatic ^11^C-labelled ureas were improved. Not all aniline derivatives benefitted from the alcoholic additive, but the addition presents a viable approach when using challenging aromatic amines in ^11^C-urea synthesis. Finally, the advantage of using [^11^C]CO to achieve high molar activity, as opposed to other ^11^C-labelling synthons, was apparent as the isolation of sEH inhibitor **19** in a good 41% radiochemical yield and with high molar activity (247 GBq/μmol–319 Gbq/μmol).

## Figures and Tables

**Table 1:**
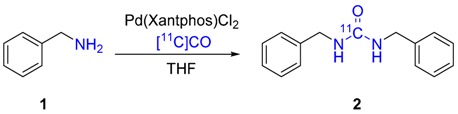
Optimisation of reaction conditions for synthesis of symmetrical ^11^C-labelled urea.

Entry	T (°C)	Time (min)	Conversion ^a^ (%)	Product Selectivity ^b^ (%)	RCY ^c^ (%)
**1**	120	5	56 ± 2.2	>99	55 ± 2.1 (3)
**2**	120	**10**	83 ± 3.9	>99	82 ± 3.9 (3)
**3**	**150**	5	66 ± 4.5	96 ± 2.6	63 ± 4.3 (3)
**4**	**150**	**10**	90 ± 2.5	97 ± 2.1	87 ± 3.4 (3)
**5 ^d^**	120	10	66 ± 1.0	>99	65 ± 1.0 (2)

Conditions: **1** (30 μmol), Pd(Xantphos)Cl_2_ (4 μmol), THF (400 μL). ^a^ Percentage of [^11^C]CO converted to non-volatile products. Decay-corrected. ^b^ Percentage of product formed, assessed by analytical HPLC of crude reaction mixture, after volatiles were purged. ^c^ Radiochemical yield, calculated from the conversion and product selectivity. Number of experiments in brackets. ^d^ 10 μmol of **1**.

**Table 2:**
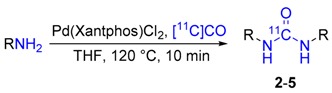
Scope for symmetrical ^11^C-labelled ureas.

Compound	^11^C-Labelled Urea	Conversion ^a^ (%)	RCY ^b^ (%)	RCP ^c^ (%)
**2**	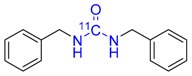	81 ± 5	65 ± 1	>99
			41 ^d^	
**3**	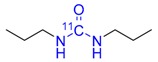	67 ± 4	40 ± 6	>99
**4**	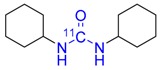	71 ± 2	48 ± 4	>99
**5**	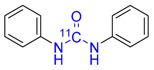	15 ± 1	4 ± 1	>99

Conditions: Amine (30 μmol), Pd(Xantphos)Cl_2_ (4 μmol), THF (400 μL). All experiments were performed in duplicate. ^a^ Percentage of [^11^C]CO converted to non-volatile products. Decay-corrected. ^b^ Radiochemical yield. Based on the ^11^C-labelled product obtained after semi-preparative HPLC and amount of [^11^C]CO collected in the reaction vial. Decay-corrected. ^c^ Radiochemical purity. Determined by analytical HPLC of the isolated ^11^C-labelled product. ^d^ 5 min reaction time, single experiment.

**Table 3:**
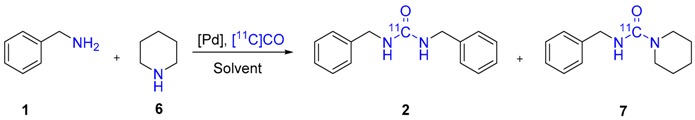
Optimisation of reaction conditions for synthesis of unsymmetrical ^11^C-labelled urea.

Entry	Catalyst	T (°C)	6 (Equiv.)	Conversion ^a^ (%)	Product Selectivity ^b^ (%)	2:7 ^c^	RCY ^d^ (%)
**1**	Pd(Xantphos)Cl_2_	120	1	53 ± 5.6	79 ± 2.9	12:88	42 ± 5.9 (3)
**2**	**Pd(PPh_3_)_2_Cl_2_**	120	1	69 ± 4.1	46 ± 3.6	16:84	32 ± 3.2 (3)
**3**	**Pd(OAc)_2_ + dppf**	120	1	95 ± 3.5	13 ± 3.5	11:89	12 ± 4.0 (2)
**4**	**Pd(OAc)_2_ + dppp**	120	1	75 ± 2.5	21 ± 0.5	23:77	16 ± 0.5 (2)
**5**	**Pd(OAc)_2_ + Xantphos**	120	1	67 ± 9	10 ± 1	23:77	7 ± 1.5 (2)
**6 ^e^**	Pd(Xantphos)Cl_2_	120	1	43 ± 1.7	49 ± 3.6	16:84	21 ± 1.6 (3)
**7**	Pd(Xantphos)Cl_2_	**80**	1	57 ± 9.2	44 ± 6.8	9:91	26 ± 8.5 (3)
**8**	Pd(Xantphos)Cl_2_	**150**	1	44 ± 11	87 ± 4.3	9:91	41 ± 6.2 (4)
**9**	Pd(Xantphos)Cl_2_	120	**2**	46 ± 4.3	63 ± 2.2	9:91	29 ± 3.3 (3)
**10**	Pd(Xantphos)Cl_2_	120	**5**	58 ± 1.7	71 ± 3.6	2:98	42 ± 3.1 (3)
**11 ^f^**	Pd(Xantphos)Cl_2_	120	1	67 ± 1.7	89 ± 3.3	7:93	60 ± 3.4 (3)
**12 ^g^**	Pd(PPh_3_)_4_	120	1	93	-	-	-

Conditions: **1** (30 μmol), **2** (30 μmol), catalyst ([Pd] 4 μmol + ligand 4 μmol), THF (400 μL). 5 min reaction time unless otherwise stated. ^a^ Percentage of [^11^C]CO converted to non-volatile products, after purge. Decay-corrected. ^b^ Percentage of product formed, assessed by analytical HPLC of crude reaction mixture, after purge. ^c^ Product ratio of **2** to **7**, assessed by analytical HPLC of crude reaction mixture. ^d^ Radiochemical yield, calculated from the conversion and product selectivity. Number of experiments in brackets. ^e^ DMF as solvent. ^f^ 10 min reaction time. ^g^ Single experiment.

**Table 4:**
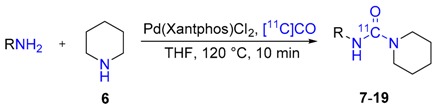
Scope for unsymmetrical ^11^C-labelled ureas.

Compound	^11^C-Labelled Urea	Conversion ^a^ (%)	RCY ^b^ (%)	RCP ^c^ (%)
**7**	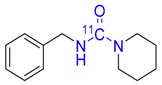	65 ± 0	41 ± 6	98 ± 1
		39 ^d^	17 ^d^	
		59 ^e^	31 ^e^	
**8**	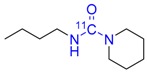	55 ± 4	23 ± 1	>99
		60 ^e^	14 ^e^	
**9**	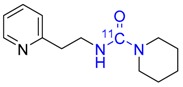	20 ± 0	12 ± 1	>99
**10**	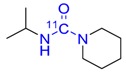	35 ± 6	14 ± 4	>99
**11**	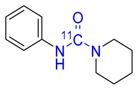	60 ± 3 ^f^	7 ± 2 ^f^	80 ± 7 ^f^
		59 ± 1	12 ± 0 ^g^	97 ± 2 ^g^
		70 ^e^	6 ^e^	88 ^e^
**12**	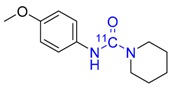	66 ± 3	9 ± 1	99 ± 1
		74 ^e^	21 ^e^	
**13**	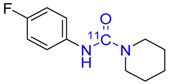	67 ± 9 ^f^	8 ± 1 ^f^	>99
		88 ^e^	28 ^e^	
**14**	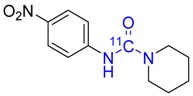	63 ± 0	5 ± 1	>99
		83 ^e^	6 ^e^	
**15**	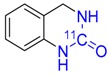	27 ± 1	1 ± 0	90 ± 10
		90 ^e^	Trace ^e^	-
**16**	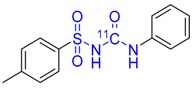	12 ± 2	Trace	-
**17**	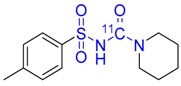	51 ± 0	Trace	-
**18**	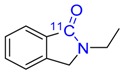	66 ± 8	-	-
**19**	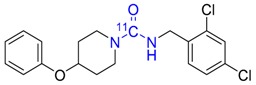	74	41 ± 7 ^f^	99 ± 0 ^f^

Conditions as in entry 11, Table 2. All experiments were performed in duplicate unless otherwise stated. ^a^ Percentage of [^11^C]CO converted to non-volatile products. Decay-corrected. ^b^ Radiochemical yield. Based on the ^11^C-labelled product obtained after semi-preparative HPLC and amount of [^11^C]CO collected in the reaction vial. Decay-corrected. ^c^ Radiochemical purity. Determined by analytical HPLC of the isolated ^11^C-labelled product. ^d^ 5 min reaction time, one experiment. ^e^ 10 equiv. of 1-butanol added, single experiment. ^f^ Average of three experiments. ^g^ 3 equiv. of aniline used.

## Supplementary Materials

The following is available online: Definitions and molar activity calculations, ^1^H and ^13^C-NMR chromatograms of all synthesised reference compounds, HPLC chromatograms of isolated ^11^C-compounds, DFT calculated structures of intermediates on path **A**.
