# Supplementary material for: Synthesis of 11C-Labelled Ureas by Palladium(II)-Mediated Oxidative Carbonylation
Source: Molecules. 2017 Oct 10;22(10):1688. doi: 10.3390/molecules22101688 (PMC6151465; doi:10.3390/molecules22101688)
Supplement: Supplementary file 1 [file molecules-22-01688-s001.pdf]

Supporting information

Synthesis of ^{11}C-labelled ureas by palladium (II)-mediated oxidative carbonylation

Sara Roslin, Peter Brandt, Patrik Nordeman, Mats Larhed, Luke R. Odell and Jonas Eriksson

Table of contents

Calculations and definitions	S2
Molar activity calculations	S3
3D-structures from Scheme 2	S4
NMR spectra – reference compounds	S5
HPLC Chromatograms – ^{11}C-labelled ureas	S21
Reference list	S38

Calculations and definitions

[^{11}C]CO was transferred to the capped reaction vial and the radioactivity was measured to determine the starting amount of [^{11}C]CO (A_1). The reaction was heated during the specified reaction time. When finished, the radioactivity was measured (A_2) before venting the reaction vial and purging with N_2 to remove unreacted [^{11}C]CO and, possibly, volatile ^{11}C-labelled compounds formed during the reaction. A third radioactivity measurement (A_3) was performed before either preparation of a sample for determination of product selectivity or semi-preparative HPLC purification. After isolation and a final radioactivity measurement (A_4) of the ^{11}C-labelled product, an aliquot was analyzed to determine radiochemical purity and the identity of the ^{11}C-labelled product was confirmed using the isotopically unmodified product as reference. Activities were decay corrected to the same time point before used in calculations.

Conversion

The conversion, the measurement of [^{11}C]CO incorporated into non-volatile ^{11}C-labelled compounds, was based on the radioactivity measurements A_3 and A_2.

$$\text{Conversion (\%)} = \frac{\text{A3 (d.c.)}}{A2} \times 100$$

Product selectivity

Percentage of ^{11}C-labelled product formed, based on HPLC analysis of crude reaction mixture.

Radiochemical yield in optimization tables 1 and 3

An estimate of the radiochemical yield (RCY) of the non-isolated ^{11}C-labelled product based on the [^{11}C]CO-conversion and the ^{11}C-labelled product selectivity.

$$RCY\ (\%) = Conversion\ \times Product\ selectivity$$

Radiochemical yield

Based on the activity of the isolated ^{11}C-labelled product (A_4) and the starting amount of [^{11}C]CO, transferred to the reaction vial (A_1).

$$RCY\ (\%) = \frac{A4\ (d.c.)}{A1} \times 100$$

Radiochemical purity

Based on the HPLC analysis of an aliquot from the isolated ^{11}C-labelled product fraction.

Identity of synthesized ^{11}C-labelled compound

The identity of a labelled compound was confirmed by adding isotopically unmodified compound (UV-active) to an aliquot of the isolated ^{11}C-labelled product and comparing the retention times of the UV-peak and radio-peak on analytical HPLC.

Molar activity calculations

A calibration curve for *N*-(2,4-dichlorobenzyl)-4-phenoxypiperidine-1-carboxamide (**19**) was prepared using five concentrations; 0.25, 0.5, 1.0, 2.0 and 5.0 µg/mL. 50 µL was injected, starting from the lowest concentration, and analyzed at 221 nm to construct a calibration curve (Figure S1). A blank sample consisting of acetonitrile was injected between every run to avoid carry-over.
The molar activity for **19** was determined in two experiments and calculated from the activity of the isolated product (A_4) and the volume and concentration of the product fraction (Table S1).

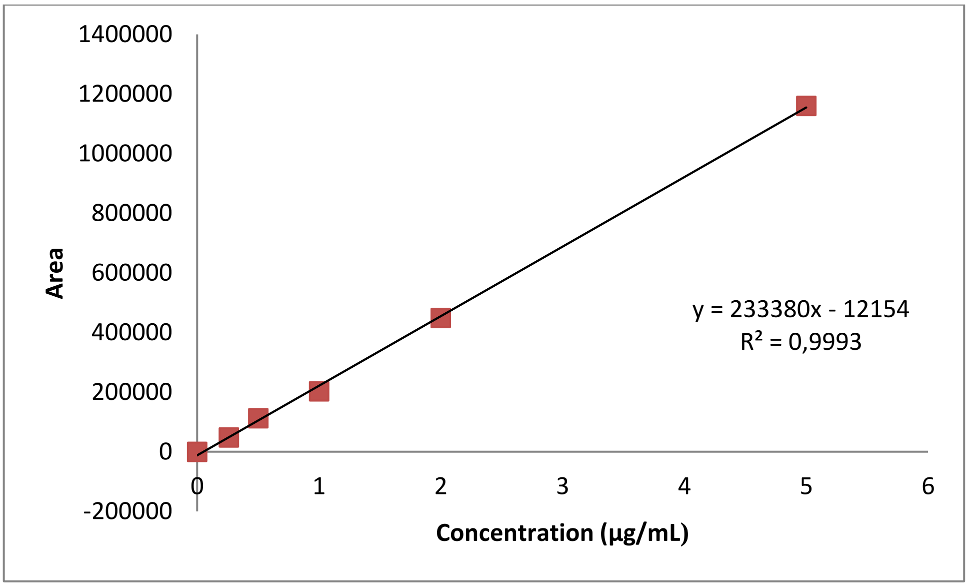

Figure S1. Calibration curve for *N*-(2,4-dichlorobenzyl)-4-phenoxypiperidine-1-carboxamide (**19**).

Table S1. Determination of molar activity.

Experiment	Area	Concentration (µg/mL)	Volume (mL)	Mass (µg)	Amount (µmol)	Activity (GBq)	Molar activity (GBq/µmol)
**1**	114169	0.599	5.12	2.86	0.00754	1.86	247
**2**	31211	0.186	13.5	2.51	0.00662	2.11	319

3D-structures from Scheme 2

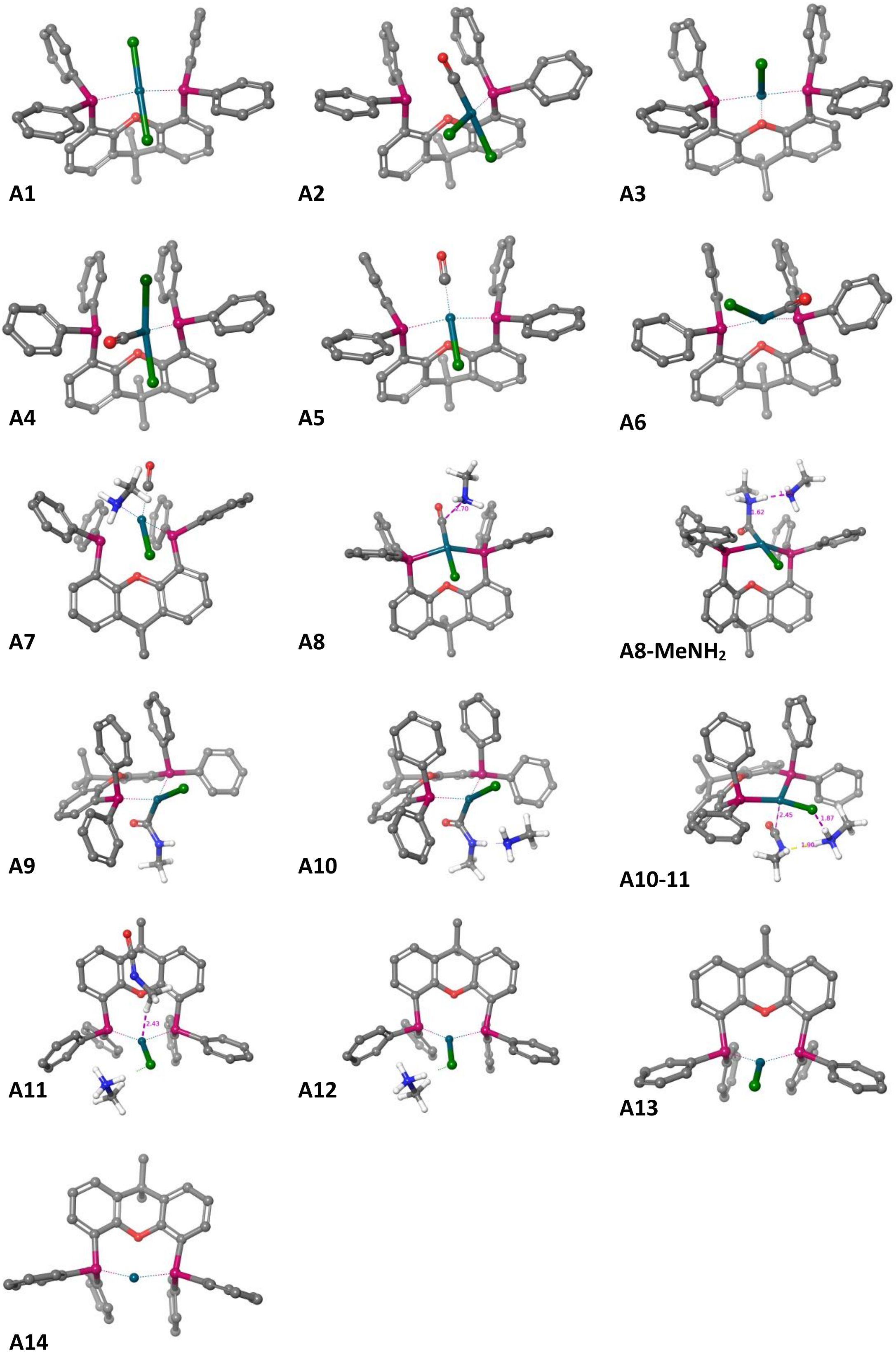

Figure S2. Optimized structures of intermediates and one transition state shown in Scheme 2.

NMR spectra

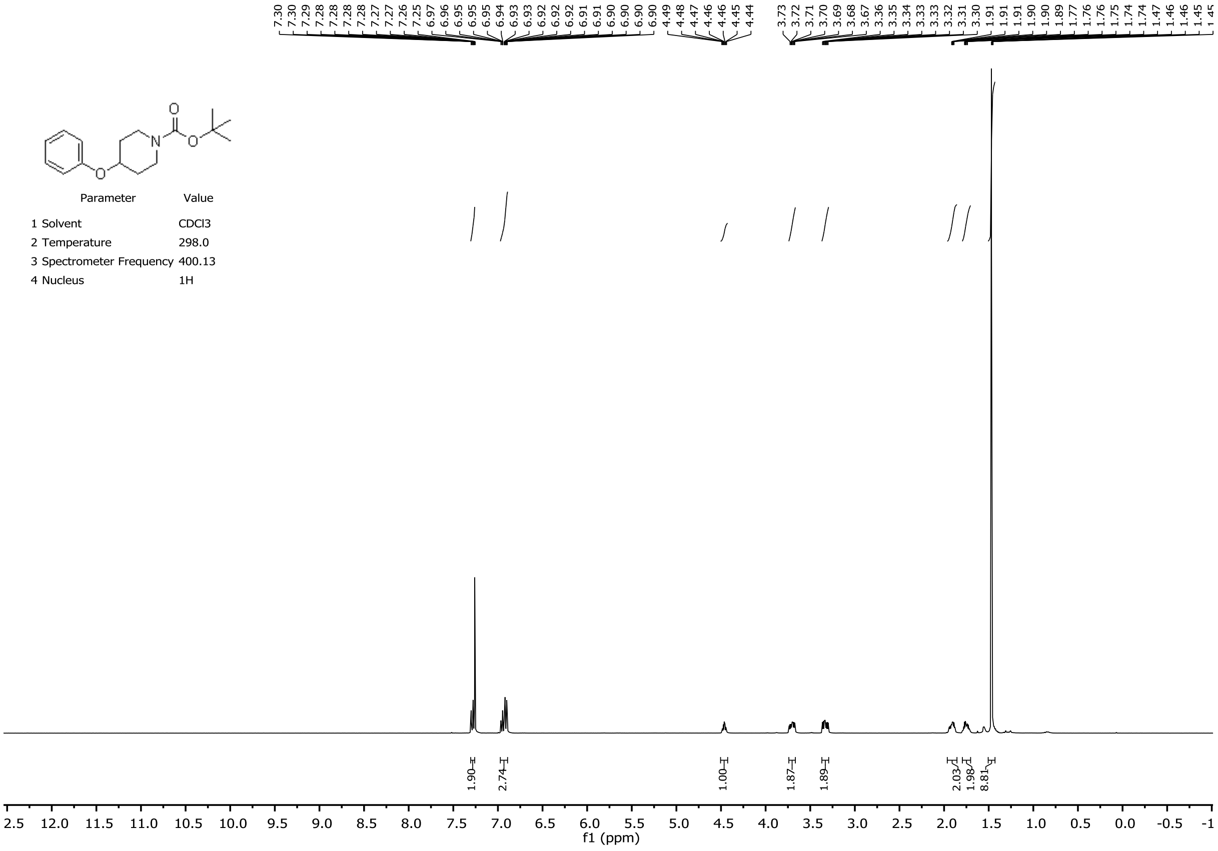
Parameter Value
1 Solvent CDCl3
2 Temperature 298.0
3 Spectrometer Frequency 400.13
4 Nucleus 1H
1.90
2.74
1.00
1.87
1.89
2.03
1.98
8.81
f1 (ppm)

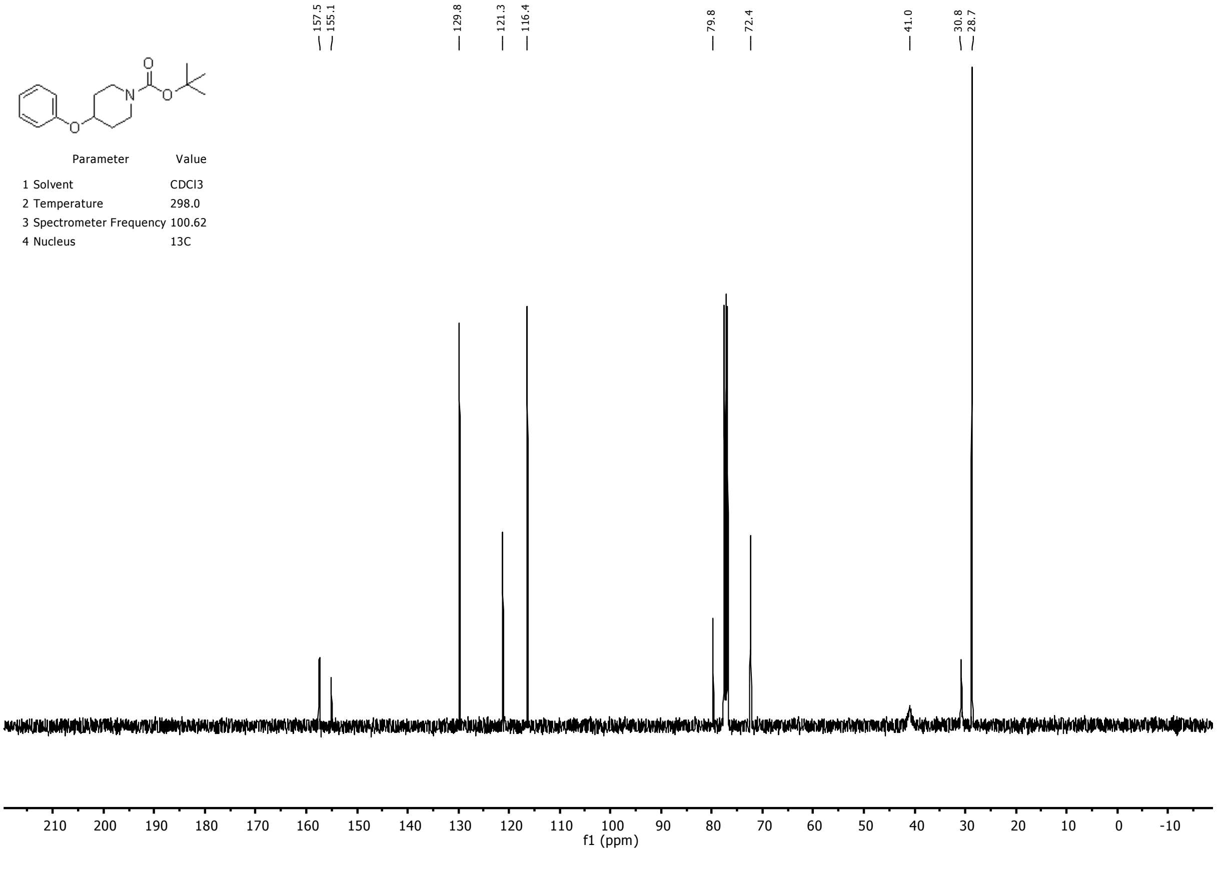
157.5
155.1
129.8
121.3
116.4
79.8
72.4
41.0
30.8
28.7
Parameter Value
1 Solvent CDCl3
2 Temperature 298.0
3 Spectrometer Frequency 100.62
4 Nucleus 13C
f1 (ppm)

tert-Butyl 4-phenoxypiperidine-1-carboxylate [1] CAS: 155989-69-8

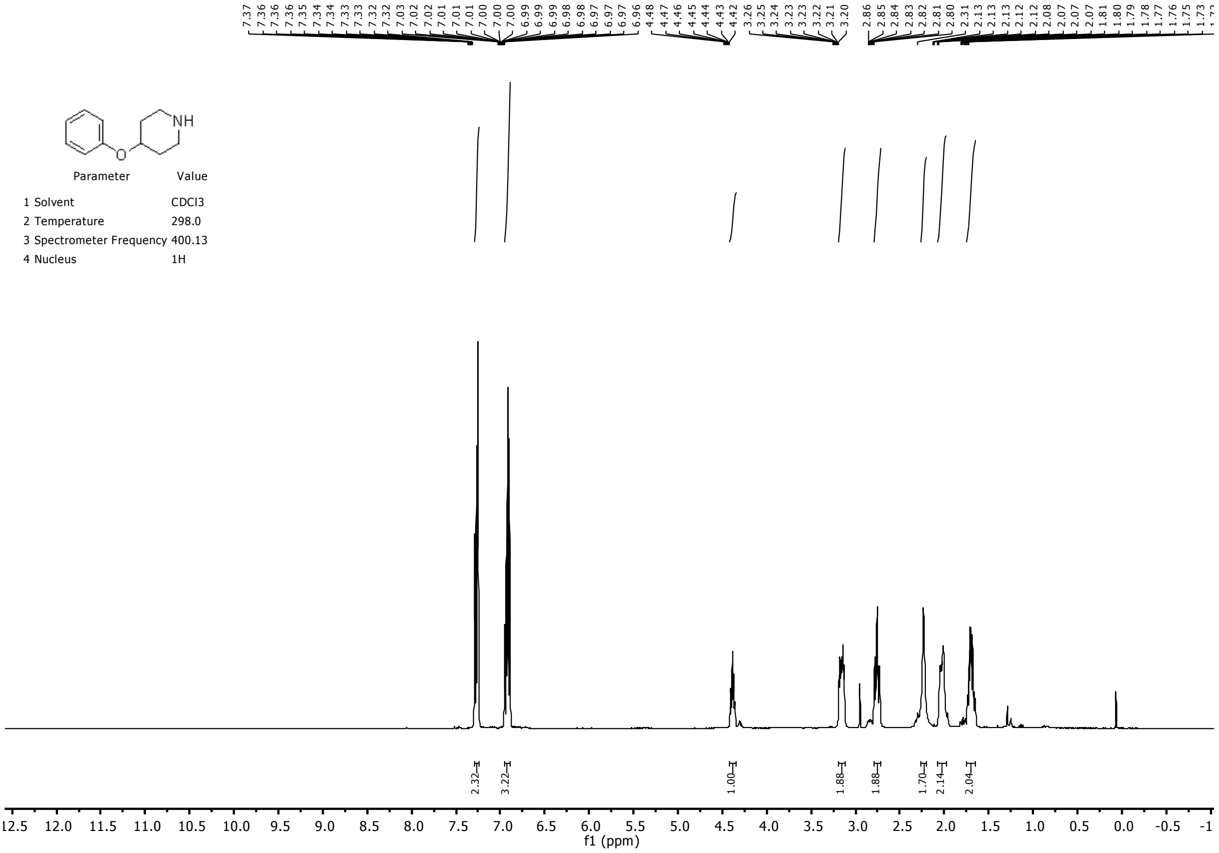

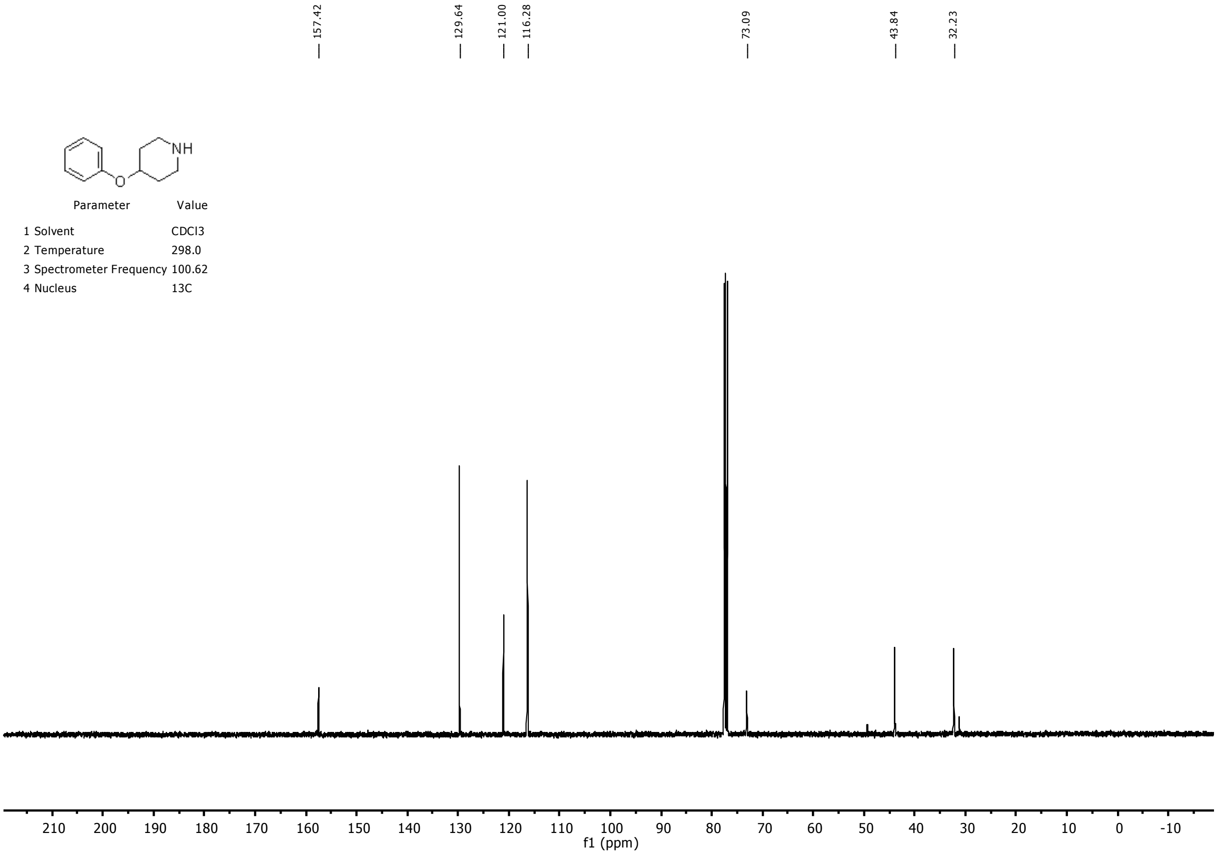

4-Phenoxypiperidine [1] CAS: 3202-33-3

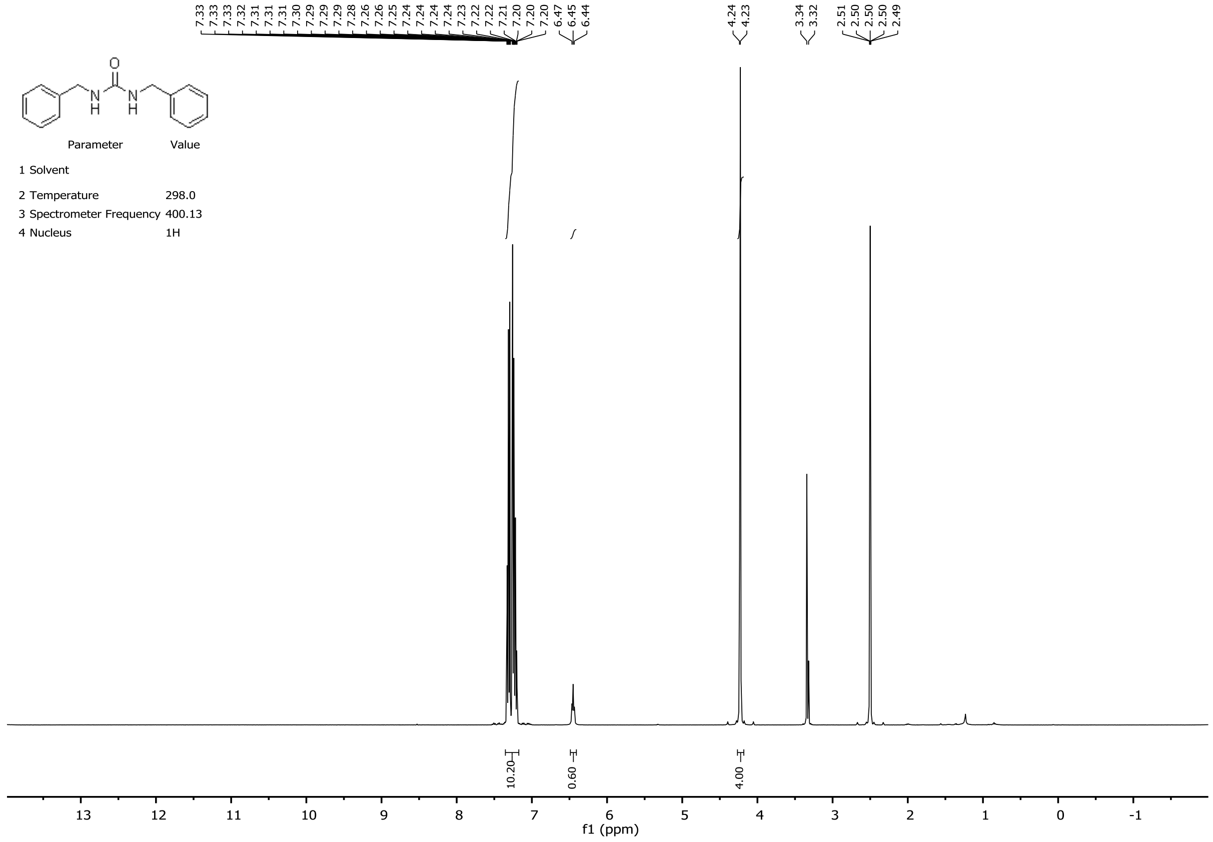

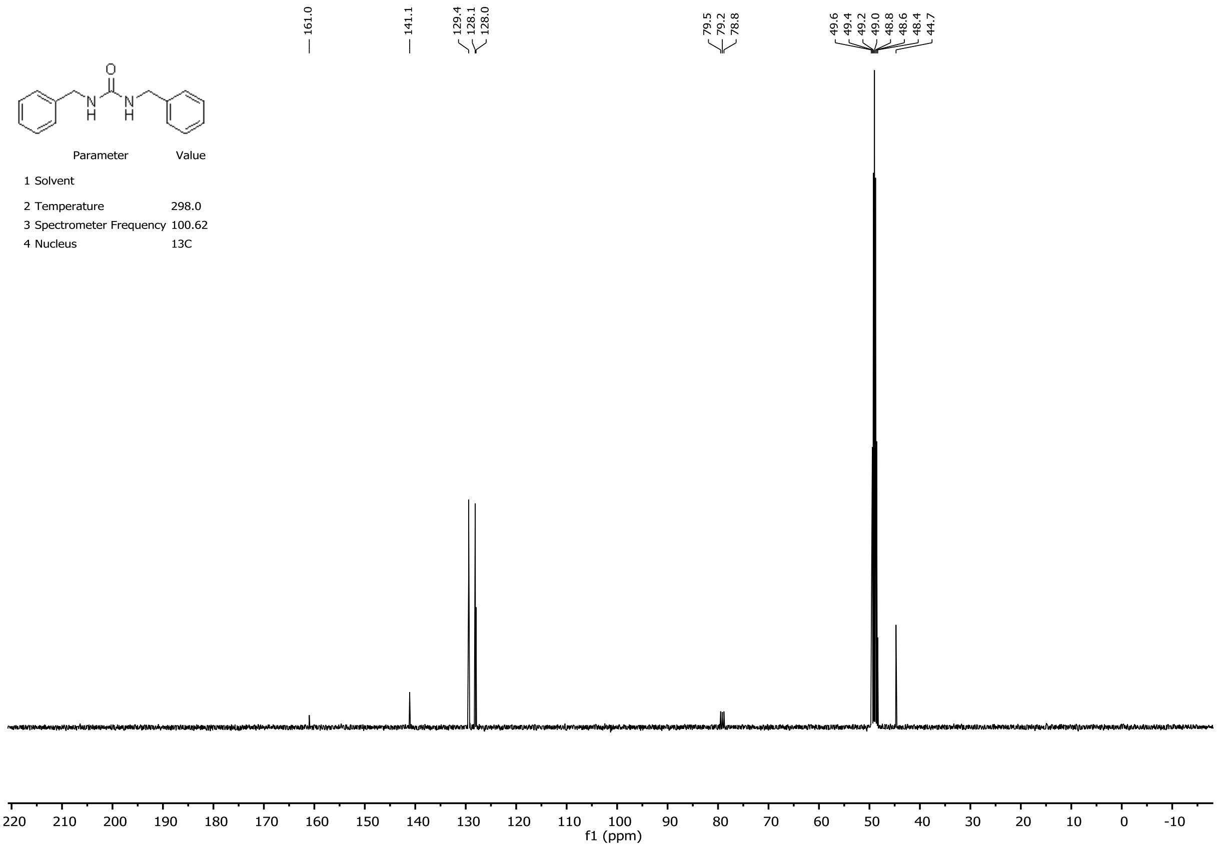

1,3-Dibenzylurea [2] CAS: 1466-67-7

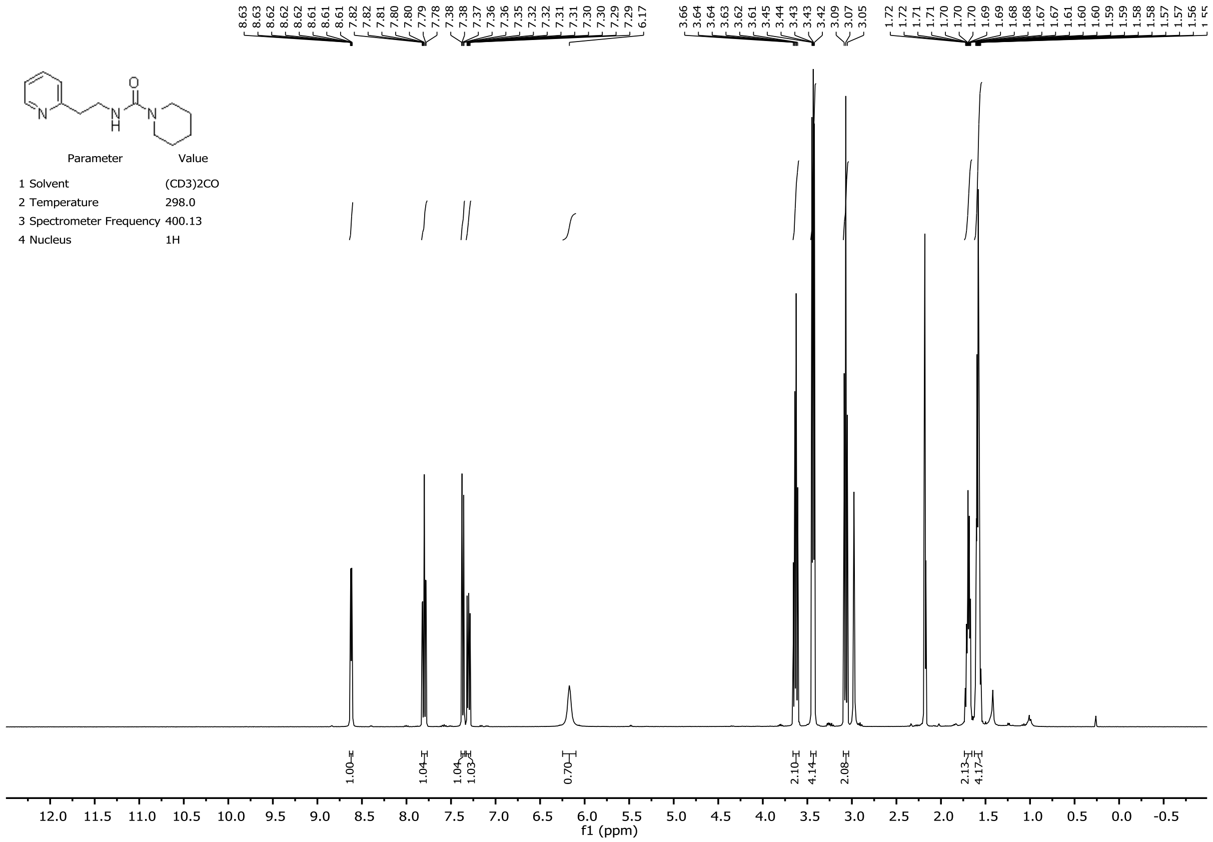

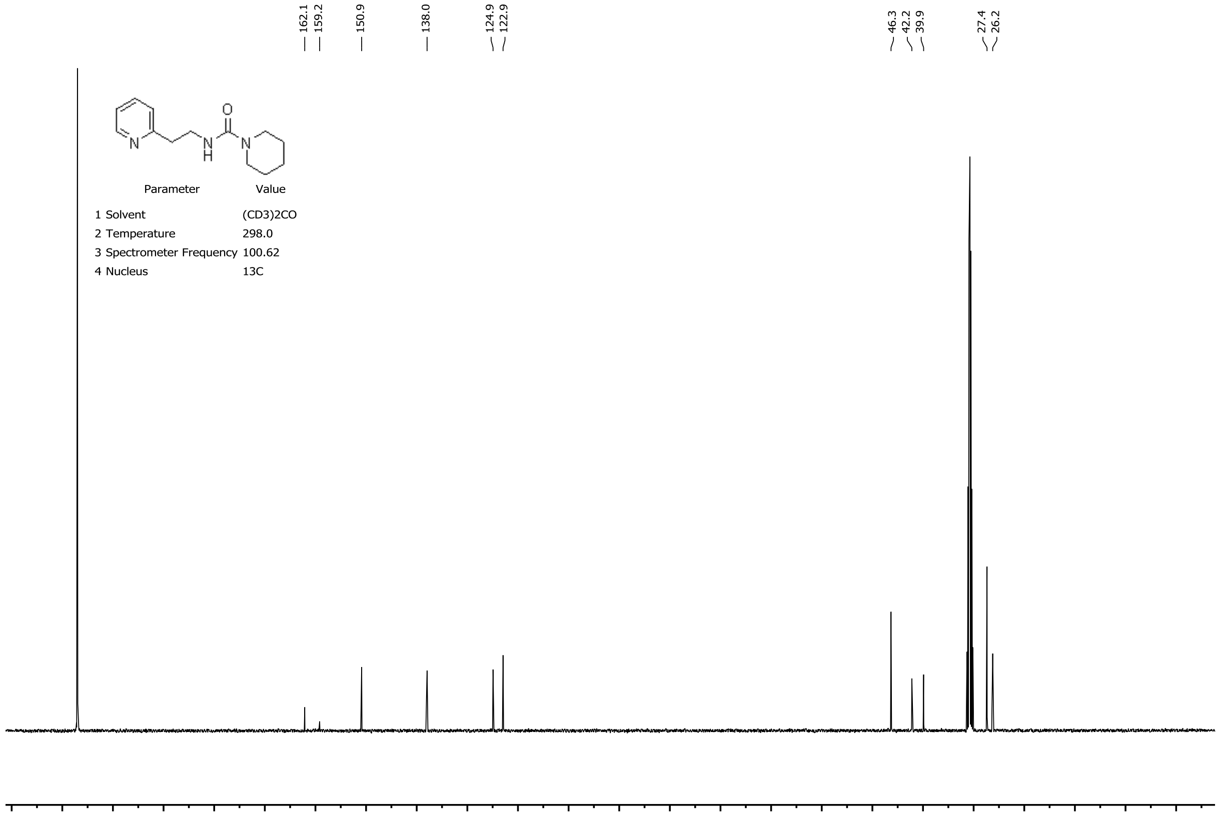

N-(2-(Pyridin-2-yl)ethyl)piperidine-1-carboxamide CAS: 1710806-84-0

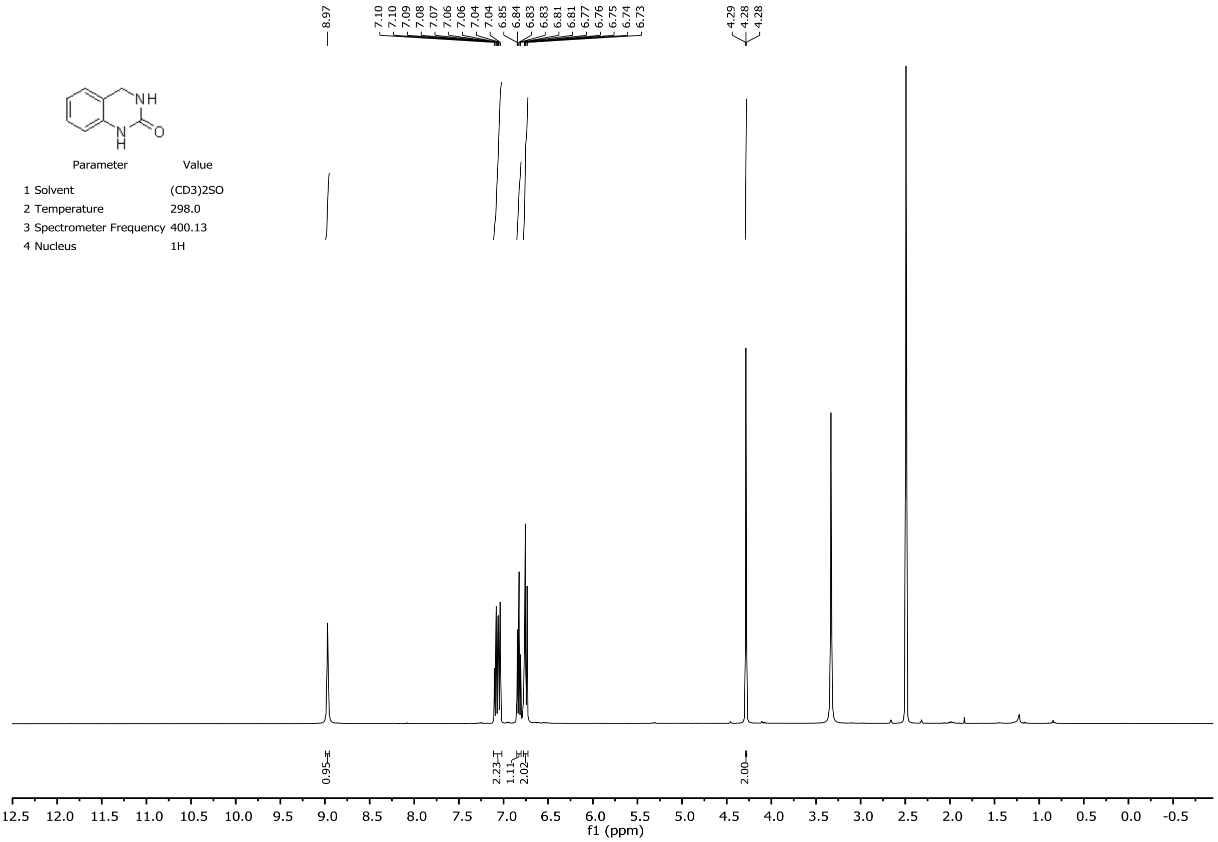

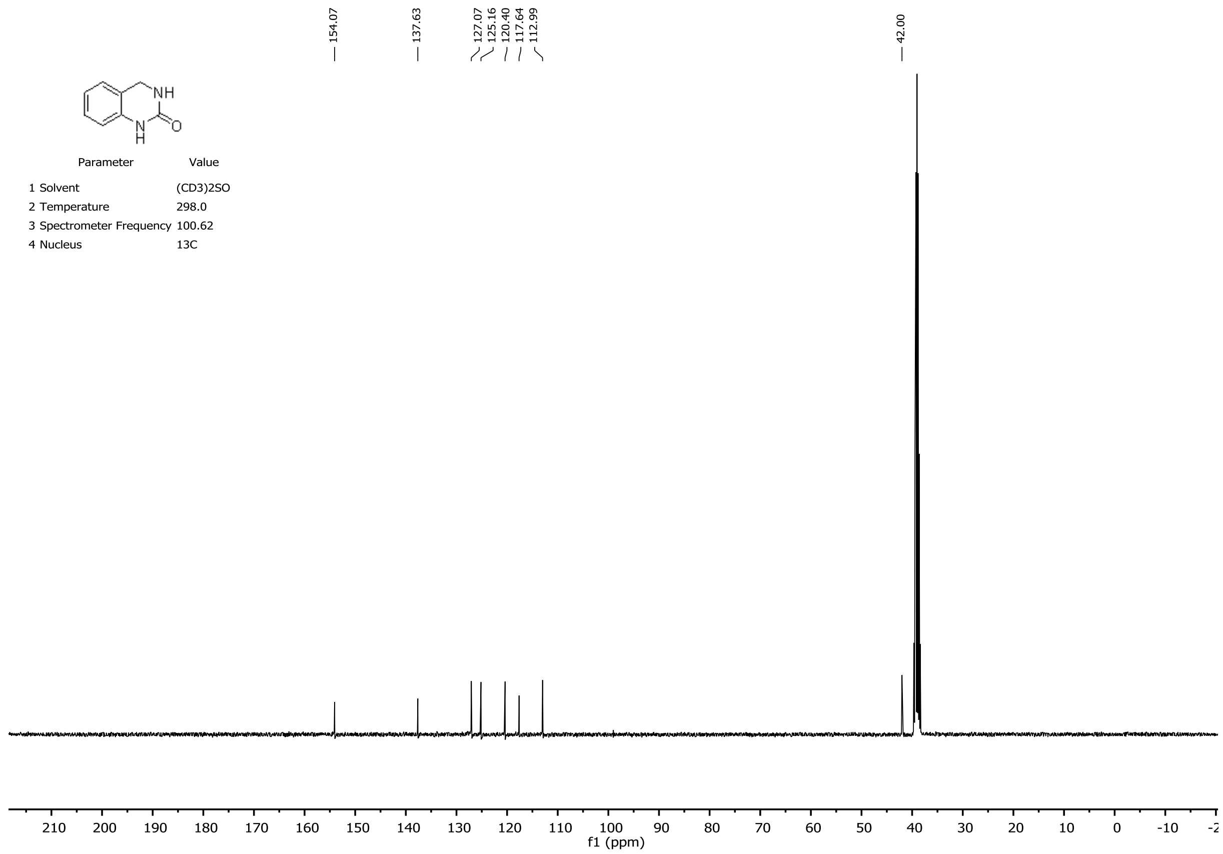

3,4-Dihydroquinazolin-2(1H)-one [3] CAS: 66655-67-2

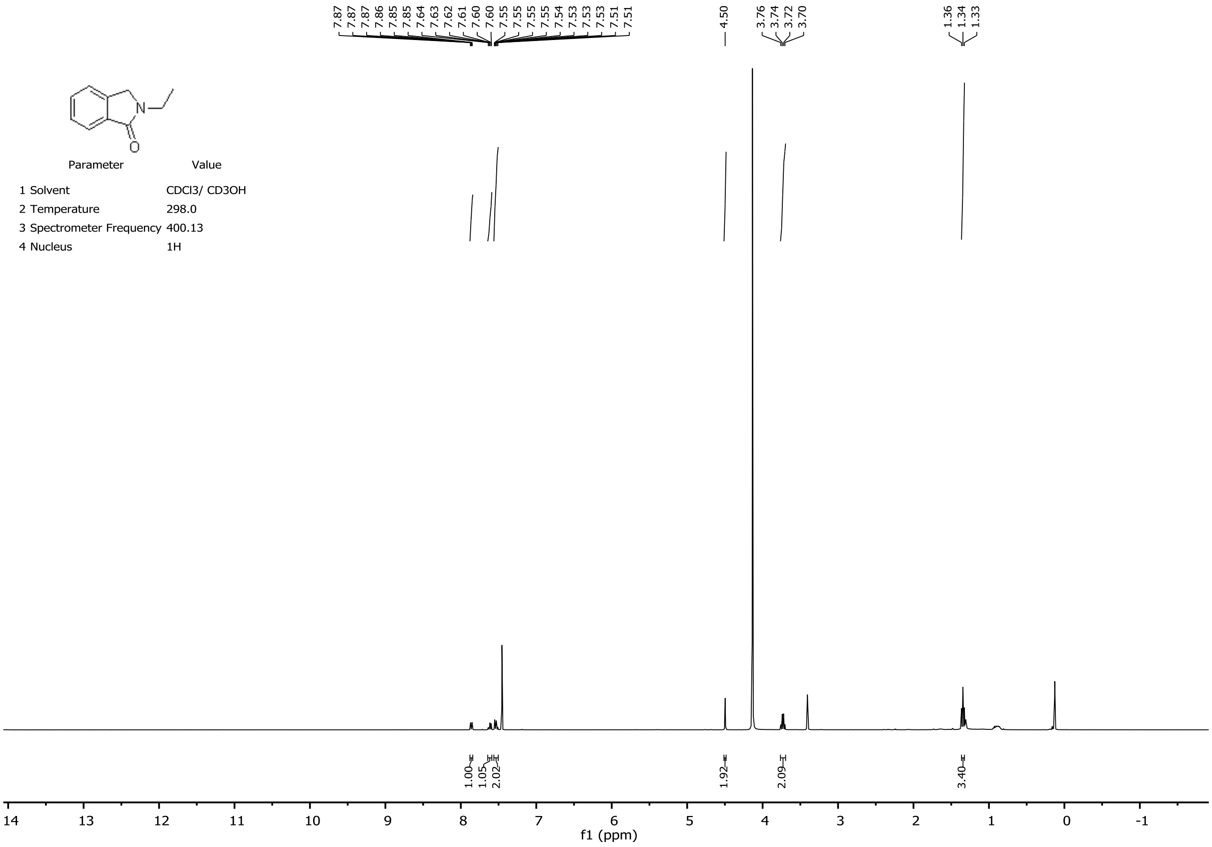

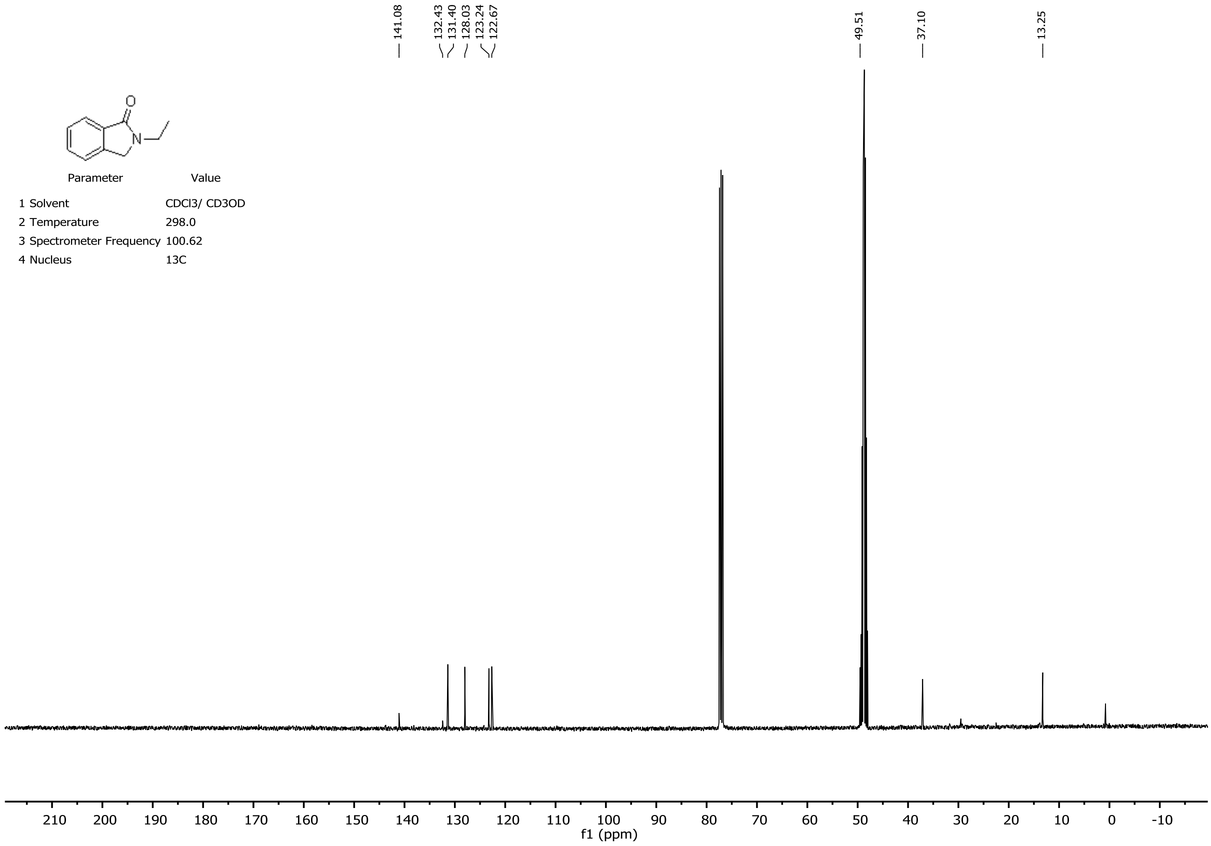

2-Ethylisoindolin-1-one [4] CAS: 23967-95-5

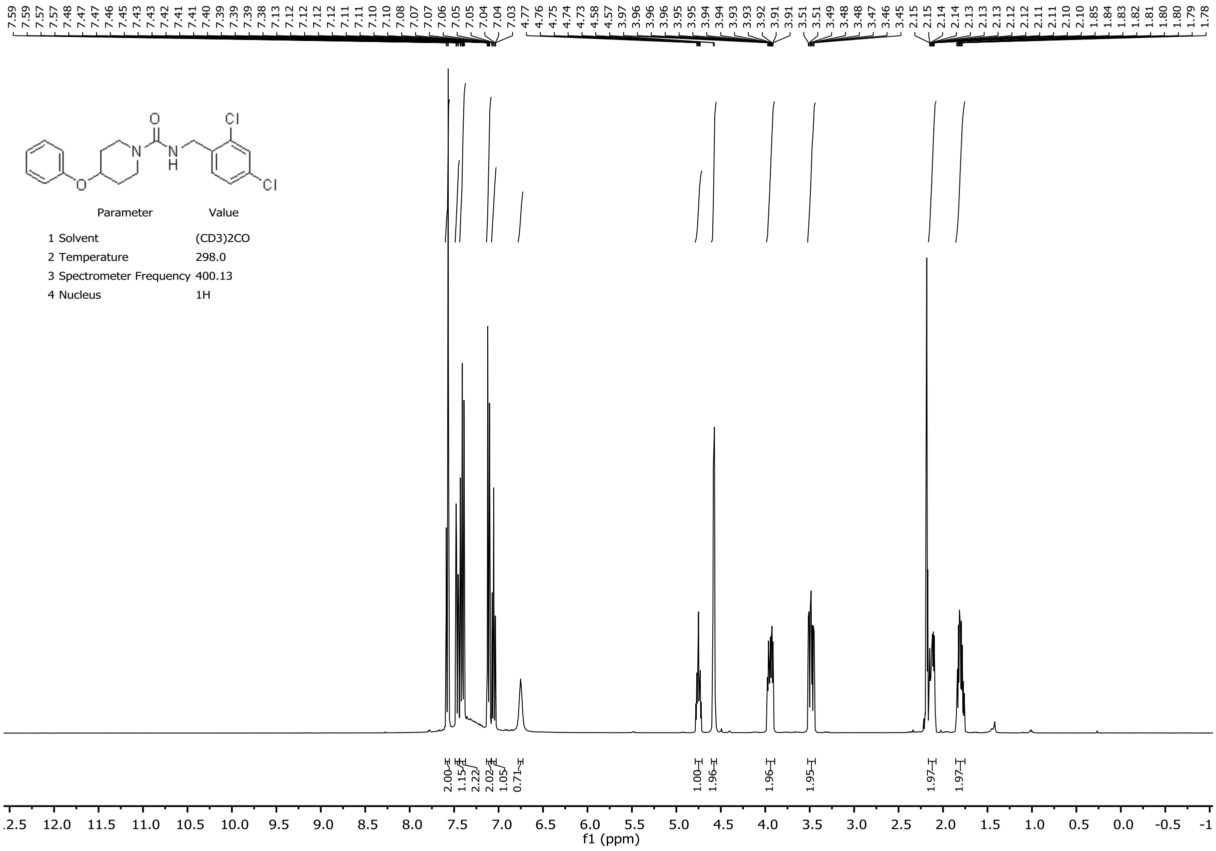

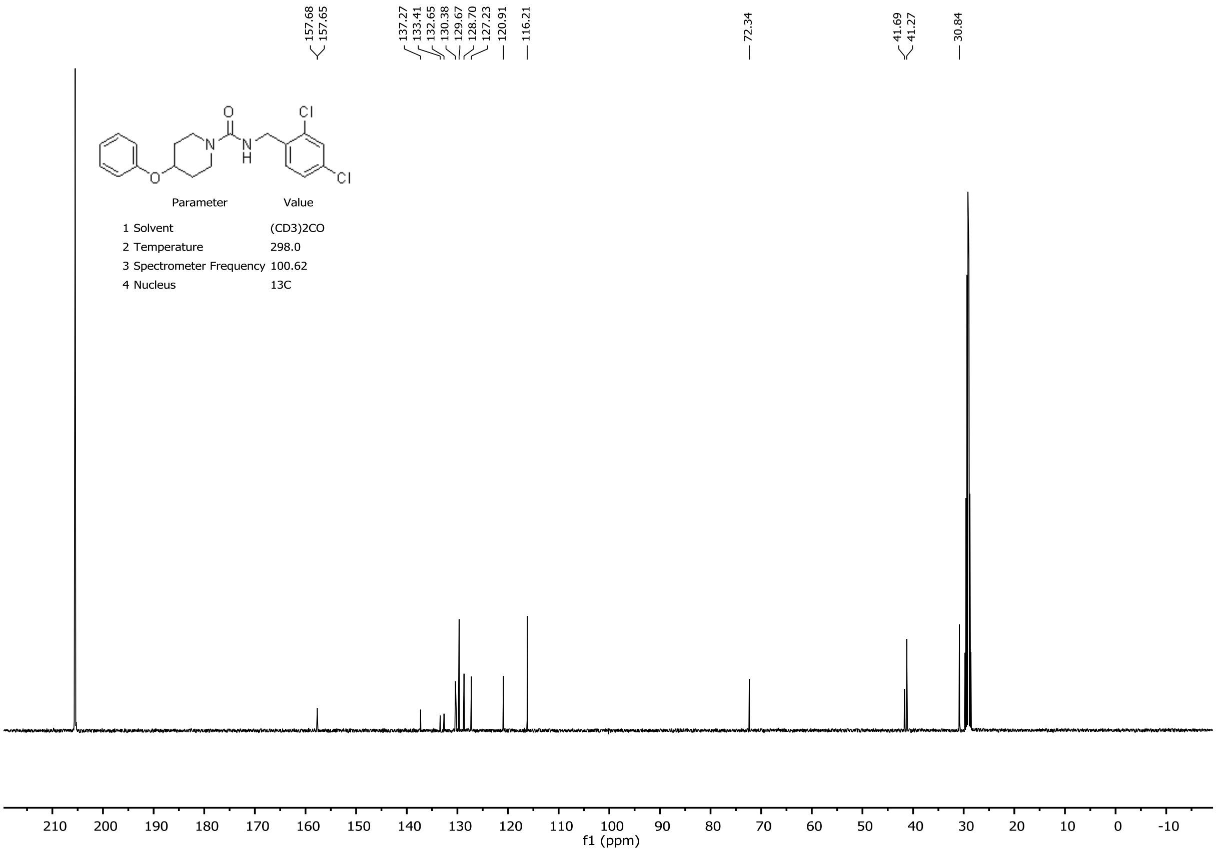

N-(2,4-Dichlorobenzyl)-4-phenoxypiperidine-1-carboxamide CAS: 950645-62-2

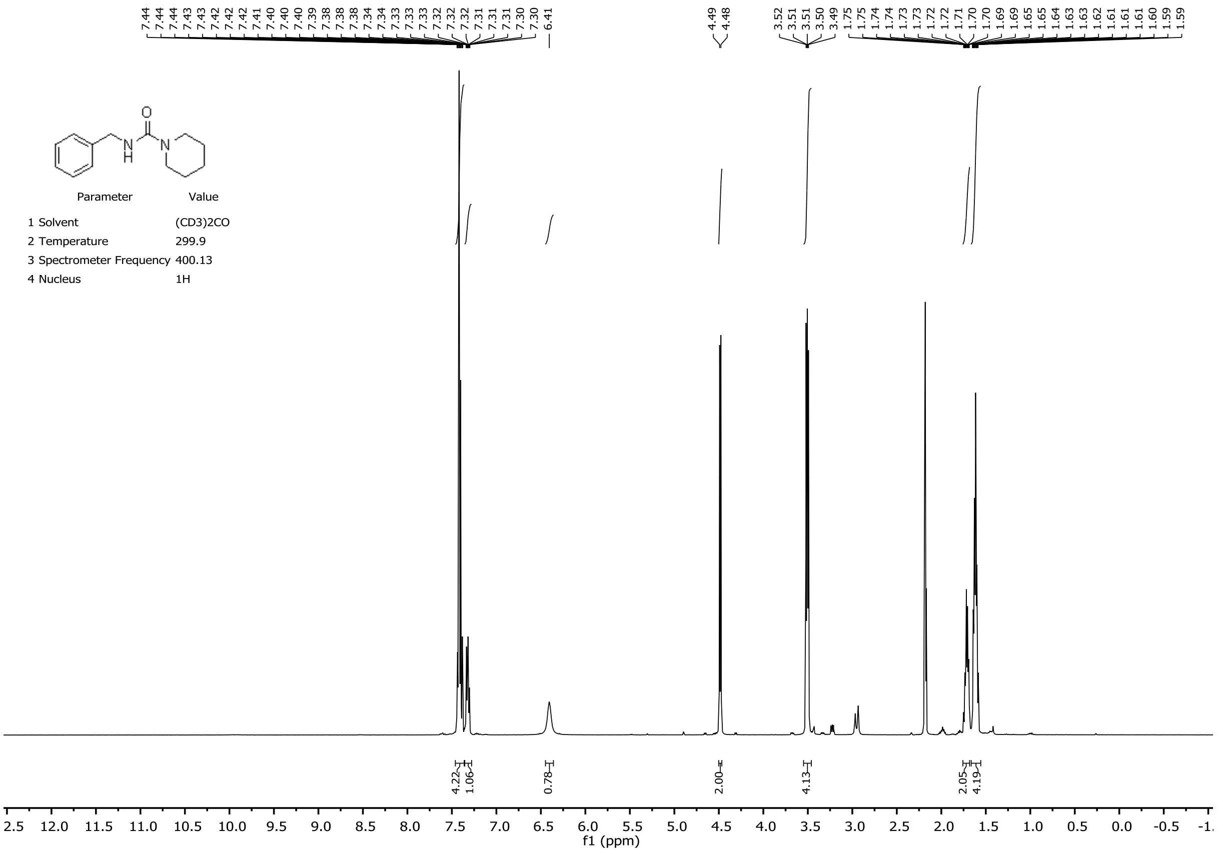

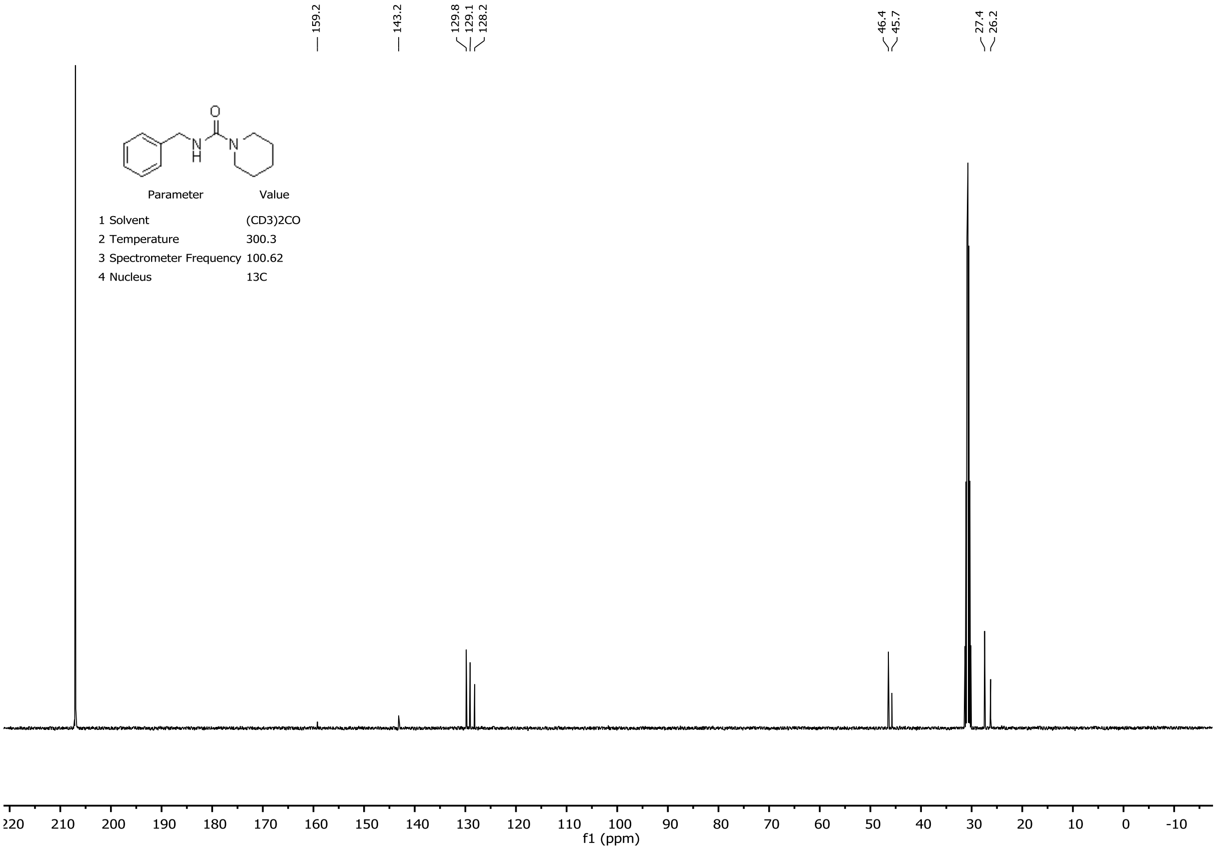

N-Benzylpiperidine-1-carboxamide [5] CAS: 39531-35-6

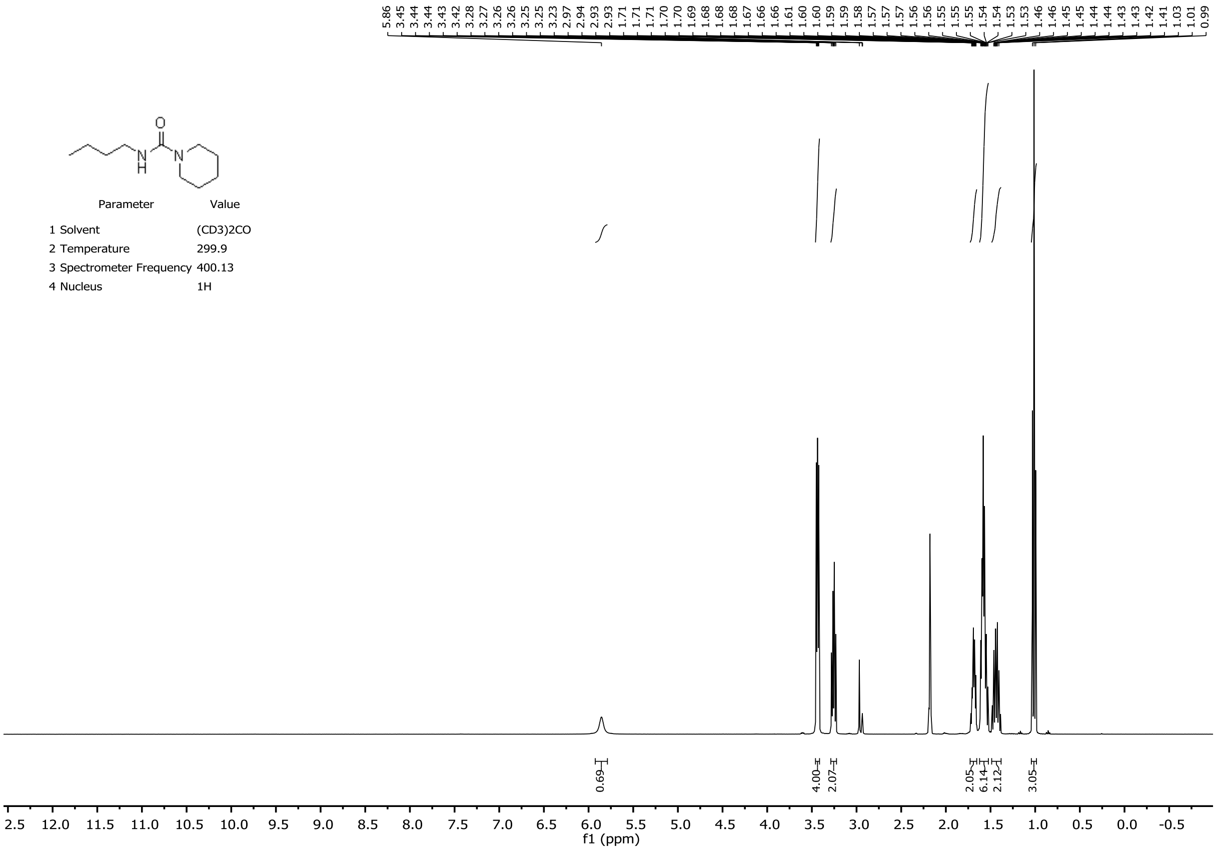

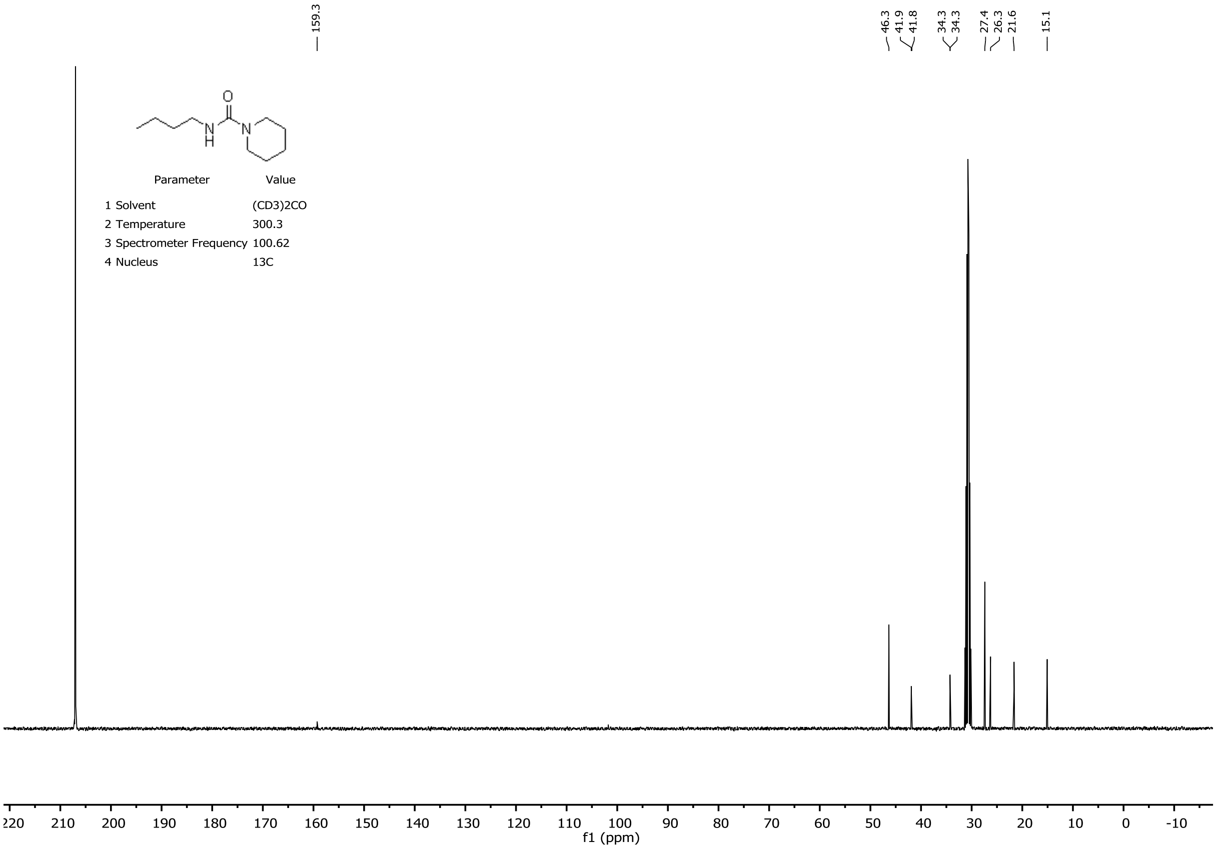

N-Butylpiperidine-1-carboxamide CAS: 1461-79-6

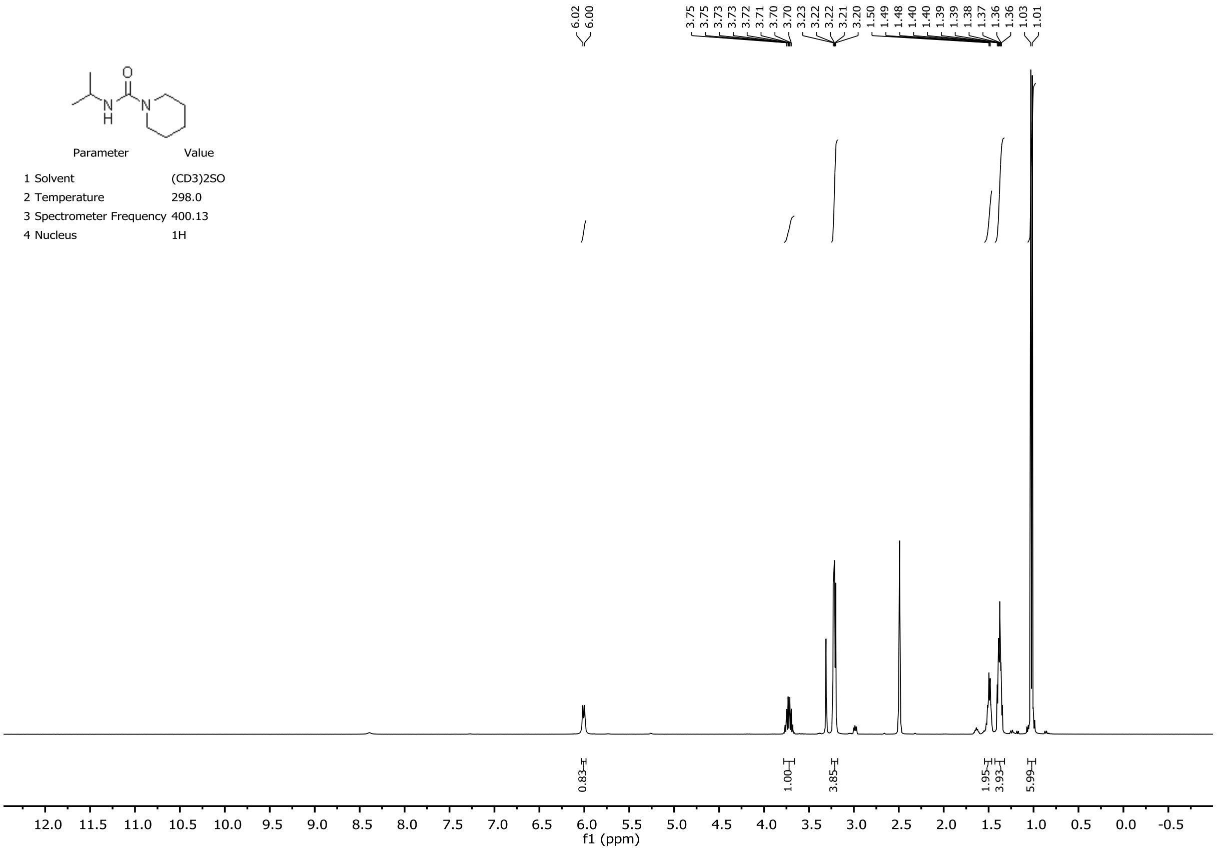

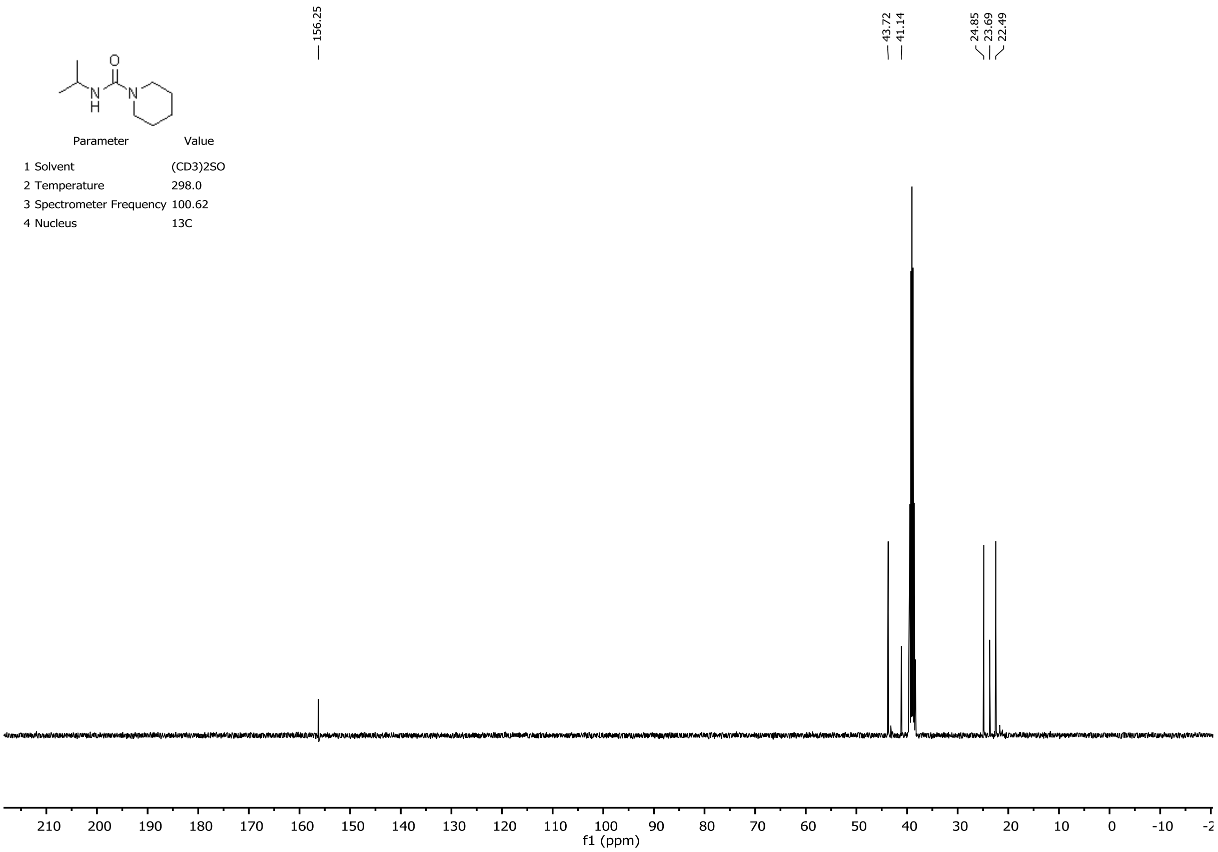

N-Isopropylpiperidine-1-carboxamide CAS: 10581-04-1

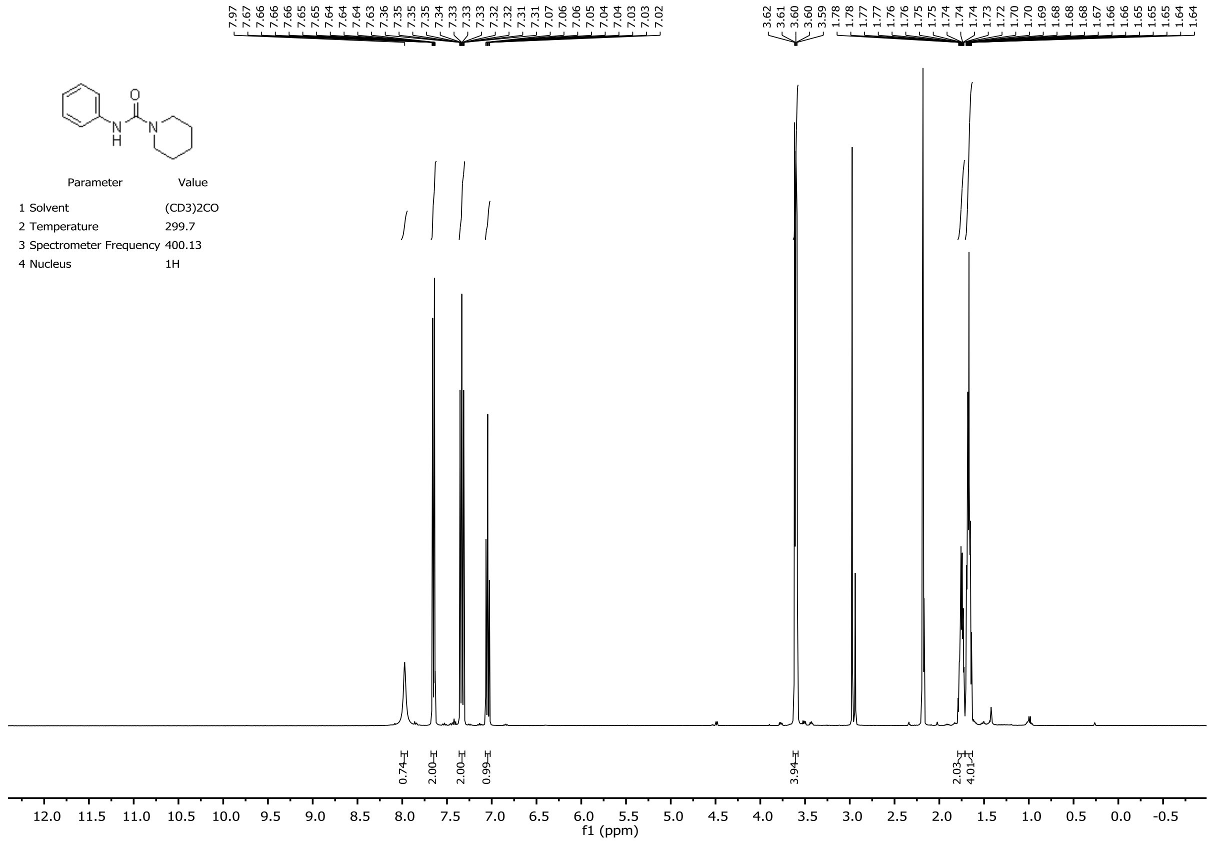

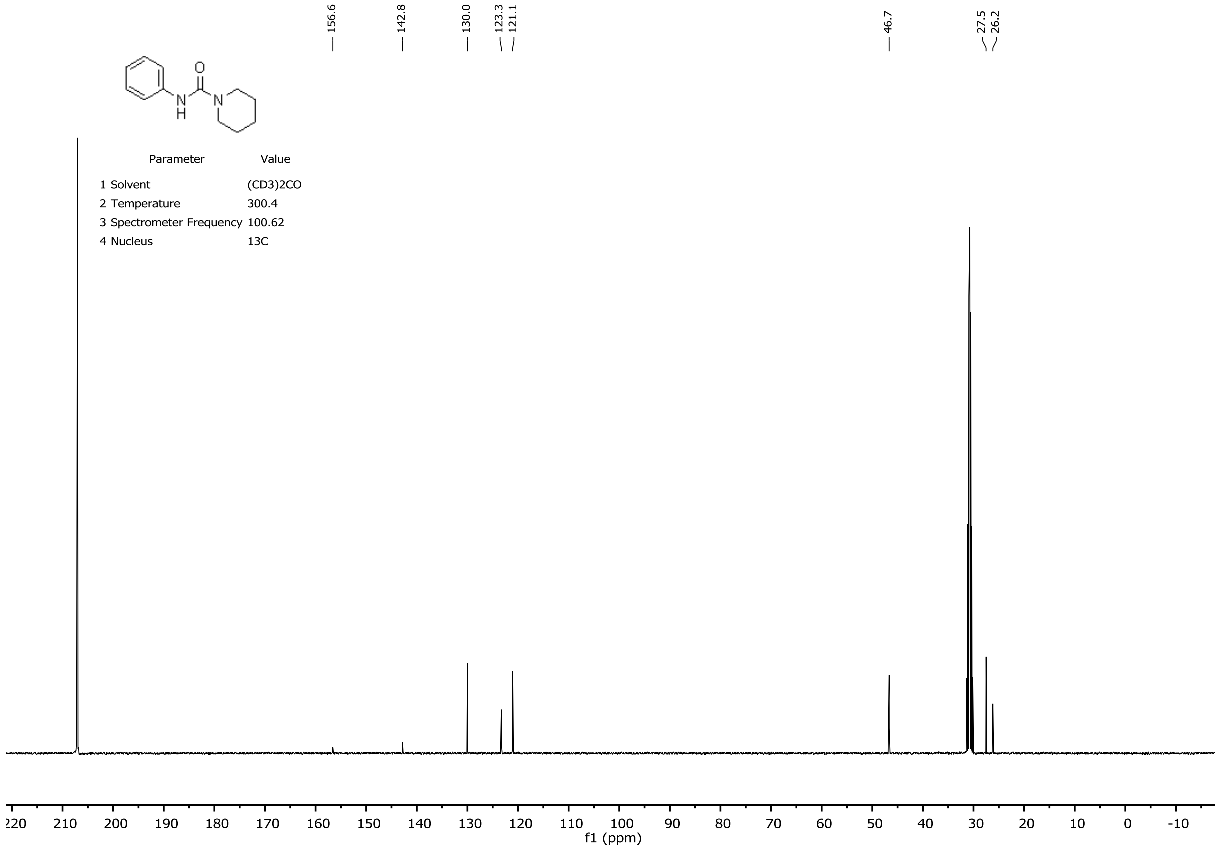

N-Phenylpiperidine-1-carboxamide [5] CAS: 2645-36-5

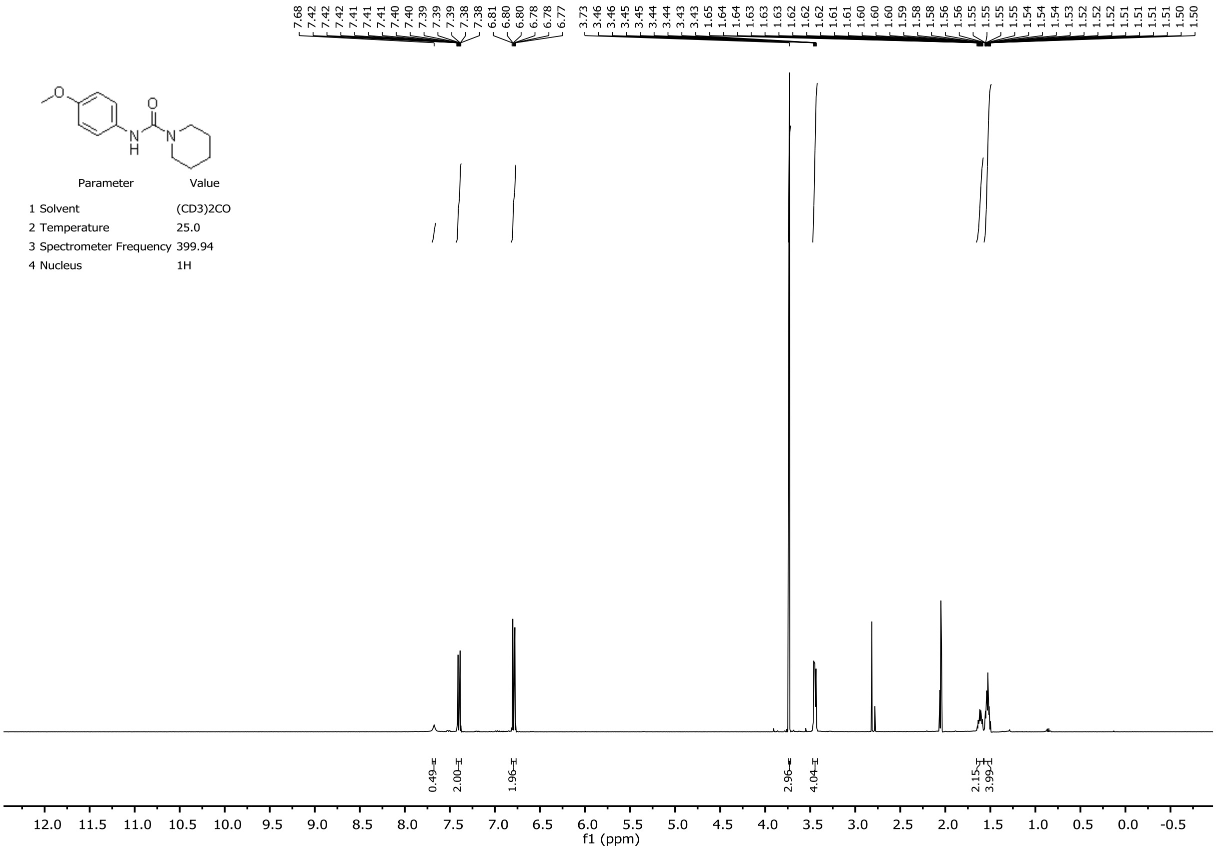

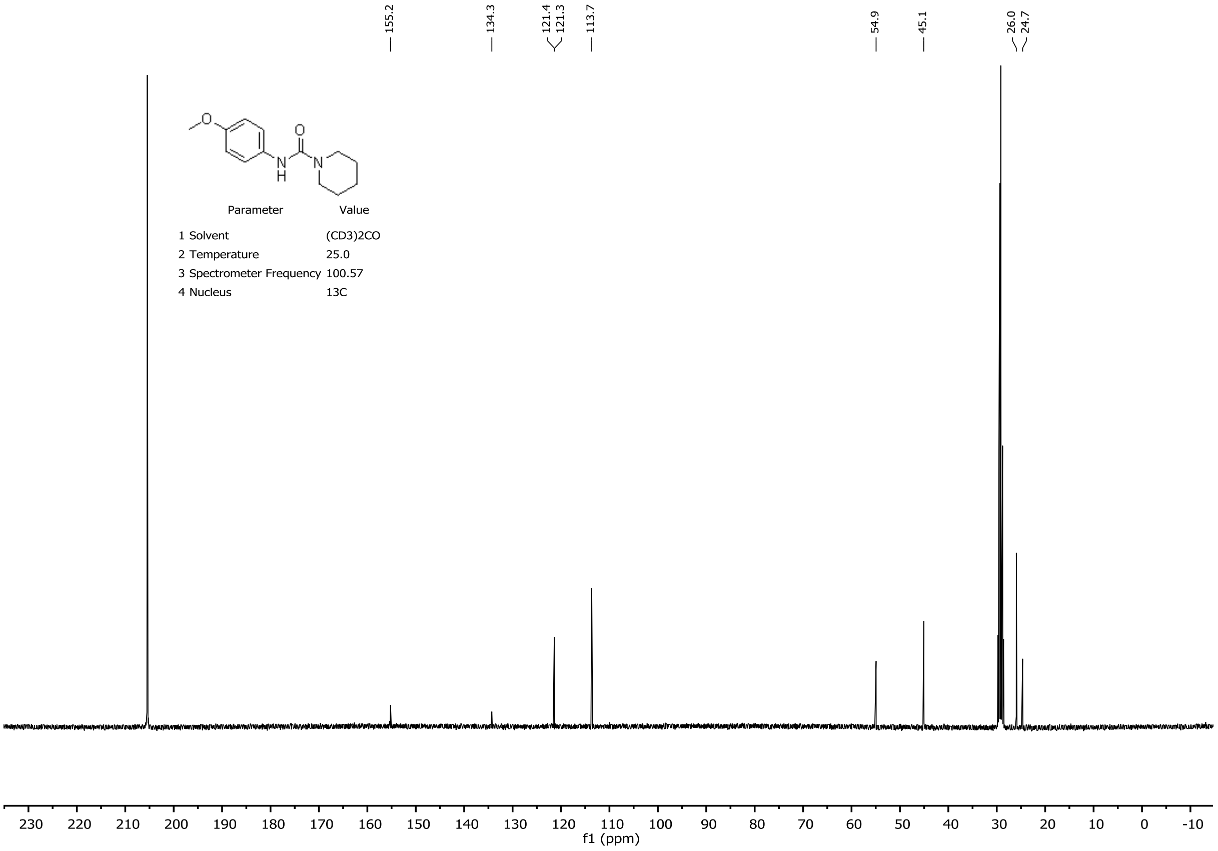

N-(4-Methoxyphenyl)piperidine-1-carboxamide CAS: 2645-37-6

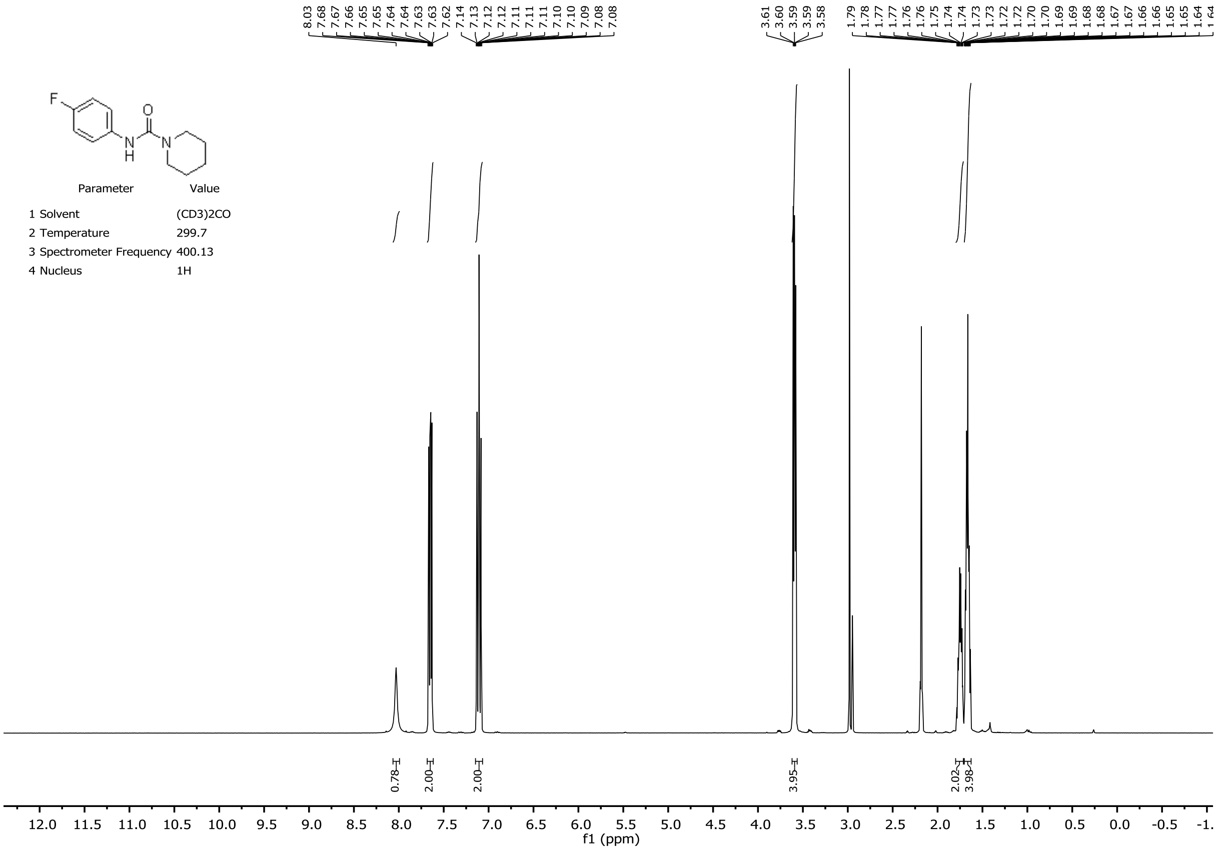

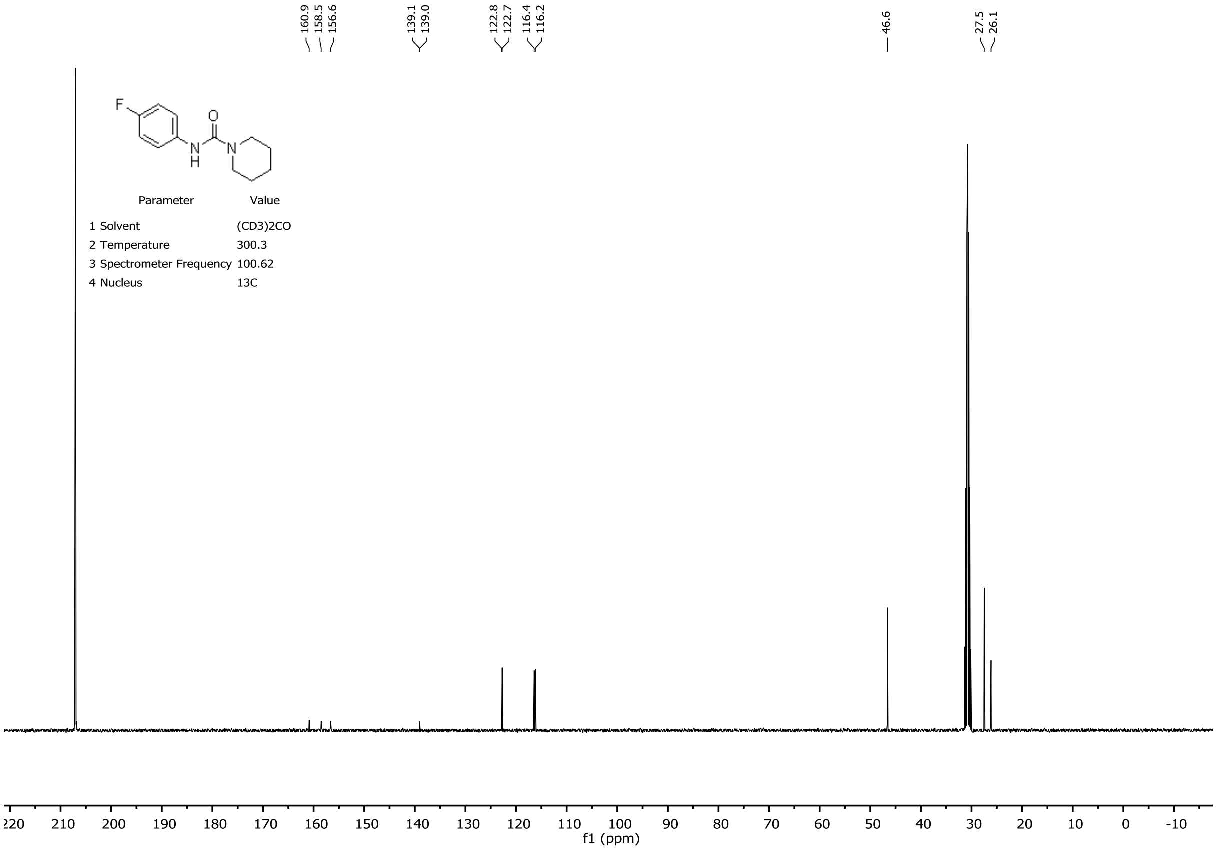

N-(4-Fluorophenyl)piperidine-1-carboxamide CAS: 60465-12-5

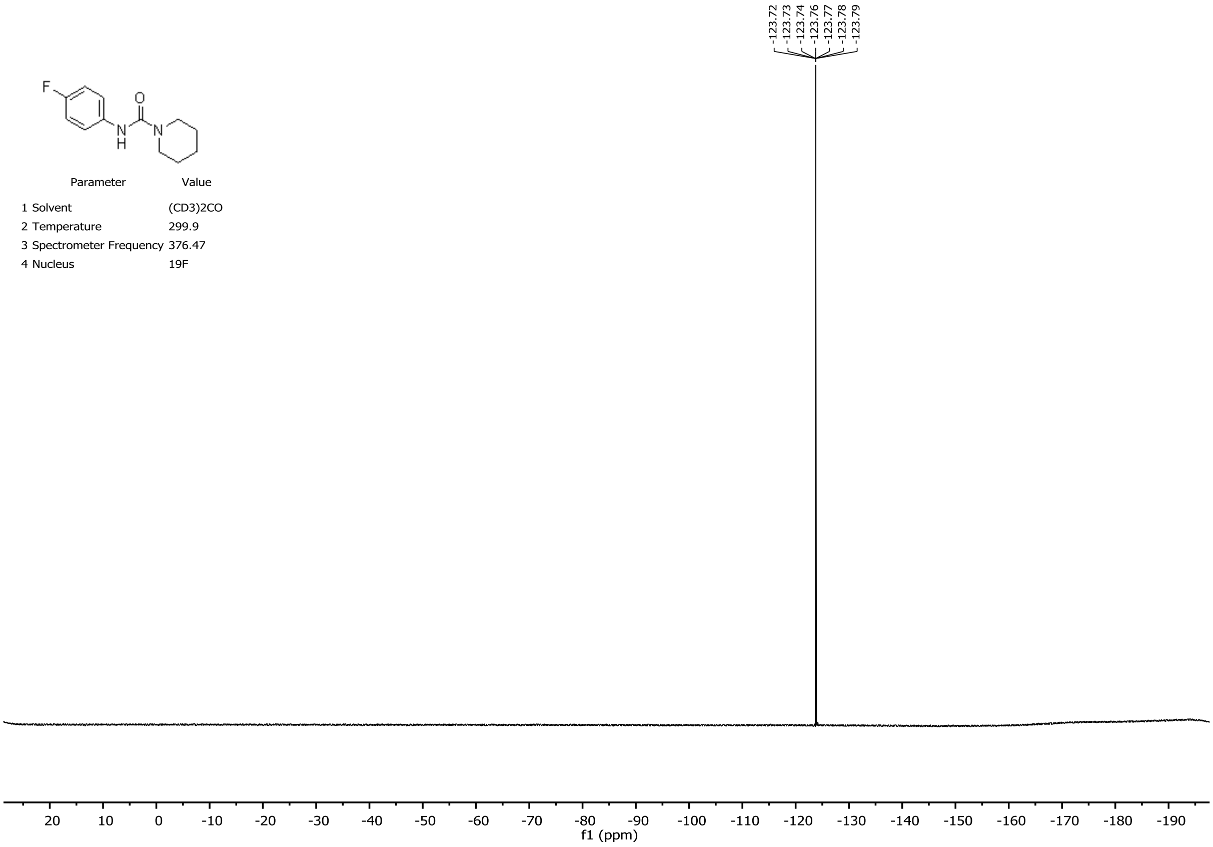

N-(4-Fluorophenyl)piperidine-1-carboxamide CAS: 60465-12-5

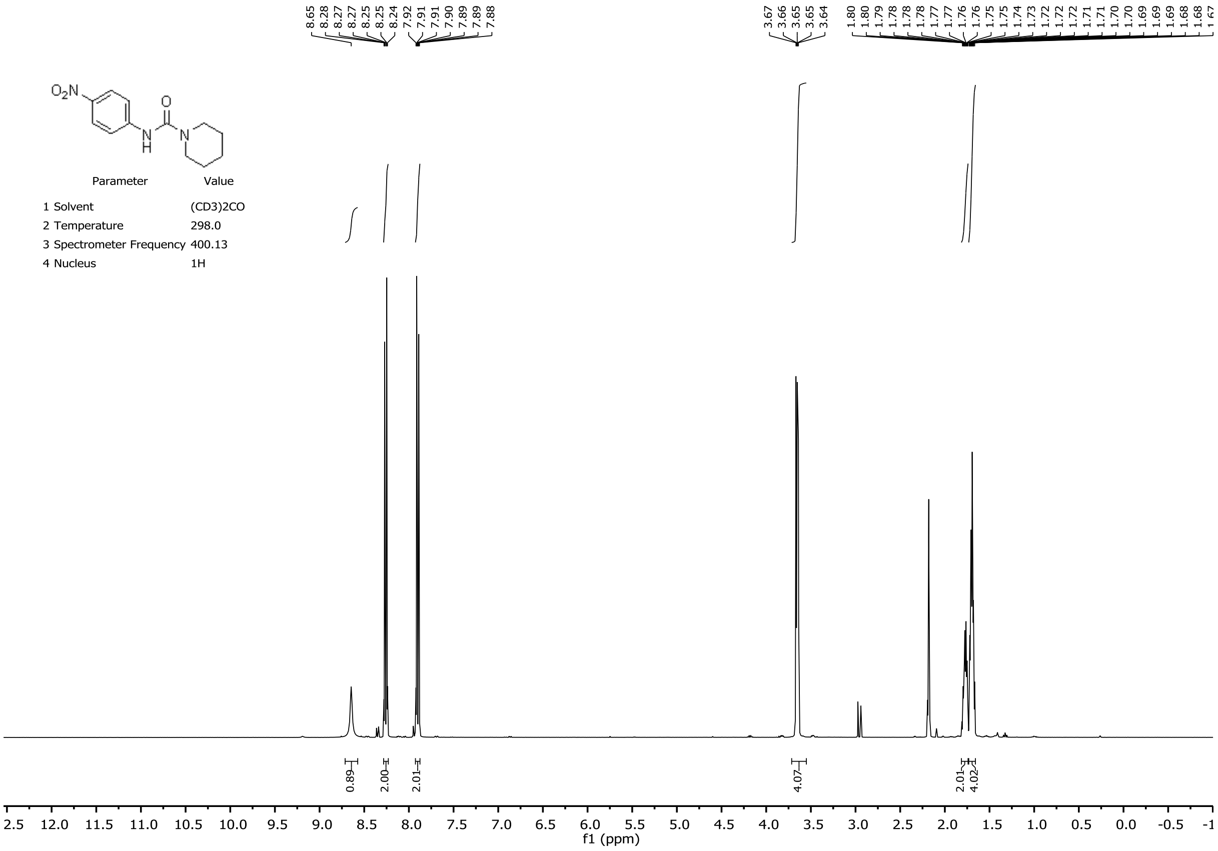

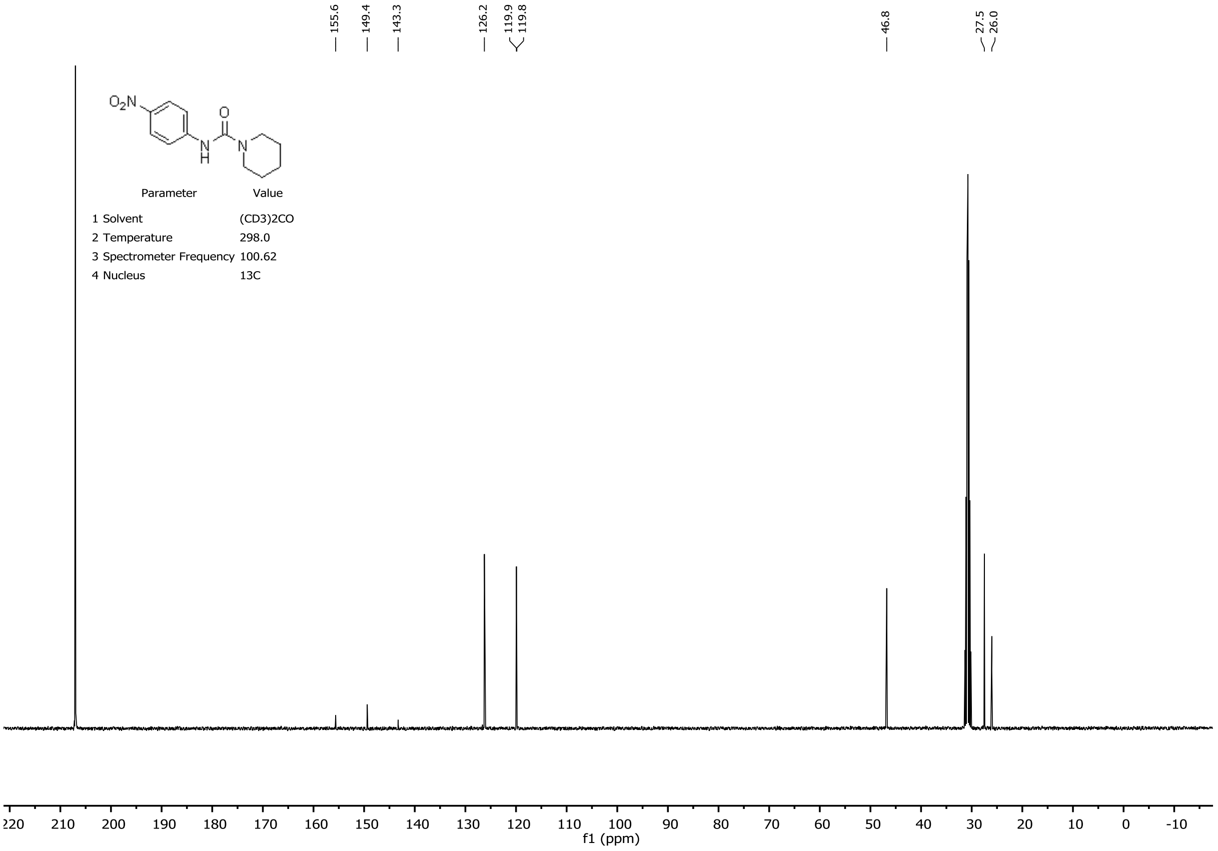

N-(4-Nitrophenyl)piperidine-1-carboxamide [6] CAS: 2589-20-0

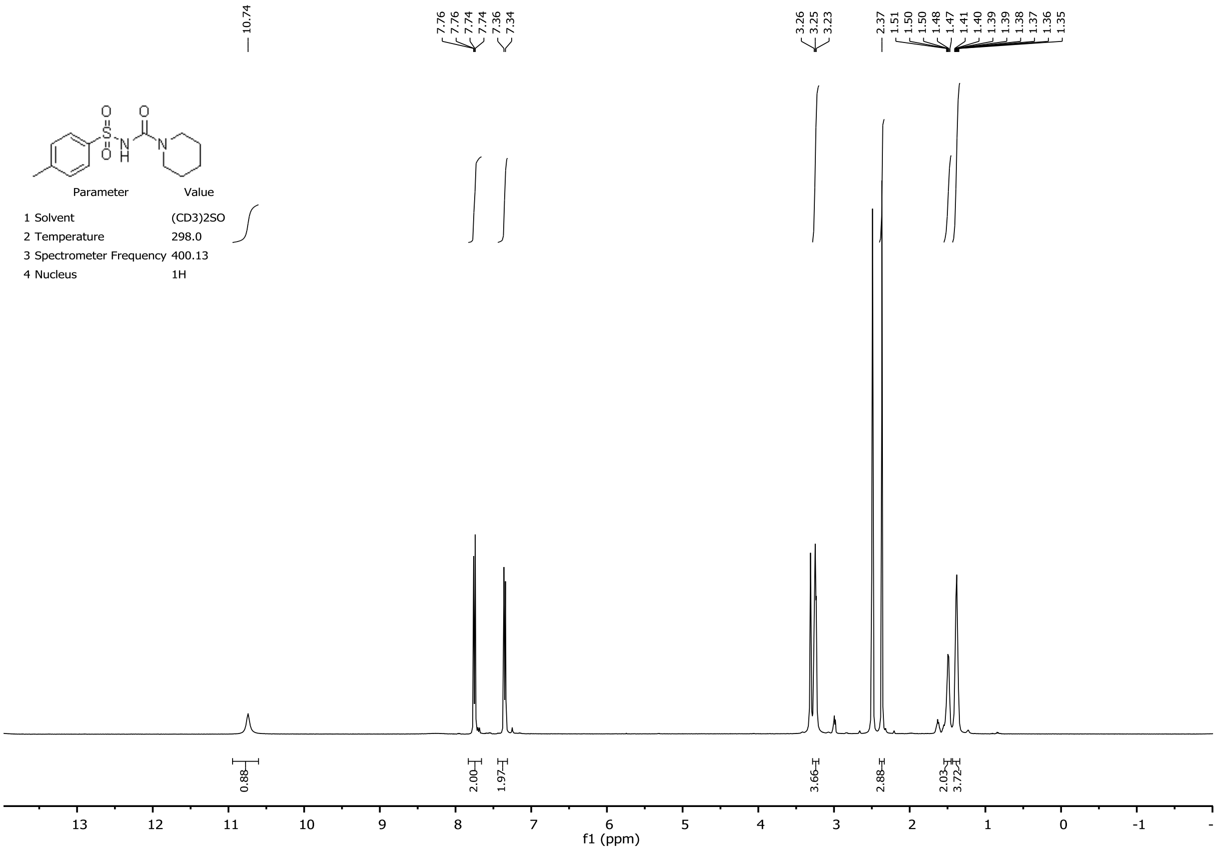

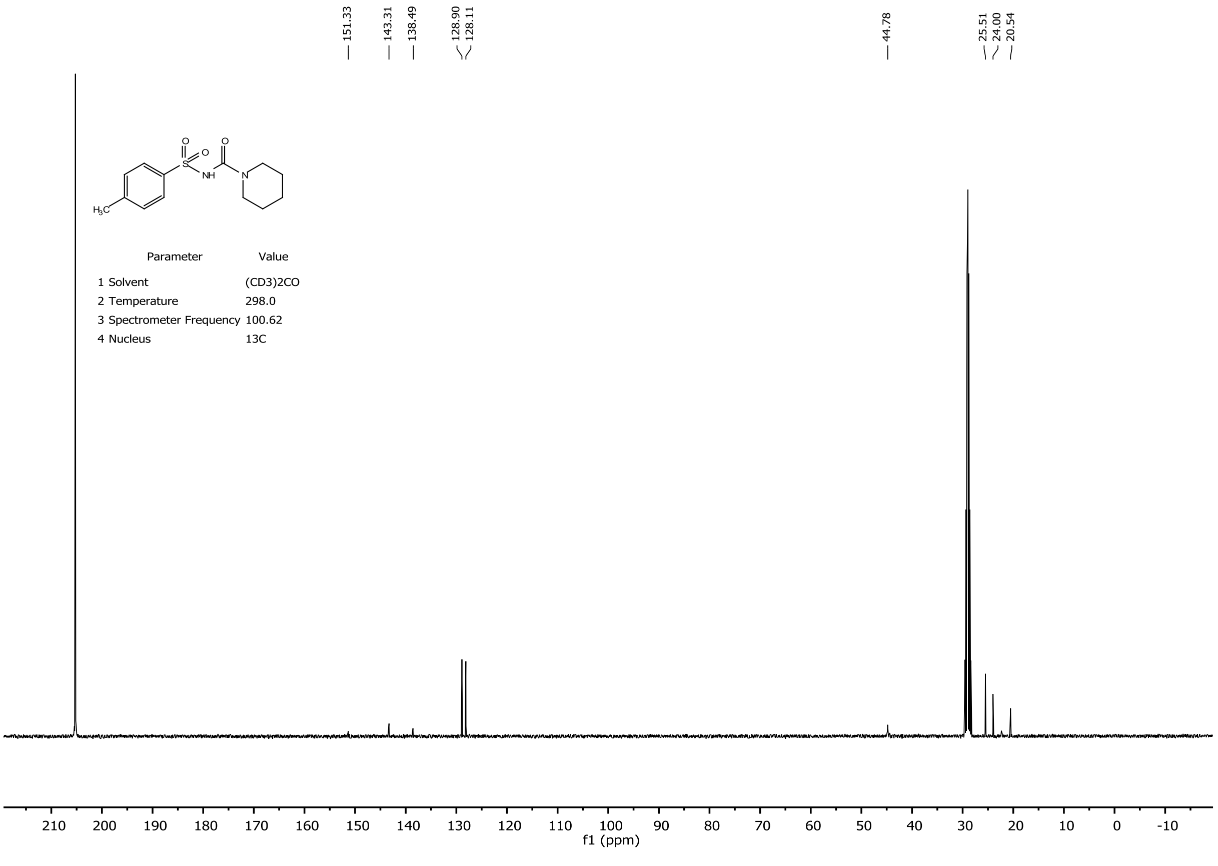

N-Tosylpiperidine-1-carboxamide CAS: 23730-08-7

HPLC Chromatogram

[carbonyl-^{11}C]N,N-dibenzylurea **2**

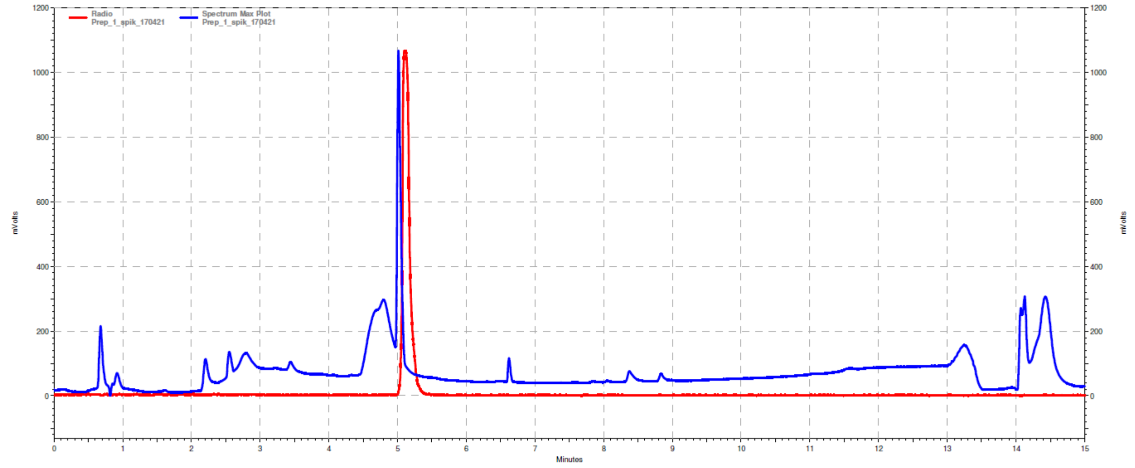

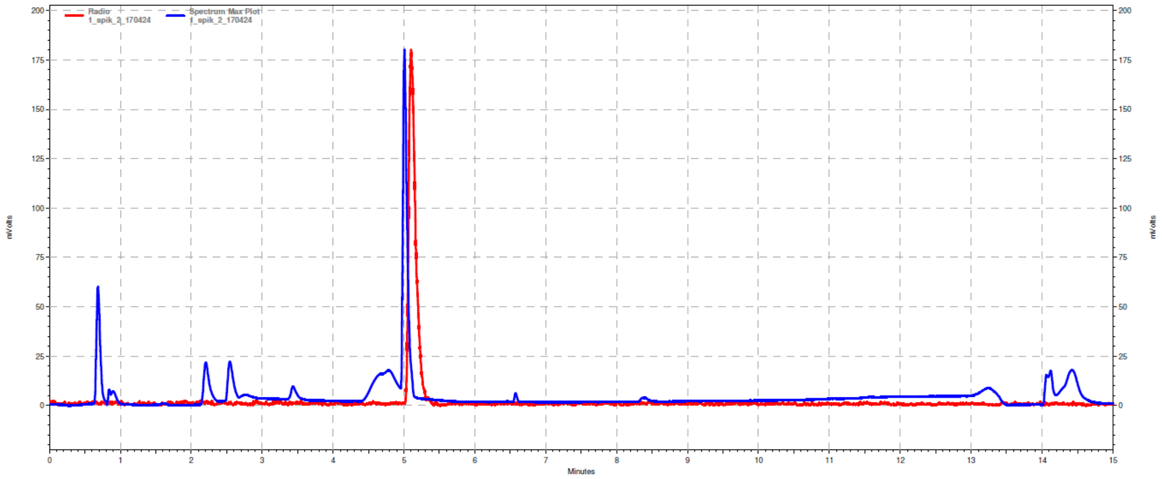

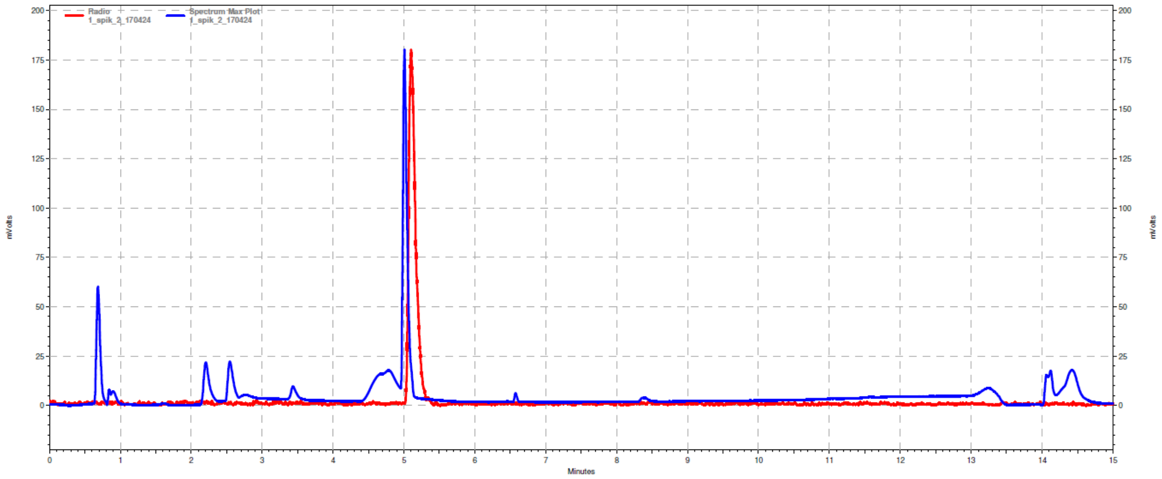

Analysis of isolated fraction containing isotopically unmodified *N,N*-dibenzylurea. Top: experiment 1; Middle: experiment 2; Bottom: experiment 3.

[carbonyl-¹¹C]N,N-dipropylurea **3**

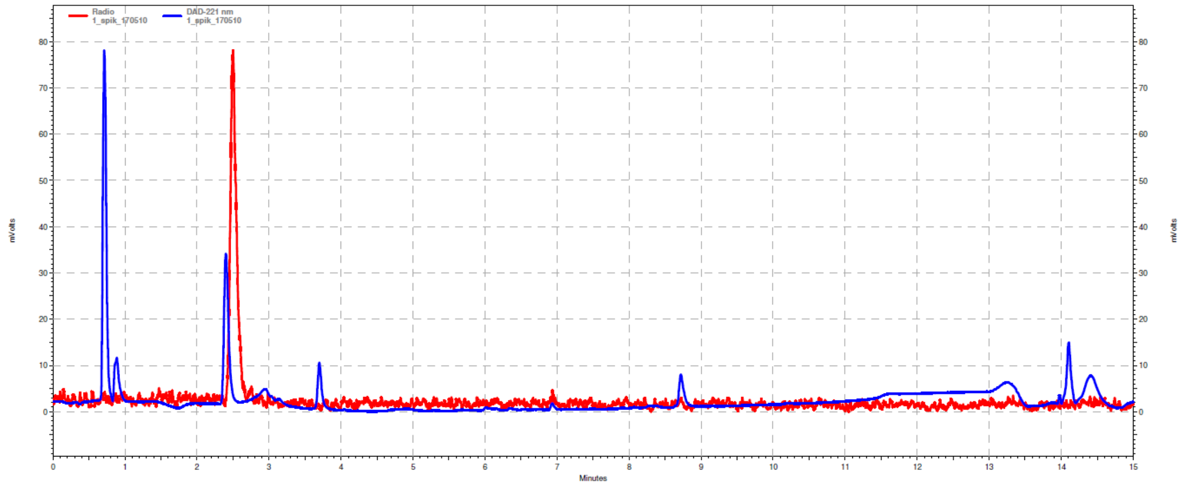

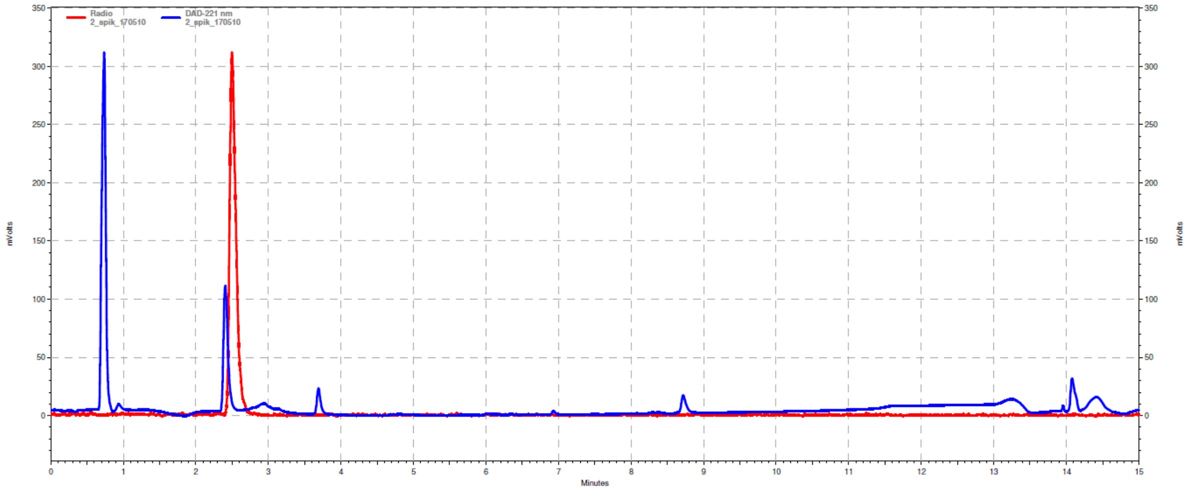

Analysis of isolated fraction containing isotopically unmodified *N,N*-dipropylurea. Top: experiment 1; Bottom: experiment 2.

[carbonyl-^{11}C]N,N-dicyclohexylurea ***4***

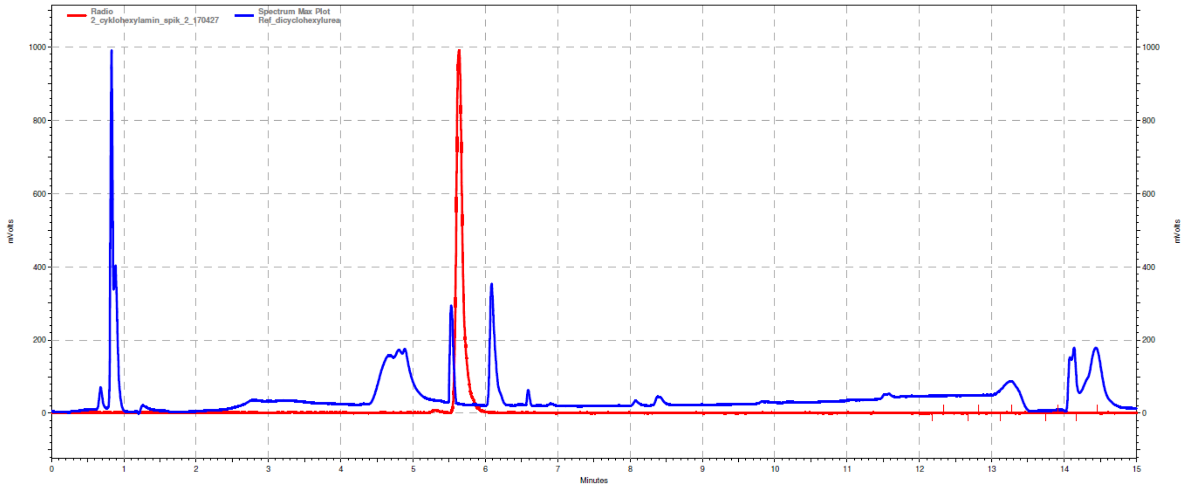

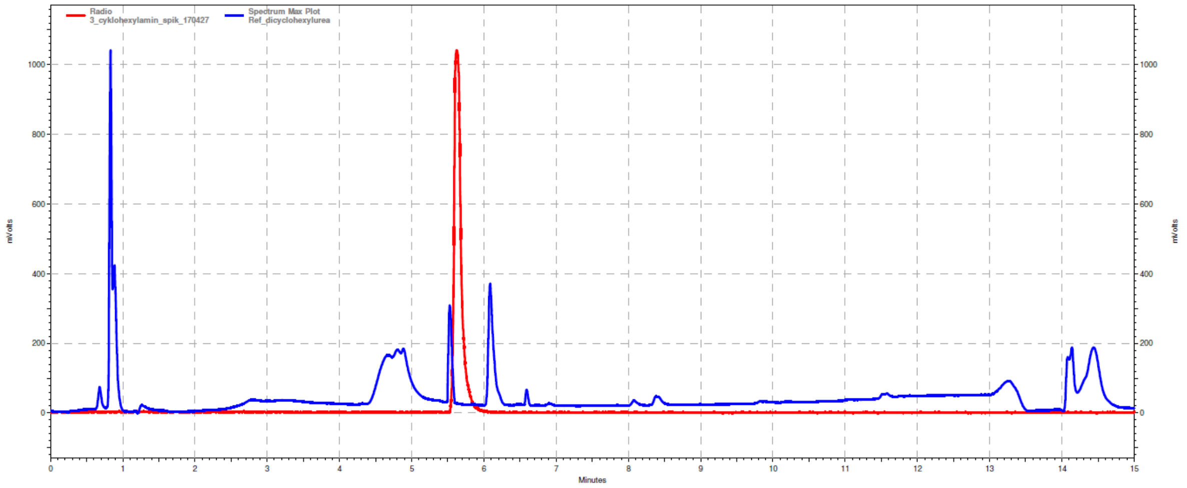

Analysis of isolated fraction containing isotopically unmodified *N,N*-dicyclohexylurea. Top: experiment 1; Bottom: experiment 2.

[carbonyl-^{11}C]N,N-diphenylurea ***5***

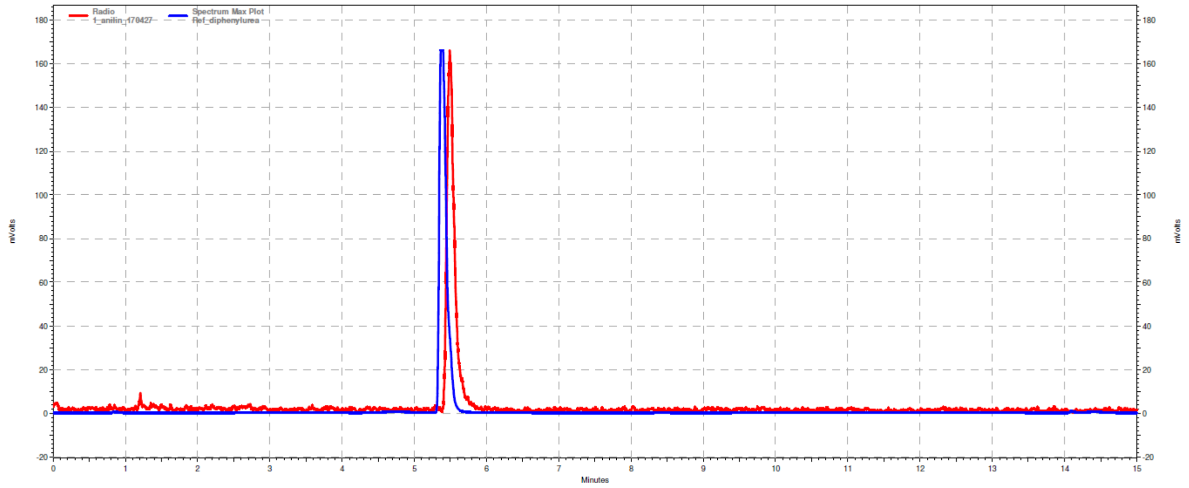

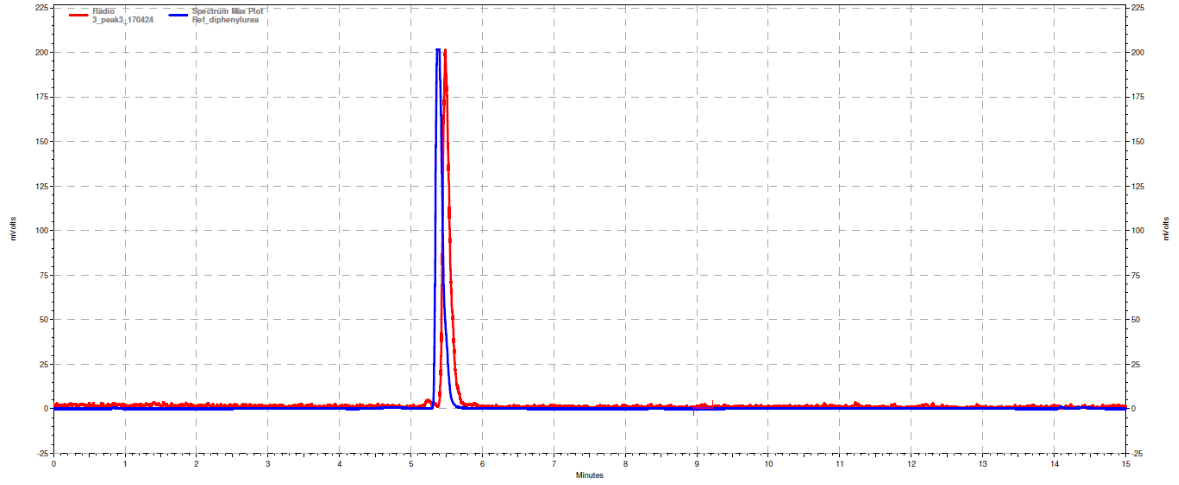

Analysis of isolated fraction containing isotopically unmodified *N,N*-diphenylurea. Top: experiment 1; Bottom: experiment 2.

[carbonyl-¹¹C]N-benzylpiperidine-1-carboxamide **7**

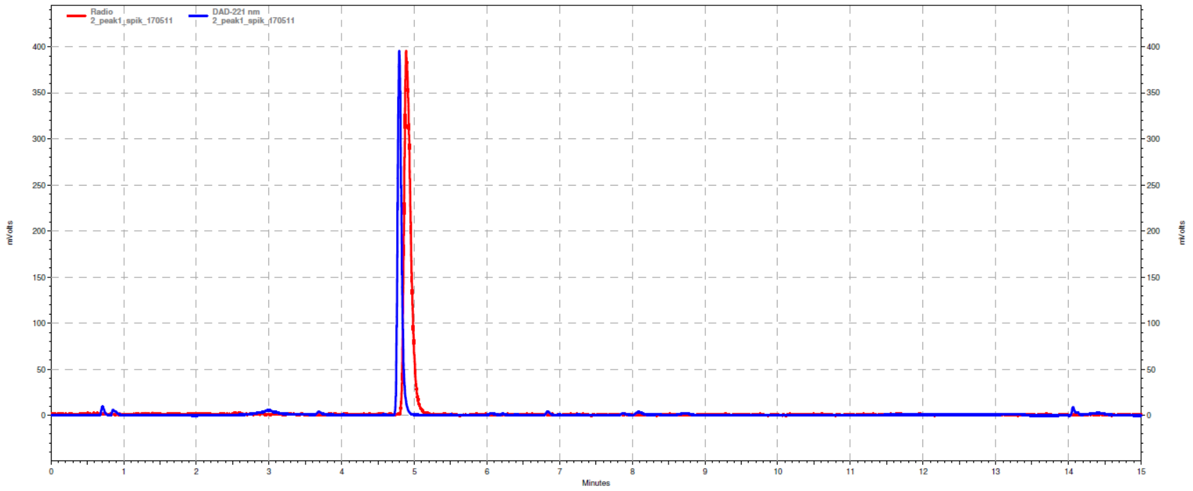

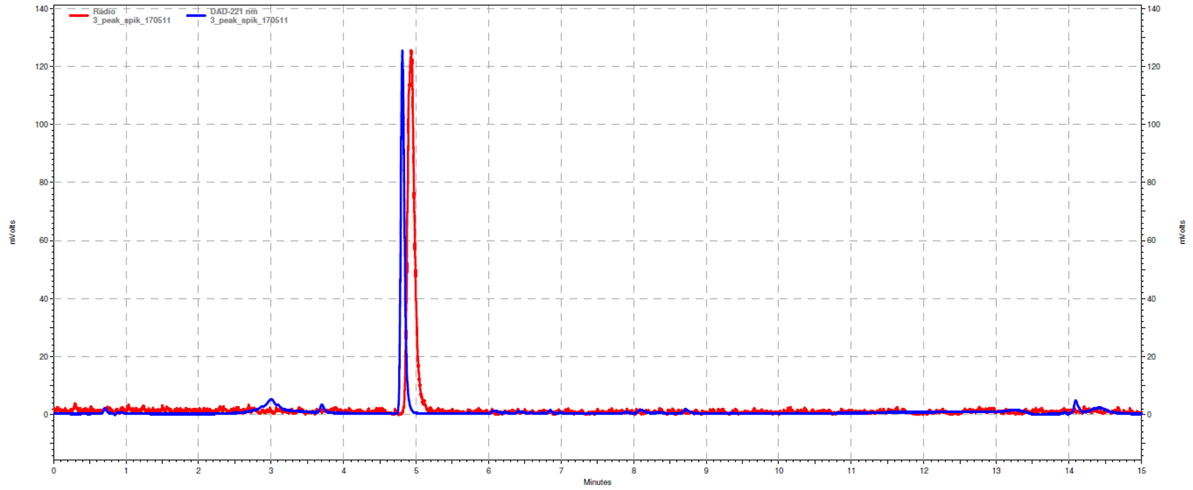

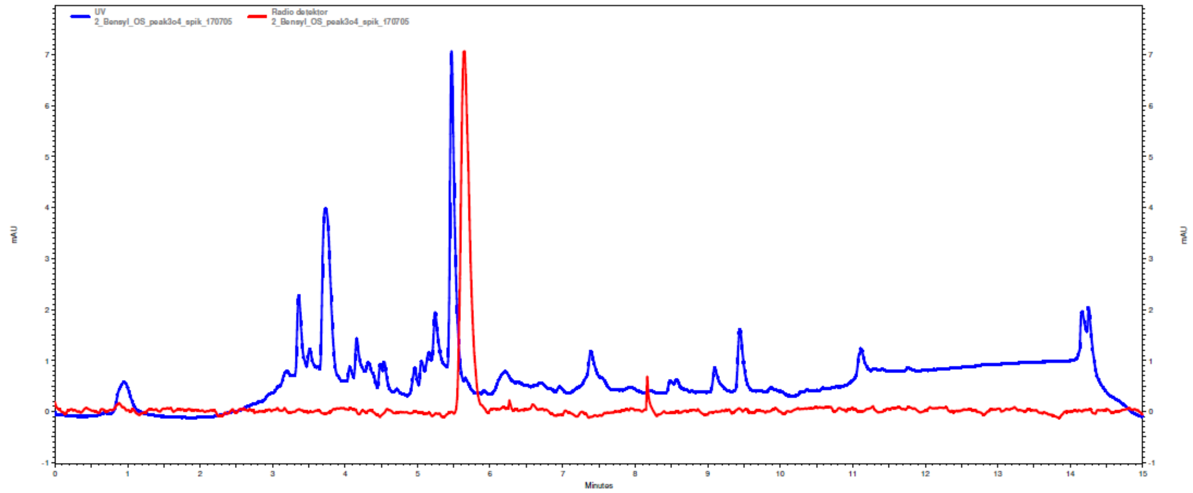

Analysis of isolated fraction containing isotopically unmodified *N*-benzylpiperidine-1-carboxamide. Top: experiment 1; Middle: experiment 2; Bottom: experiment 3.

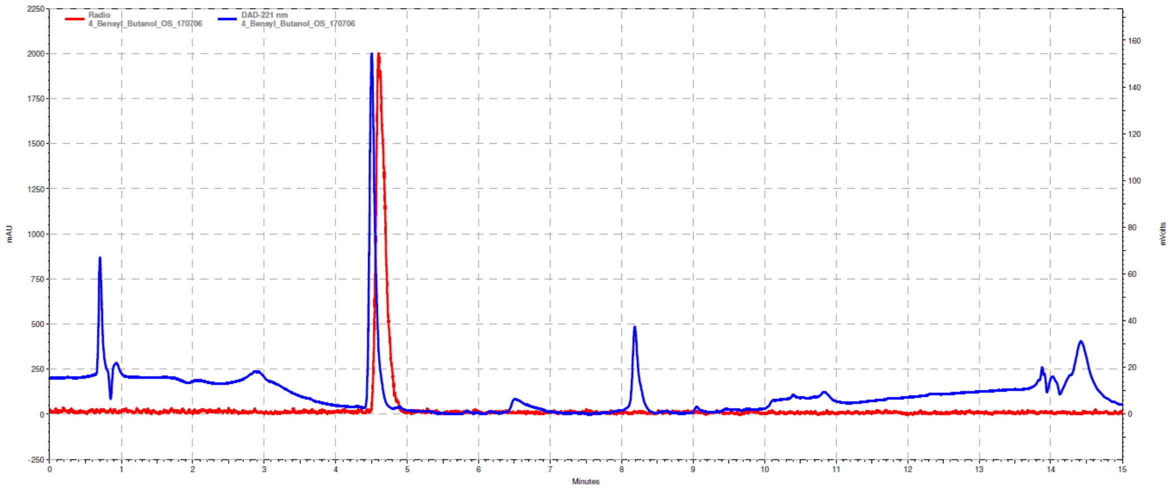

Analysis of isolated fraction containing isotopically unmodified *N*-benzylpiperidine-1-carboxamide. Bottom: experiment 4.

[carbonyl-^{11}C]N-butylpiperidine-1-carboxamide **8**

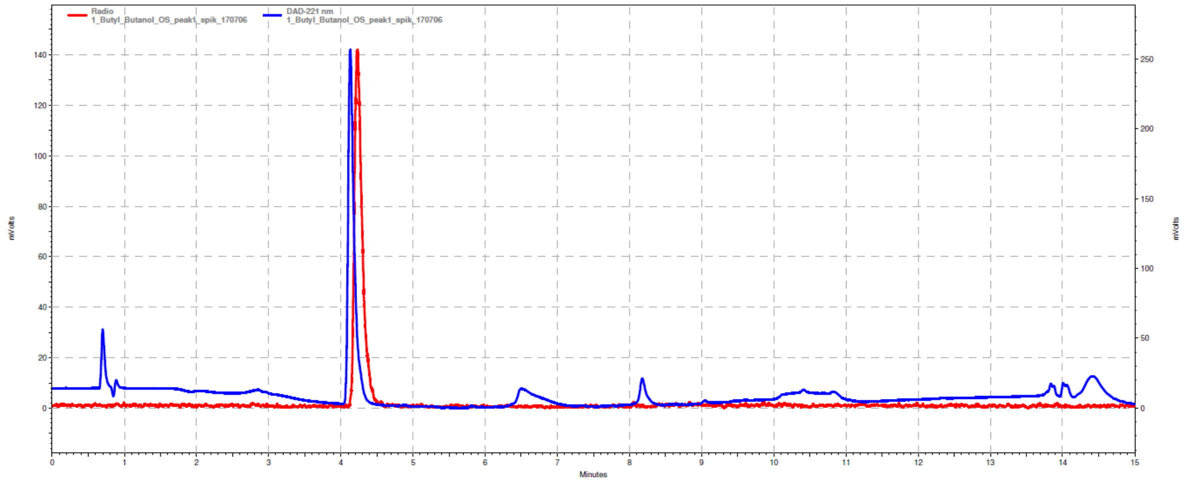

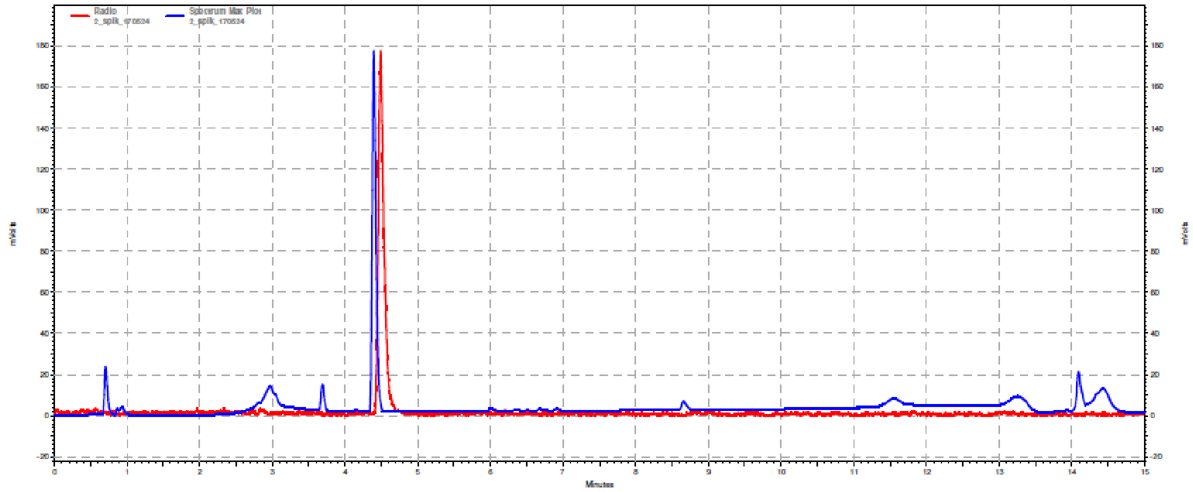

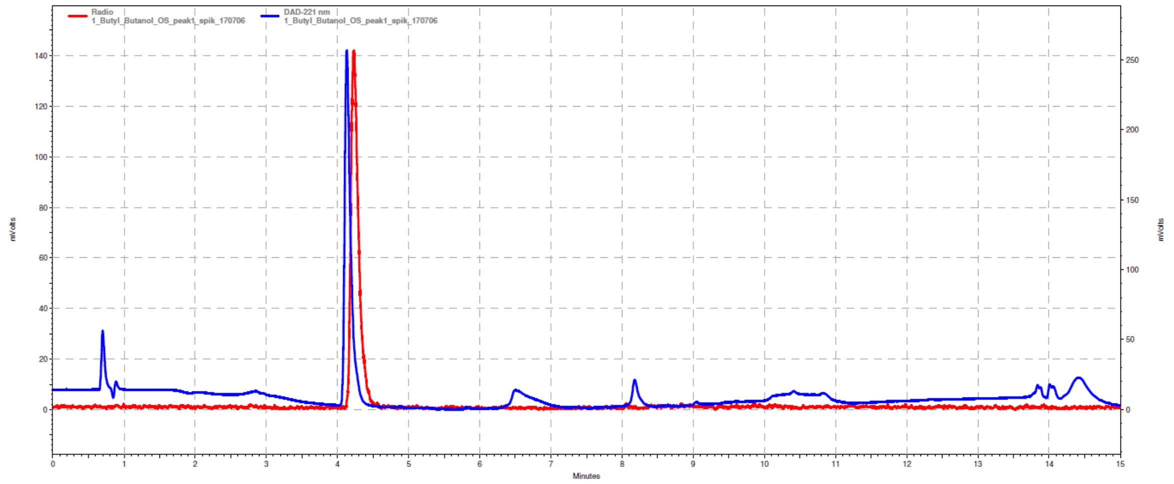

Analysis of isolated fraction containing isotopically unmodified *N*-butylpiperidine-1-carboxamide. Top: experiment 1; Middle: experiment 2; Bottom: experiment 3.

[carbonyl-^{11}C]N-(2-(pyridin-2-yl)ethyl)piperidine-1-carboxamide ***9***

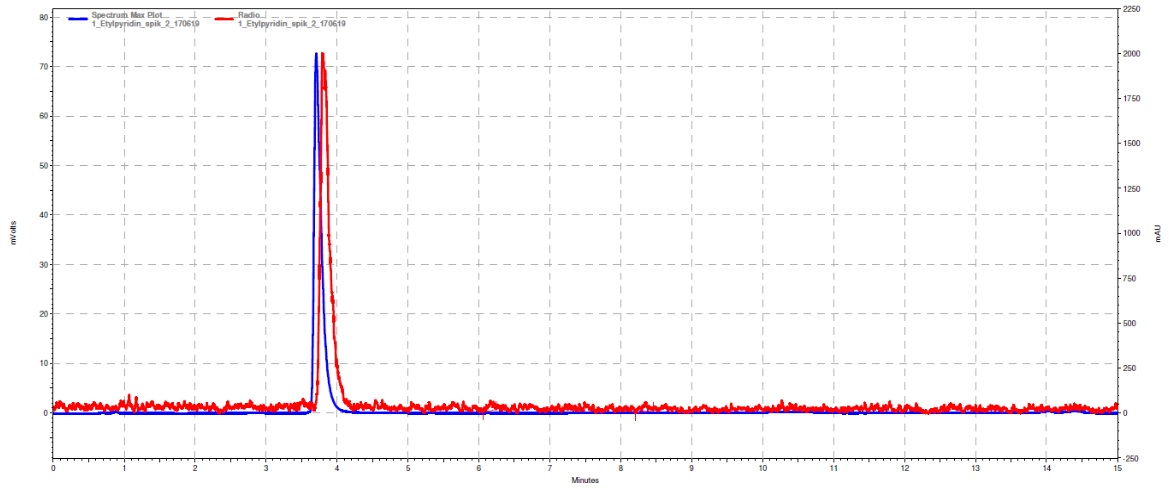

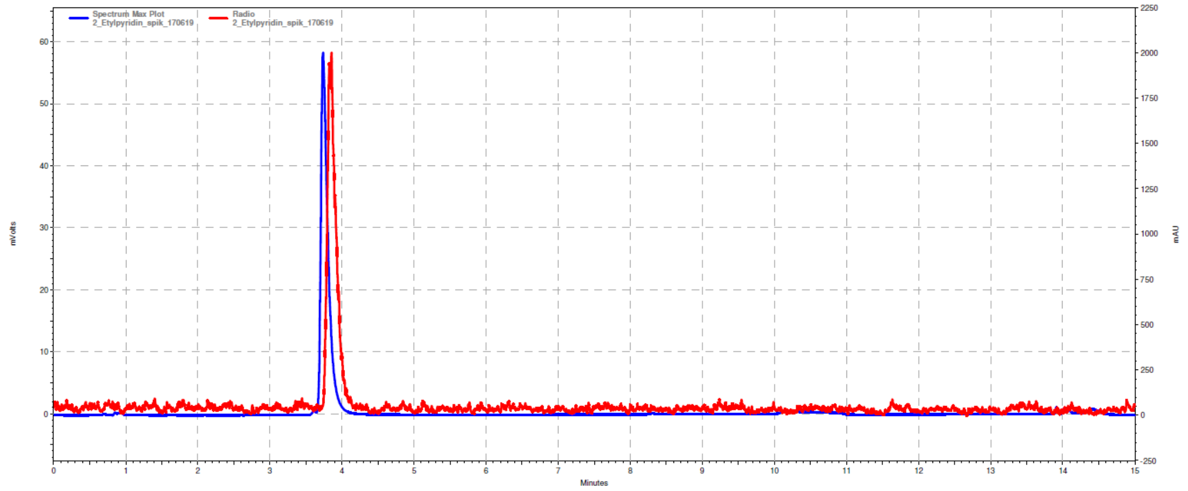

Analysis of isolated fraction containing isotopically unmodified *N*-(2-(pyridin-2-yl)ethyl)piperidine-1-carboxamide. Top: experiment 1; Bottom: experiment 2.

[carbonyl-^{11}C]N-isopropylpiperidine-1-carboxamide ***10***

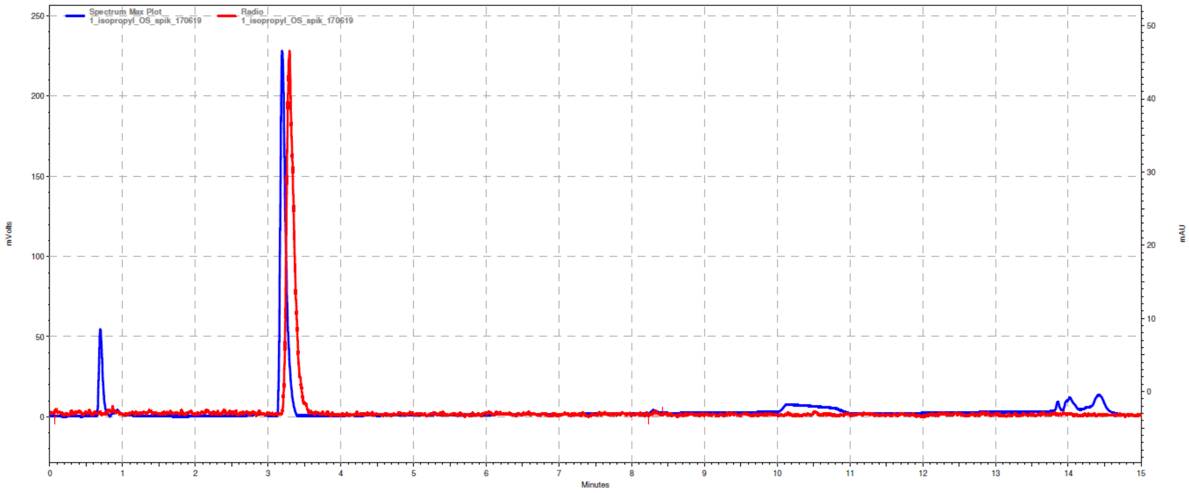

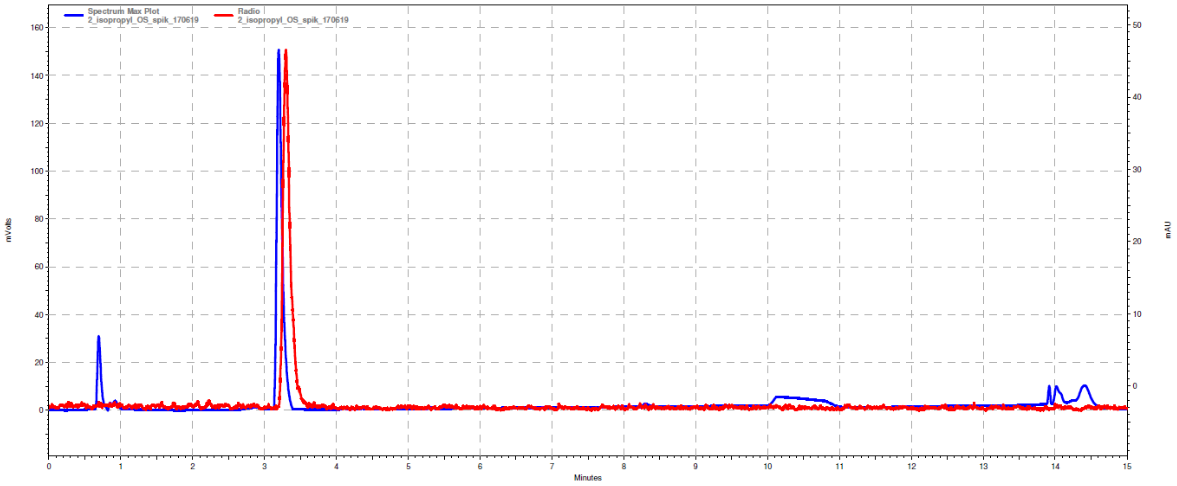

Analysis of isolated fraction containing isotopically unmodified *N*-isopropylpiperidine-1-carboxamide. Top: experiment 1; Bottom: experiment 2.

[carbonyl-¹¹C]N-phenylpiperidine-1-carboxamide ***11***

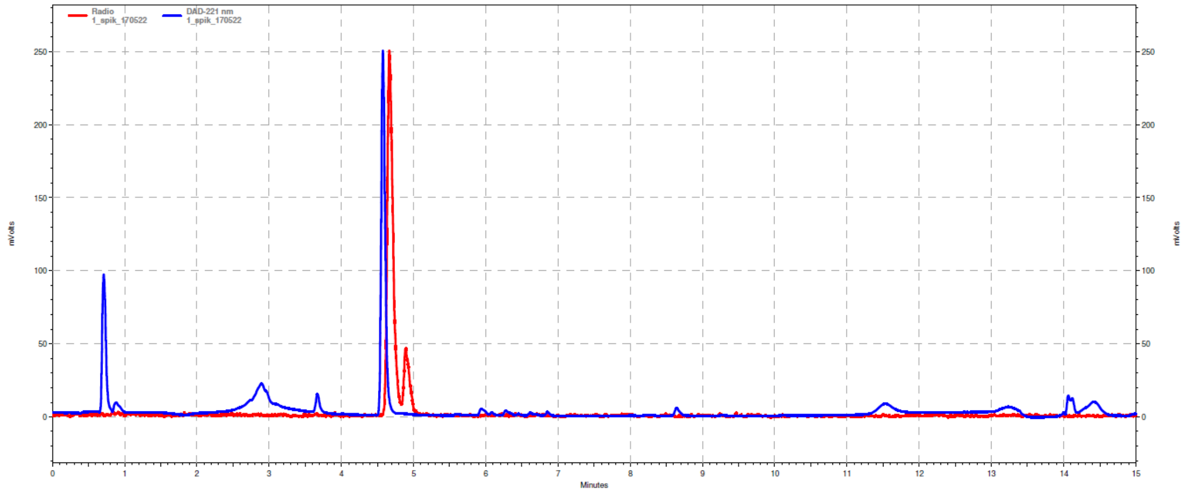

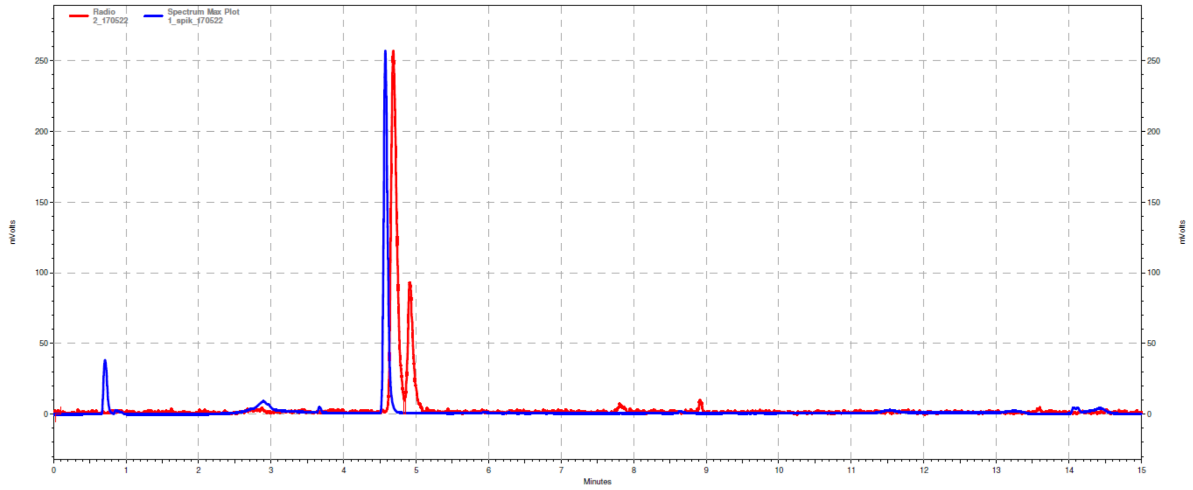

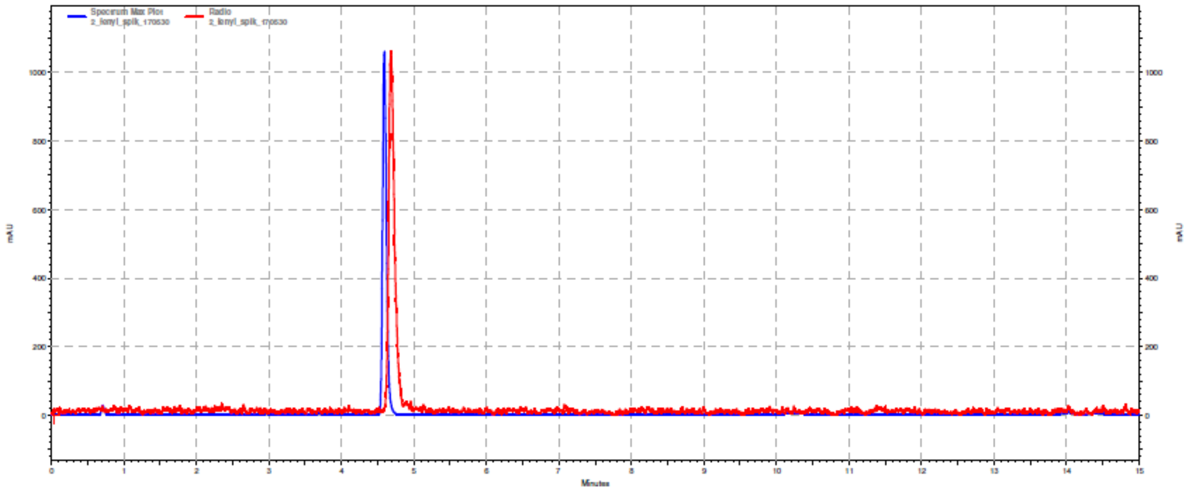

Analysis of isolated fraction containing isotopically unmodified *N*-phenylpiperidine-1-carboxamide. Top: experiment 1; Middle: experiment 2; Bottom: experiment 3.

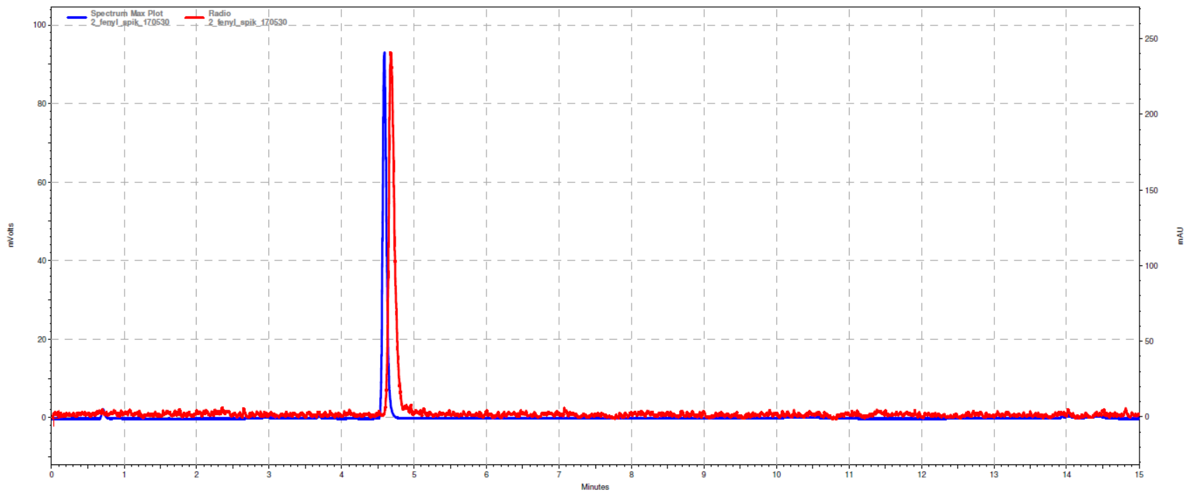

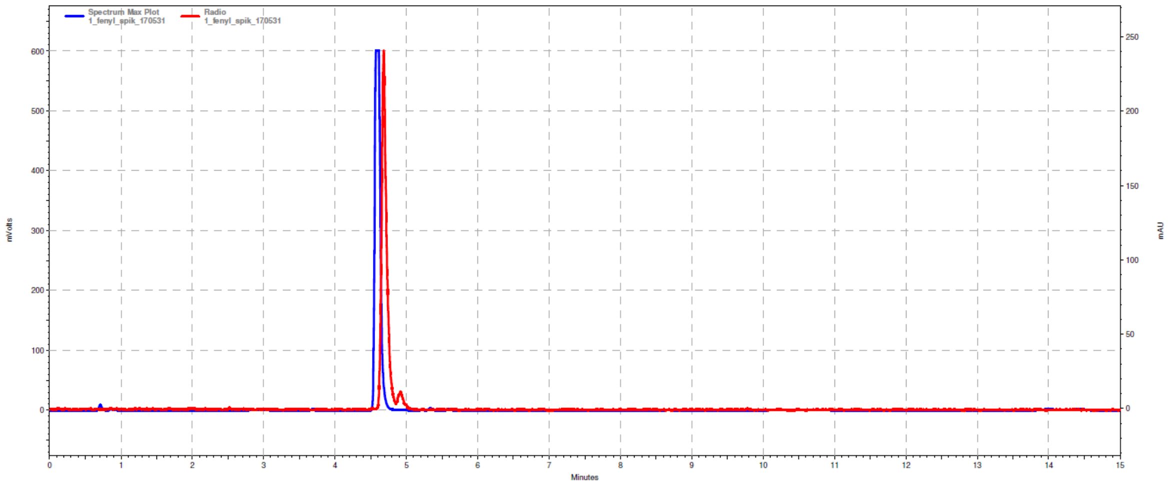

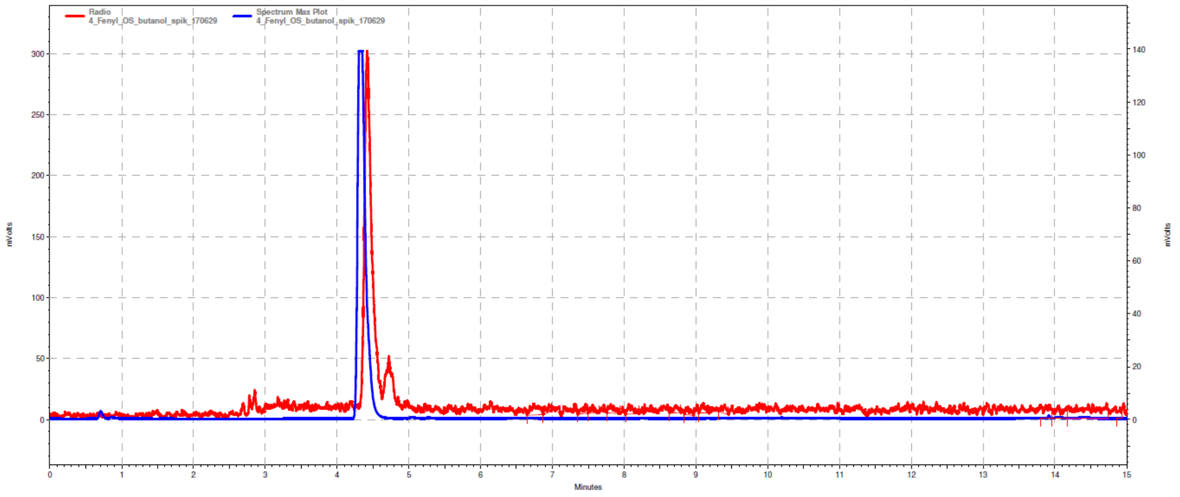

Analysis of isolated fraction containing isotopically unmodified *N*-phenylpiperidine-1-carboxamide. Top: experiment 4; Middle: experiment 5; Bottom: experiment 6.

[carbonyl-^{11}C]N-(4-methoxyphenyl)piperidine-1-carboxamide ***12***

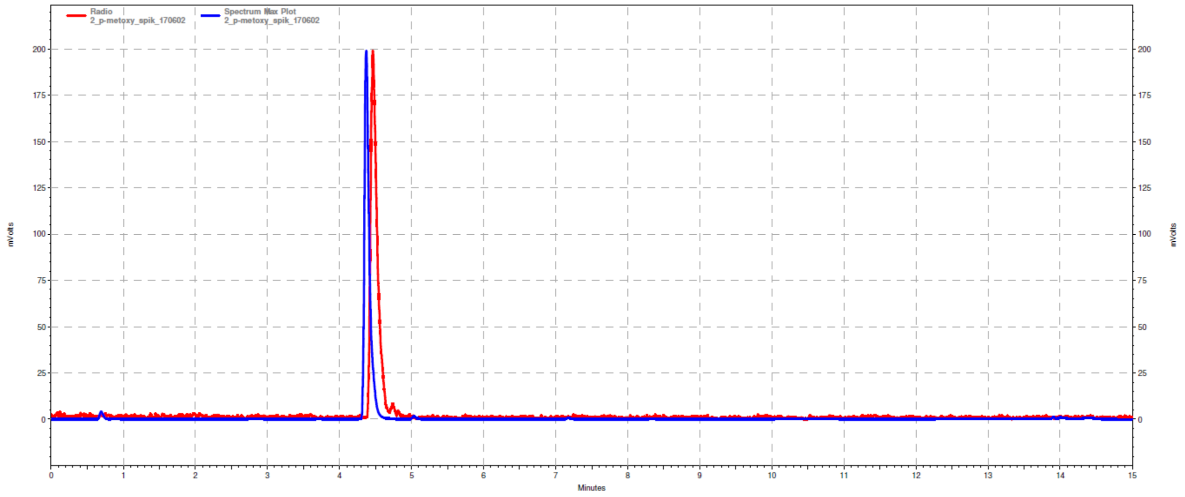

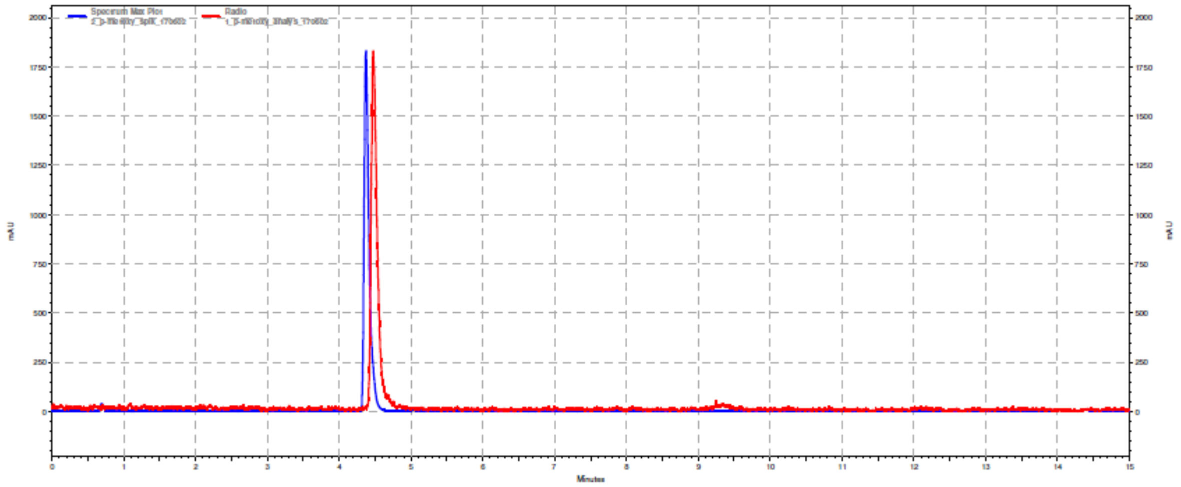

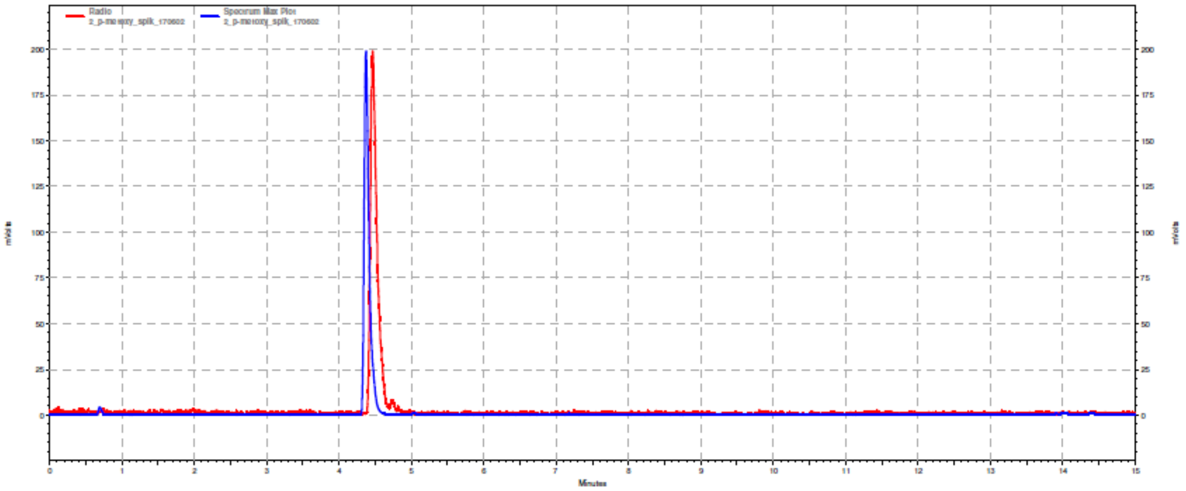

Analysis of isolated fraction containing isotopically unmodified *N*-(4-methoxyphenyl)piperidine-1-carboxamide. Top: experiment 1; Middle: experiment 2; Bottom: experiment 3.

[carbonyl-^{11}C]N-(4-fluorophenyl)piperidine-1-carboxamide ***13***

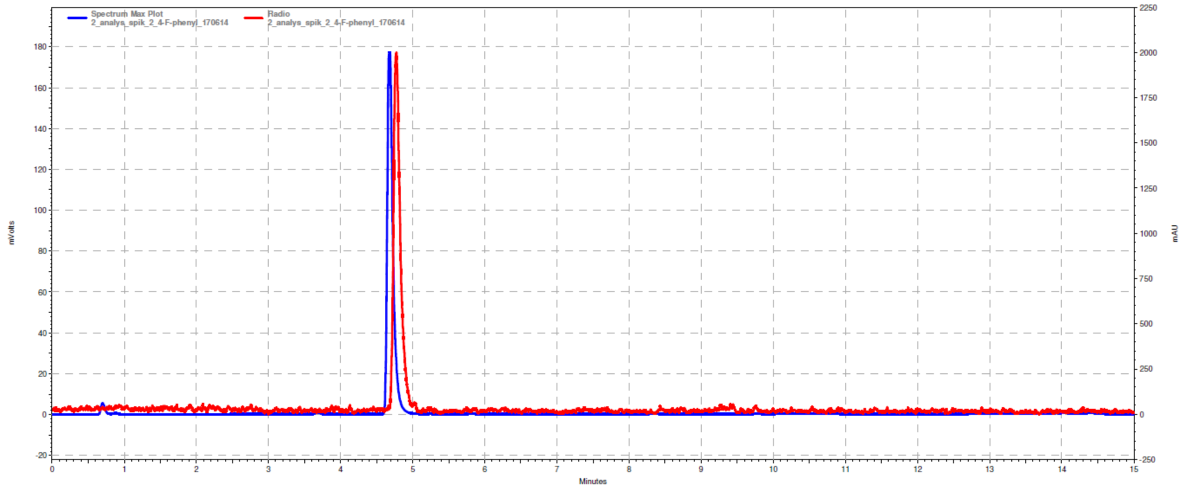

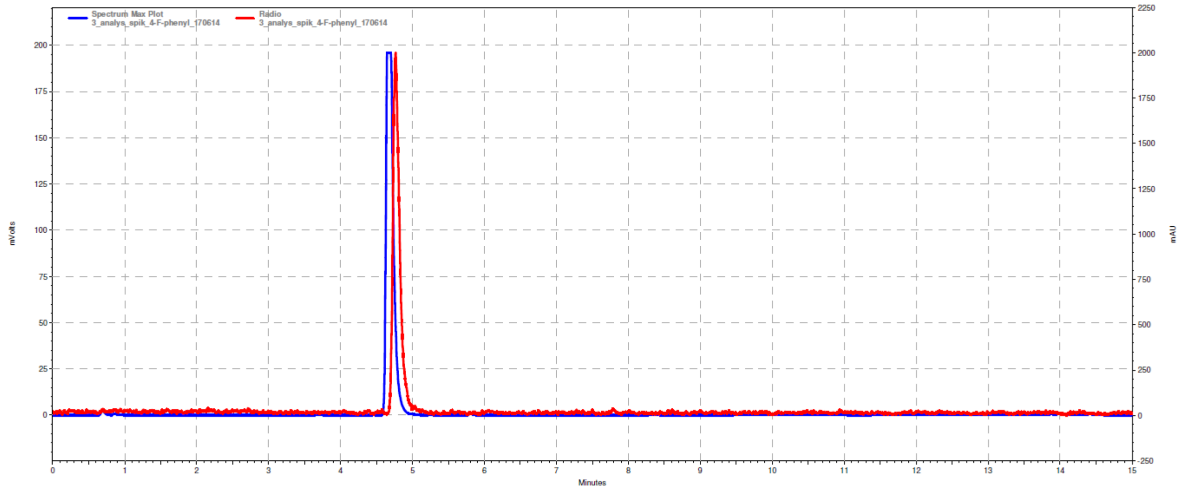

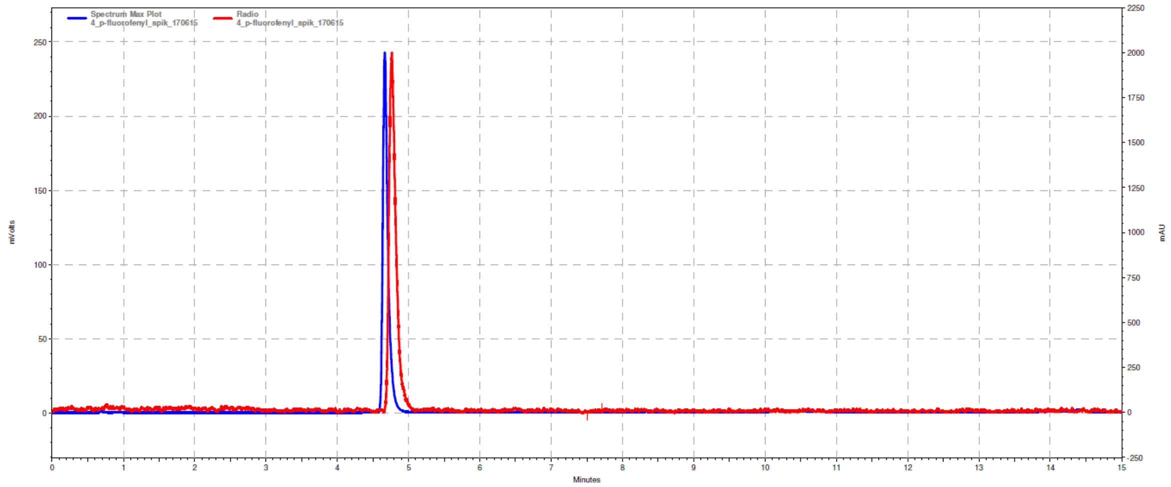

Analysis of isolated fraction containing isotopically unmodified *N*-(4-fluorophenyl)-piperidine-1-carboxamide. Top: experiment 1; Middle: experiment 2; Bottom: experiment 3.

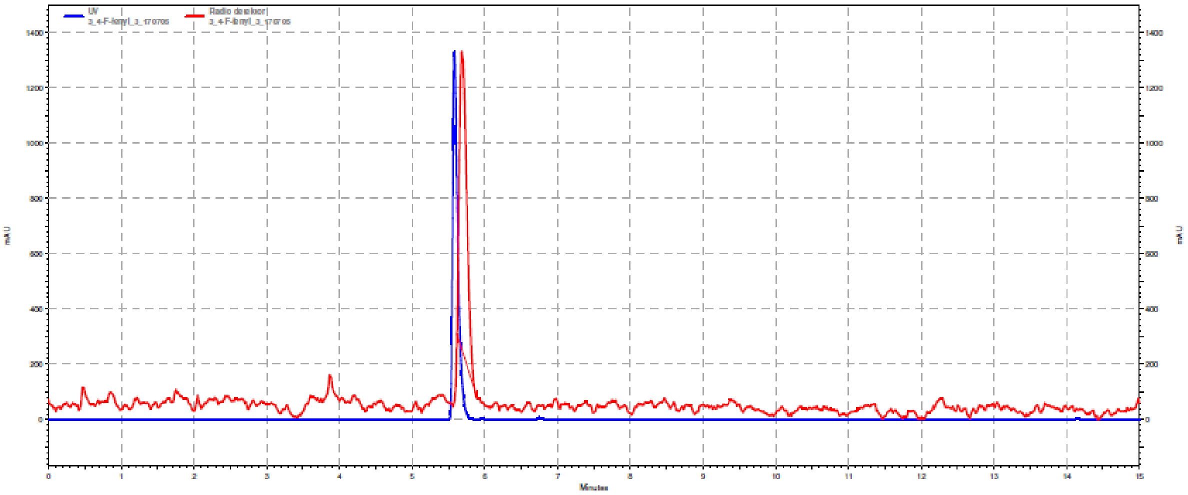

Analysis of isolated fraction containing isotopically unmodified *N*-(4-fluorophenyl)piperidine-1-carboxamide. Experiment 4.

[carbonyl-^{11}C]N-(4-nitrophenyl)piperidine-1-carboxamide ***14***

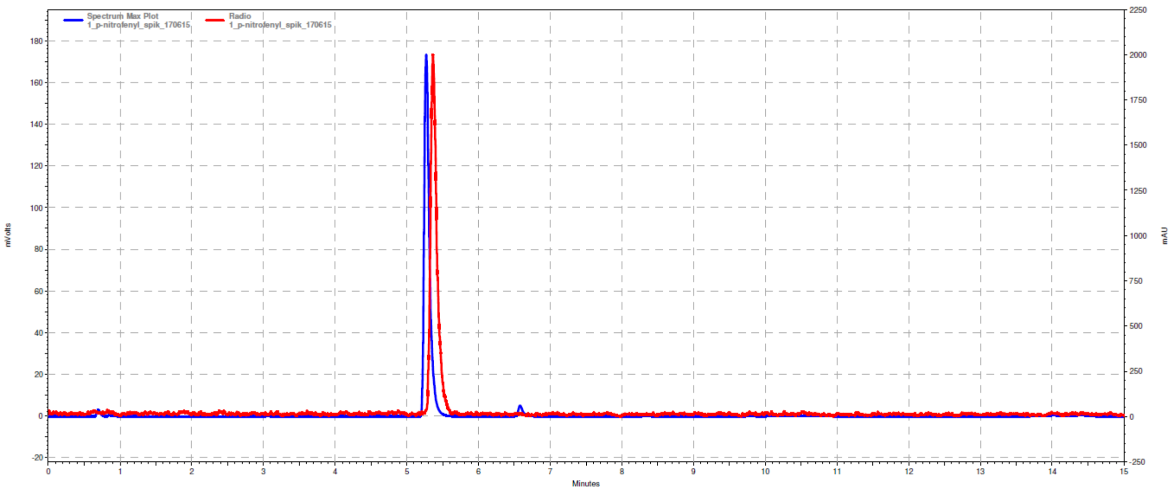

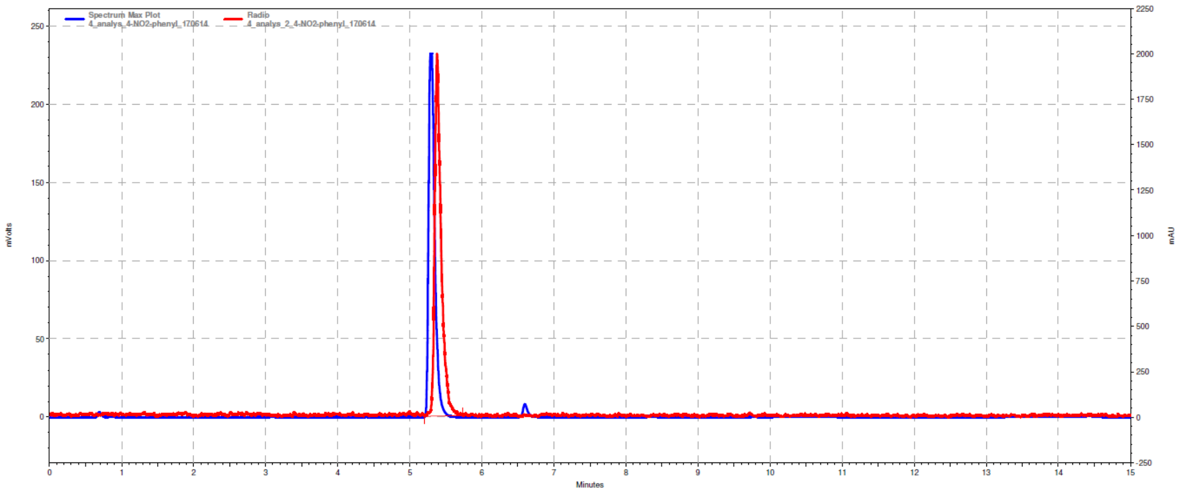

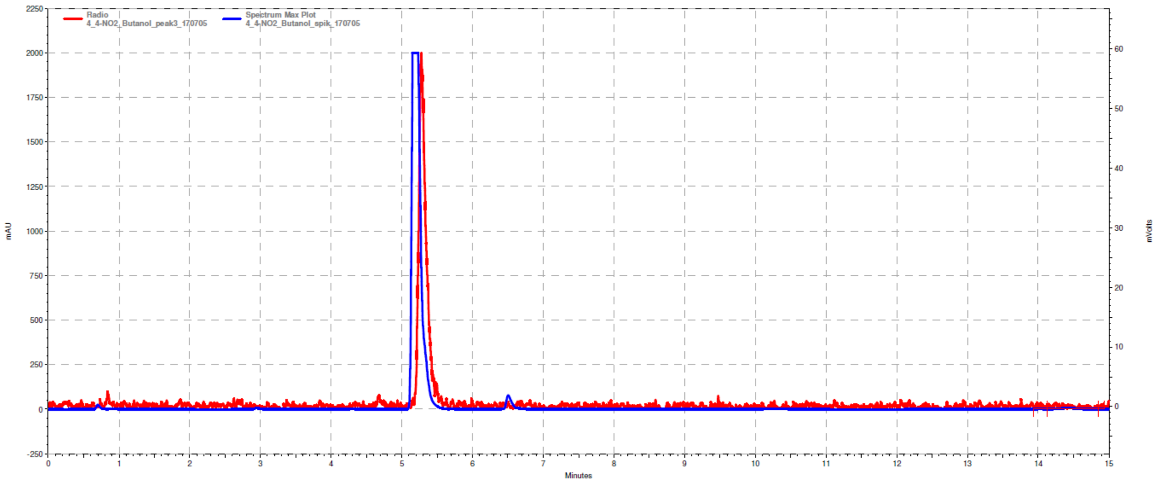

Analysis of isolated fraction containing isotopically unmodified *N*-(4-nitrophenyl)piperidine-1-carboxamide. Top: experiment 1; Middle: experiment 2; Bottom: experiment 3.

[carbonyl-^{11}C]3,4-dihydroquinazolin-2(1H)-one ***15***

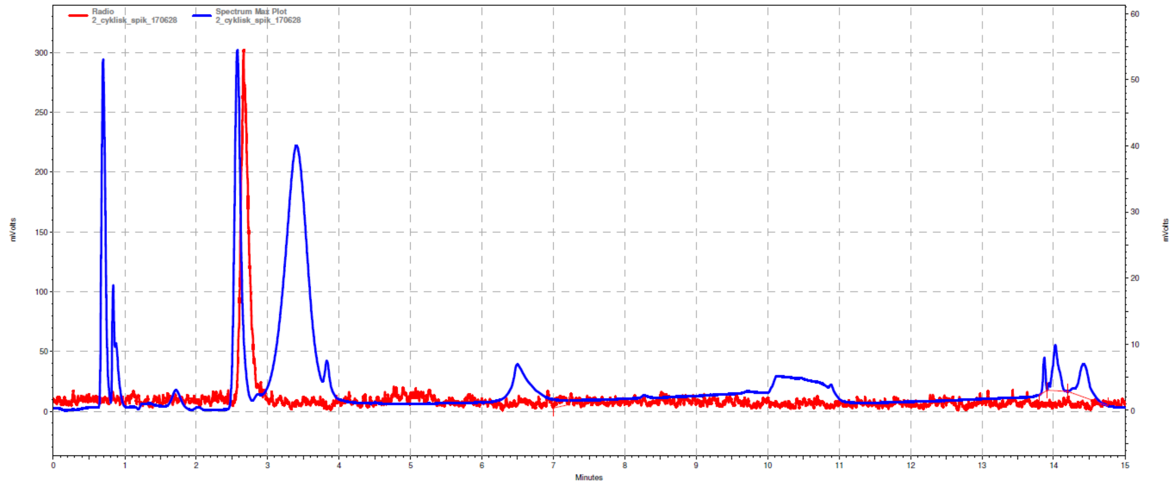

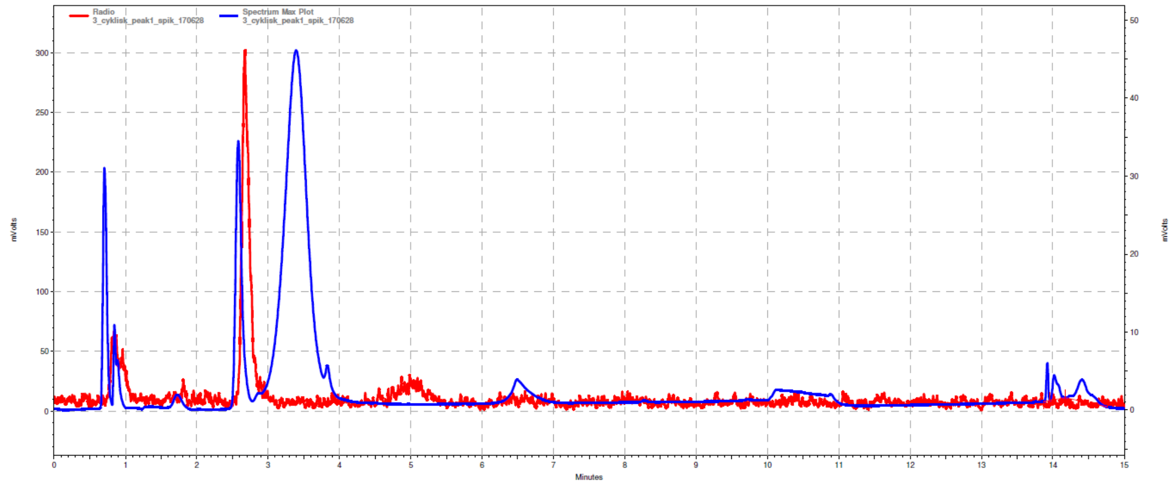

Analysis of isolated fraction containing isotopically unmodified 3,4-dihydroquinazolin-2(1H)-one. Top: experiment 1; Bottom: experiment 2.

[carbonyl-^{11}C]N-(2,4-dichlorobenzyl)-4-phenoxypiperidine-1-carboxamide ***19***

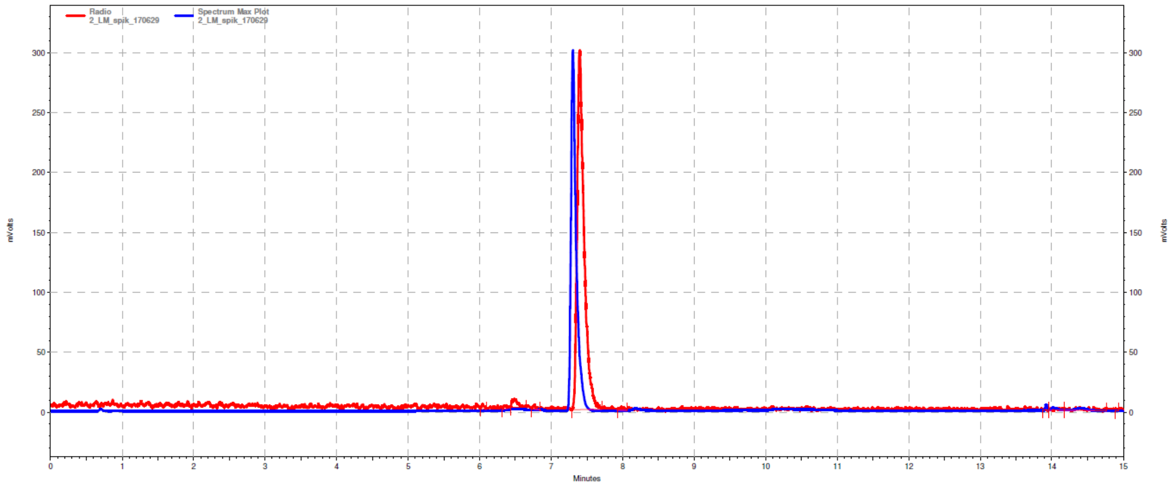

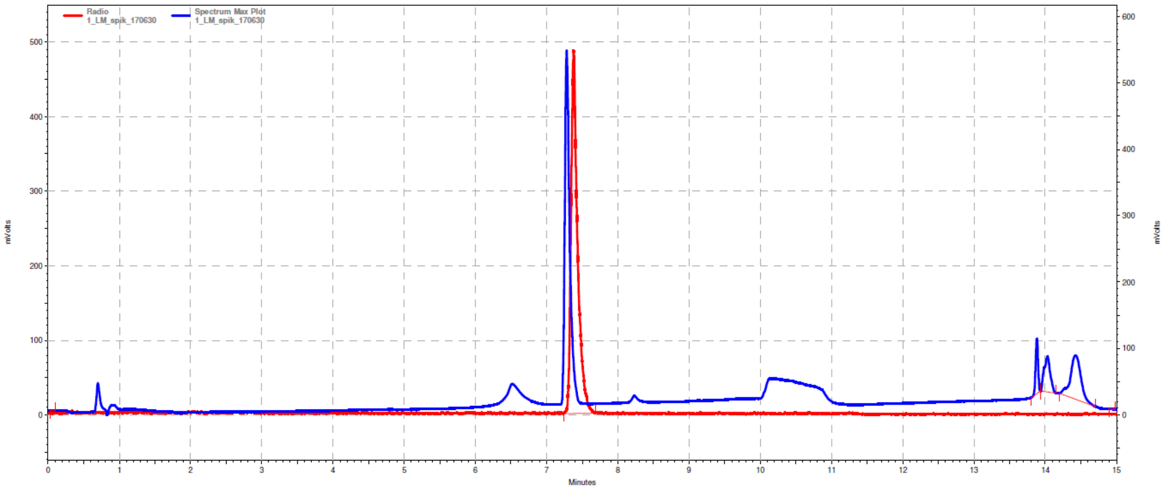

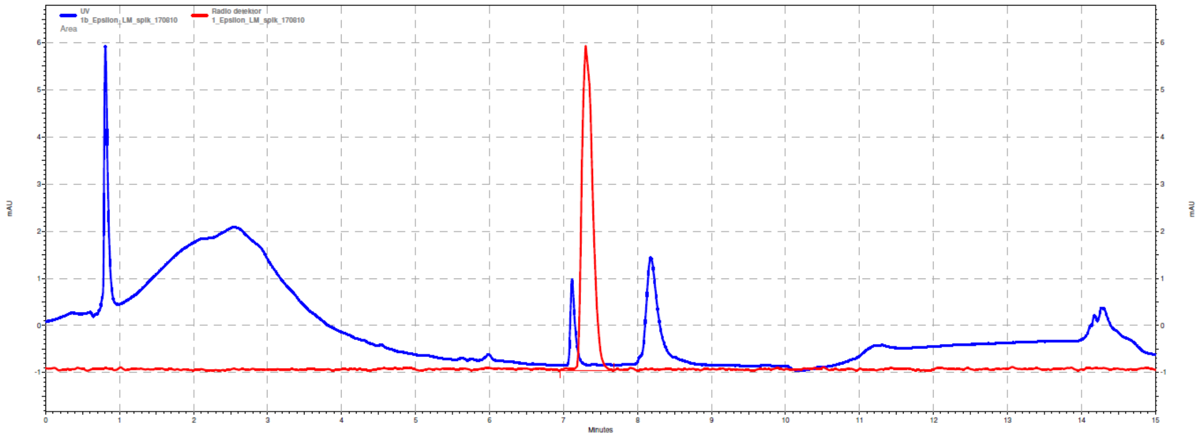

Analysis of isolated fraction containing isotopically unmodified *N*-(2,4-dichlorobenzyl)-4-phenoxypiperidine-1-carboxamide. Top: experiment 1; Middle: experiment 2; Bottom: experiment 3.

Reference list

1. Kiesewetter, D. O.; Eckelman, W. C. Utility of azetidinium methanesulfonates for radiosynthesis of 3-[18F]fluoropropyl amines *J. Label. Compd. Radiopharm.* **2004**, *47*, 953–969.
2. Guan, Z. H.; Lei, H.; Chen, M.; Ren, Z. H.; Bai, Y.; Wang, Y. Y. Palladium-catalyzed carbonylation of amines: Switchable approaches to carbamates and N,N′-disubstituted ureas *Adv. Synth. Catal.* **2012**, *354*, 489–496.
3. Paz, J.; Pérez-Balado, C.; Iglesias, B.; Muñoz, L. Carbon dioxide as a carbonylating agent in the synthesis of 2-oxazolidinones, 2-oxazinones, and cyclic ureas: Scope and limitations *J. Org. Chem.* **2010**, *75*, 3037–3046.
4. Das, S.; Addis, D.; Knöpke, L. R.; Bentrup, U.; Junge, K.; Brückner, A.; Beller, M. Selective catalytic monoreduction of phthalimides and imidazolidine-2,4-diones *Angew. Chemie - Int. Ed.* **2011**, *50*, 9180–9184.
5. Lee, S. H.; Matsushita, H.; Clapham, B.; Janda, K. D. The direct conversion of carbamates to ureas using aluminum amides *Tetrahedron* **2004**, *60*, 3439–3443.
6. Orito, K.; Miyazawa, M.; Nakamura, T.; Horibata, A.; Ushito, H.; Nagasaki, H.; Yuguchi, M.; Yamashita, S.; Yamazaki, T.; Tokuda, M. Pd (OAc) 2 -Catalyzed Carbonylation of Amines *J. Org. Chem.* **2006**, *71*, 5951–5958.
